# Exploring the mechanism of Shuangyu Granule in regulating immune-inflammatory responses in influenza through UPLC-Orbitrap-MS/MS, GC-MS, and network target analysis

**DOI:** 10.1371/journal.pone.0353259

**Published:** 2026-07-27

**Authors:** Ling Sun, ZhiTao Jiang, Ying Chen, MingShu Han, YaoZhong Lv, Liang Li, XinZhuang Zhang, Liang Cao, TuanJie Wang, ZhenZhong Wang, Wei Xiao

**Affiliations:** 1 State Key Laboratory of Technologies for Chinese Medicine Pharmaceutical Process Control and Intelligent Manufacture (Jiangsu Kanion Pharmaceutical Co., Ltd. & Nanjing University of Chinese Medicine) Jiangsu, Nanjing, China; 2 Jiangsu Kanion Pharmaceutical Co., Ltd., Lianyungang, China; Versiti Blood Research Institute, UNITED STATES OF AMERICA

## Abstract

Influenza, an acute respiratory infectious disease caused by the influenza virus, remains a significant challenge for prevention and treatment due to rapid viral mutation and high pathogenicity. Traditional Chinese Medicine (TCM), including Shuangyu Granule (SYKL), has demonstrated efficacy in managing influenza. This study aimed to systematically identify the chemical components of SYKL in vitro and its absorbed constituents in vivo, and to preliminarily explore its potential mechanism in regulating influenza-related immune inflammation. UPLC-Orbitrap-MS/MS and GC-MS were used to characterize SYKL’s chemical profile, identifying 148 in vitro components and 21 prototype absorbed blood components. Network target analysis, integrated with single-cell RNA sequencing (scRNA-seq) data from influenza patients, predicted that the absorbed components may target multiple immune-inflammatory regulatory genes across various immune cell types. Molecular docking suggested favorable predicted binding potential between these components and target proteins. Experimental validation using poly(I:C)-induced inflammatory models in both RAW264.7 macrophages and mouse bone marrow-derived macrophages (BMDMs) showed that the absorbed components—loganic acid, 8-epiloganic acid, calycosin, atractylodin, eucalyptol, secoxyloganin, and paeoniflorin—significantly reduced mRNA expression of immune-inflammatory genes (DUSP6, MAPKAPK2, NOD2) and inhibited secretion of TNF-α, IL-6, IL-8, and NO. These findings suggest that SYKL may alleviate influenza-associated inflammation through multi-component, multi-cell, and multi-target pathways, highlighting its potential in modulating excessive immune responses in influenza.

## 1. Introduction

Influenza is a highly contagious acute respiratory disease caused by the influenza virus, clinically manifested by systemic symptoms such as fever, chills, myalgia, and fatigue, along with respiratory symptoms like cough, sore throat, and rhinorrhea. It can be categorized as seasonal influenza or pandemic influenza [1]. Influenza pandemics can lead to significant global morbidity and mortality [2], while seasonal influenza causes approximately 1 billion infections and 290,000–650,000 deaths annually [3].

The influenza virus primarily infects and replicates in airway and alveolar cells, causing lung tissue damage and simultaneously triggering host innate and adaptive immune responses. From viral infection until a period after viral clearance, the body mobilizes various immune and non-immune cells to participate in virus clearance, inflammation resolution, and repair of damaged lung tissue [4–5]. Research indicates that the pathological damage caused by influenza stems more from excessive immune cell activation and the resultant immune response rather than direct viral effects [6–7]. Immune cells play a dual role in influenza progression: on one hand, innate immune cells (e.g., alveolar macrophages, neutrophils, dendritic cells, NK cells) exert early defense through phagocytosis, interferon signaling activation, and immune cell recruitment; adaptive immunity (T cells and B cells) provides long-term protection via cytotoxic effects, antibody neutralization, and immunological memory formation. On the other hand, aberrant immune cell activation leading to responses such as cytokine storms, complement system dysregulation, or delayed immune activation can cause tissue damage, potentially resulting in acute respiratory distress syndrome (ARDS) or thromboinflammation in severe cases [8–12]. For example, appropriately activated neutrophils directly kill viruses and recruit other immune cells, but overactivation can exacerbate respiratory tract damage through delayed apoptosis, release of reactive oxygen species, cytokines, and neutrophil extracellular traps (NETs) [13–15]. Alveolar macrophages can clear viruses, present antigens, and secrete anti-inflammatory factors during antiviral responses; however, overactivation can induce epithelial cell apoptosis via TRAIL (TNF-related apoptosis-inducing ligand) expression and produce nitric oxide and pro-inflammatory cytokines, aggravating tissue damage [13,16,17]. T cells also have dual roles: CD8 ⁺ cytotoxic T lymphocytes (CTLs) directly kill infected cells, while CD4 ⁺ Th1 and T follicular helper (Tfh) cells coordinate antibody production and immune memory; however, overactivation of Th17 cells can mediate IL-17-driven lung tissue inflammation, and persistent antigen stimulation may lead to T cell exhaustion, impairing viral clearance [7,8,18].

Currently approved western anti-influenza drugs are small molecule chemicals targeting viral proteins. Due to the influenza virus’s simple single-stranded RNA genome and high mutation rate, drug-resistant mutations easily arise. Resistance has been reported to varying degrees for M2 ion channel blockers (amantadine), hemagglutinin inhibitors (arbidol), neuraminidase inhibitors (oseltamivir), and RNA polymerase inhibitors (baloxavir marboxil).

Traditional Chinese Medicine (TCM) has accumulated rich theoretical and clinical experience in influenza prevention and treatment [19]. Modern research shows that Chinese herbs and their formulas can not only directly inhibit viruses but also achieve multi-level regulation by modulating body immunity and reducing excessive inflammatory responses [20–21]. Shuangyu Granule (SYKL) is composed of five medicinal herbs: Lonicerae Japonicae Flos (Jinyinhua), Houttuyniae Herba (Yuxingcao), Paeoniae Radix Rubra (Chishao), Artemisiae Argyi Folium (Aiye), and Menthae Haplocalycis Herba (Bohe). Randomized, double-blind, multicenter clinical trials have confirmed that SYKL can shorten fever duration, promote the alleviation of cough, nasal congestion, fatigue, and other symptoms, with a good safety profile [22–23]. Each individual herb has been reported to possess antiviral and immunomodulatory activities [24–28], but the material basis and immunomodulatory mechanism underlying SYKL’s overall anti-influenza effect remain unclear.

A TCM formula is a complex mixture containing hundreds of chemical constituents. However, only those components that can be absorbed into the bloodstream and reach target tissues are considered pharmacologically relevant. Therefore, identifying the absorbed components is a critical step in determining the true pharmacodynamic material basis of a TCM formula and for subsequent target prediction.

In this study, we first employed UPLC-Orbitrap-MS/MS (Ultra-high performance liquid chromatography coupled with Orbitrap tandem mass spectrometry) and GC-MS (gas chromatography-mass spectrometry) to identify the chemical composition of SYKL and its absorbed components; subsequently, network target analysis was used to predict its action targets; single-cell RNA sequencing (scRNA-seq) data from influenza patients and healthy controls were obtained from the GEO database to identify differentially expressed genes; through intersection analysis and association mapping, the potential effects of SYKL on immune cells were inferred; Furthermore, in the theoretical framework of Traditional Chinese Medicine, “holistic regulation” refers to treating the host as an integrated system, aiming to restore internal balance and strengthen vital energy (“Zheng Qi”) rather than solely targeting the pathogen. “Multi-target intervention” describes the inherent feature of TCM formulas, where multiple chemical components simultaneously interact with diverse biological pathways (e.g., inflammation, immunity) to achieve synergistic therapeutic effects, a concept that aligns with modern systems pharmacology [29–31]. Finally, this study utilized poly(I:C)-induced inflammatory models in both RAW264.7 macrophages and BMDMs—poly(I:C) is a synthetic double-stranded RNA analog that activates Toll-like receptor 3 and cytoplasmic receptors such as MDA5 and RIG-I, thereby initiating innate immune responses [32–35]—to experimentally validate part of the predictions.

## 2. Materials and methods

### 2.1. Animals and cells

Male SPF-grade SD rats, weighing 200 ± 20 g, were purchased from Sipeifu Biotechnology Co., Ltd. Male C57BL/6J mice, weighing 20–22 g, were purchased from Sipeifu (Suzhou) Biotechnology Co., Ltd. All animals were housed in an SPF-grade barrier environment (temperature 25 ± 1°C, humidity 65 ± 10%) with free access to food and water. Animals were acclimatized for one week before experiments. All animal experiments were approved by the Experimental Animal Ethics Committee of Jiangsu Kanion Pharmaceutical Co., Ltd. (KY2025022507 and KY2026023351)and conducted in accordance with the Guide for the Care and Use of Laboratory Animals. All animal procedures were performed in accordance with the ARRIVE guidelines and the Guide for the Care and Use of Laboratory Animals. For blood collection, SD rats were anesthetized with isoflurane (batch No. 2025070701, Shandong Ante Animal Husbandry Technology Co., Ltd.) using a small animal anesthesia machine (Model: TAIJI-IE, RWD) with 4% induction and 1.5% maintenance (oxygen flow rate 1.0 L/min). The depth of anesthesia was confirmed by the absence of the toe pinch reflex before any surgical procedure. Following blood collection from the abdominal aorta, exsanguination led to rapid cardiac arrest; death was confirmed by observing the absence of respiration and heartbeat for 3 minutes and bilateral pupil dilation. For bone marrow collection, C57BL/6J mice were anesthetized with isoflurane (4% induction, 2% maintenance; oxygen flow rate 0.5 L/min). After deep anesthesia (confirmed by loss of pedal reflex), both femurs and tibiae were dissected. After tissue collection, mice were euthanized by cervical dislocation under deep isoflurane anesthesia. All efforts were made to minimize animal suffering, including the use of appropriate anesthesia and rapid euthanasia procedures.RAW264.7 mouse macrophages were purchased from the Shanghai Cell Bank of the Chinese Academy of Sciences.

### 2.2. Reagents and instruments

Methanol (LC-MS grade; Merck; Germany); Acetonitrile (LC-MS grade; Merck; Germany); Formic acid (LC-MS grade; Sigma-Aldrich; USA); n-Hexane: Beijing MREDA Technology Co., Ltd.; Shuangyu Granule (provided by Jiangsu Kanion Pharmaceutical Co., Ltd.); NO detection kit, CCK-8 kit (Beyotime Biotechnology Co., Ltd., Cat. No. Z867241107, C0039); Mouse IL-6, Mouse IL-8, TNF-α ELISA kits (Bender MedSystems GmbH, Cat. No. 46461−002, 381320−003).

MiniBEST Universal RNA Extraction Kit, PrimeScript™ RT reagent Kit with gDNA Eraser, TB Green® Premix Ex Taq™ II (Takara, Japan, Lot No. AL40413A, AL61877A, ALE2092A).

UPLC-Orbitrap-MS/MS system, Xcalibur 2.2 workstation (Thermo Scientific); UPLC column: Accucore C18 column (2.1 mm × 100 mm, 2.6 µm) (Thermo Scientific); GC-MS: 7890A/7000B triple quadrupole GC-MS system with FID detector (Agilent, USA); Capillary column: Agilent DB-1 (30 m × 0.32 mm, 0.25 µm); AB StepOne Plus real-time PCR system (Applied Biosystems); FlexStation 3 multimode microplate reader (Molecular Devices); 5810R high-speed refrigerated centrifuge (Eppendorf).

### 2.3. Identification of chemical components of SYKL In Vitro and In Vivo

#### 2.3.1. Sample preparation for In Vitro SYKL Analysis.

UPLC-Orbitrap-MS/MS: An appropriate amount of SYKL powder was ground (passed through an 80-mesh sieve). 0.25 g was weighed into a stoppered conical flask, 25 mL of 70% methanol was added, and the flask was weighed. Ultrasonic extraction (250 W, 40 kHz) was performed for 30 min in a 40°C water bath. After re-weighing, the weight loss was compensated with 70% methanol. The mixture was centrifuged (11,000 rpm, 10 min), and the supernatant was filtered through a 0.22 µm nylon membrane and cooled for analysis. Before injection, the solution was diluted 15-fold with 70% methanol.

GC-MS: An appropriate amount of SYKL powder (10 g) was placed in a 500 mL flask with several glass beads, and 250 mL of water was added. Then, 3 mL of n-hexane was added through the top of a volatile oil extractor. After boiling, timing started. Heating was stopped after 60 min, and the mixture was left to stand for 30 min. The n-hexane layer was separated, and the extractor was rinsed twice with n-hexane. The washings were combined with the n-hexane layer in a 25 mL volumetric flask, diluted to volume with n-hexane, mixed well, and filtered through a 0.22 µm microporous membrane. The filtrate was used as the test solution.

#### 2.3.2. Collection of absorbed components of SYKL.

Twenty-one SD rats were randomly divided into two groups: a blank control group (n = 3) and an administration group (n = 18). The administration group received SYKL orally at a dose of 5.2 g/kg/day for 5 consecutive days, once daily. After the last administration on day 4, food was withheld but water was allowed. On day 5, after administration, the 18 rats were randomly divided into 6 subgroups (n = 3 per time point) for blood collection at 10 min, 20 min, 30 min, 1 h, 2 h, and 4 h after the final oral administration. Rats were anesthetized with isoflurane (4% induction, 1.5% maintenance; oxygen flow rate 1.0 L/min). After loss of the toe pinch reflex, the abdominal aorta was exposed via a midline incision. Blood (8–10 mL) was collected using a 10 mL syringe with a 25G needle, transferred to a heparinized tube, and centrifuged at 4°C and 4,000 rpm for 10 min. Plasma was aliquoted (0.5 mL per tube) and stored at −80°C. Following blood collection, exsanguination led to rapid cardiac arrest; death was confirmed by the absence of respiration and heartbeat for 3 min and bilateral pupil dilation.

#### 2.3.3. Sample preparation for detection of absorbed components.

UPLC-Orbitrap-MS/MS: Thawed plasma samples were brought to room temperature. Precisely 100 µL of plasma from each administered rat (total 1.8 mL) was pooled into a 5 mL EP tube and vortex-mixed. Then, 1 mL of the mixed plasma was taken, 3 volumes of chromatographic methanol were added, and the mixture was vortexed. Proteins were removed by centrifugation (4°C, 12,000 rpm, 10 min). The supernatant was collected, dried under nitrogen, and the residue was reconstituted in 200 µL of 70% methanol aqueous solution, vortexed for 3 min, centrifuged (4°C, 12,000 rpm, 10 min), and the supernatant was taken for injection.

GC-MS: Thawed plasma samples were brought to room temperature. Precisely 200 µL of plasma from each administered rat (total 3.6 mL) was pooled into a 15 mL EP tube and vortex-mixed. Then, 3 mL of the mixed plasma was taken, 1 volume of n-hexane was added, and the mixture was vortex-extracted for 10 min. The upper layer was transferred to a clean 1.5 mL centrifuge tube, centrifuged (4°C, 12,000 rpm, 10 min), and the supernatant was taken for injection.

#### 2.3.4. Instrumental analysis conditions.

UPLC-Orbitrap-MS/MS Conditions: Column: Accucore UPLC BEH C18 column (2.1 mm × 100 mm, 2.6µm); Column temperature: 40°C; Flow rate: 0.3 mL/min; Mobile phase: A (0.1% formic acid in water) – B (acetonitrile); Gradient elution: 0–5 min, 5–10% B; 5–8 min, 10–15% B; 8–14 min, 15–25% B; 14–20 min, 25–34% B; 20–26 min, 34–70% B; 26–28 min, 70–95% B; 28–28.1 min, 95–5% B; 28.1–32 min, 5% B. Ion source: Heated electrospray ionization (H-ESI); Data were acquired in positive and negative ion modes separately; Spray voltage: + 3500 V (positive), −3000 V (negative); Scan mode: Full MS/dd-MS²; Full MS resolution: 60,000; dd-MS² resolution: 15,000; Mass scan range: m/z 100–1500; Sheath gas and auxiliary gas flow rates: 50 and 10 arbitrary units, respectively; Ion transfer tube temperature: 320°C; Vaporizer temperature: 350°C; S-Lens RF Level: 70%; HCD collision energy: 30%.

GC-MS Conditions: Capillary column: Agilent DB-1 (30 m × 0.32 mm, 0.25 µm); Injection volume: 1 µL; Split ratio: 25:1; Inlet temperature: 230°C; FID detector temperature: 250°C; Nitrogen flow rate: 0.6 mL/min. Temperature program: Initial oven temperature 80°C held for 2 min, increased to 92°C at 2°C/min, then to 112°C at 10°C/min, then to 120°C at 2°C/min and held for 4 min, finally increased to 250°C at 20°C/min and held for 4 min. Ion source temperature: 260°C; Ionization voltage: −70 eV; Solvent delay: 2.5 min; Full scan MS mode; Mass range: 10–550 Da.

#### 2.3.5. Data processing.

UPLC-Orbitrap-MS/MS data were processed using Xcalibur 4.2 software. Database search parameters were set as: retention time deviation < ±1 min; mass deviation < ±5 mDa; mass range: 100–1500; target ions: [M-H]^‒^, [M+HCOO]^‒^, [M + H]^+^ and [M + NH_4_]^+^.

GC-MS data were analyzed by matching with reference standards, the NIST mass spectral library, and comparison with literature for structural confirmation.

### 2.4. Network target analysis

#### 2.4.1. Prediction of targets for SYKL absorbed components.

Targets of the absorbed components were predicted using the TCMSP, SwissTargetPrediction, and PharmMapper databases. For SwissTargetPrediction, the species was set to “

Homo sapiens”

with a probability > 0. For PharmMapper, the parameter Norm Fit was set ≥ 0.7. Predicted targets from the three databases were merged and deduplicated to obtain the target set for SYKL absorbed components.

#### 2.4.2. Acquisition of differential genes and cell cluster annotation in influenza patient peripheral blood.

Single-cell RNA sequencing data (GEO accession: GSE243629) from two influenza patients (male, 20; female, 27) and two healthy controls (male, 20; female, 29) were obtained, involving a total of 39,097 cells [36]. Data preprocessing was performed using R. Genes expressed in at least 3 cells were retained, and cells expressing at least 200 genes were kept, resulting in 39,052 cells after initial filtering. Quality control was further performed based on nFeature_RNA, nCount_RNA, and percent.mt. The expression matrix was normalized using the LogNormalize method. High-variable genes were identified using FindVariableFeatures, followed by data scaling via ScaleData. Principal component analysis (PCA) was performed, and cell clustering was conducted based on the top principal components using the FindClusters function, with UMAP for nonlinear dimensionality reduction. The FindAllMarkers function was used to identify differentially expressed genes (DEGs) for each cluster. To annotate cell types, marker genes for each cluster were identified using FindAllMarkers in Seurat and manually annotated by comparison with known cell-type-specific marker genes from the literature and the CellMarker database. All R scripts used for these analyses are provided in S4 Code.R.

#### 2.4.3. Construction of the “Absorbed Components - Differential Genes - Cells” Network for SYKL in Influenza Treatment.

To investigate the effects of SYKL on peripheral blood immune cells, the target genes of SYKL absorbed components were intersected with the DEGs from influenza patients. The “

absorbed components – differential genes”

interaction network was constructed using Cytoscape 3.9.1 software to obtain potential target genes for SYKL intervention in influenza. Based on marker genes for each immune cell type, different clusters were labeled and annotated to identify the cell types harboring the intersection targets, thereby mapping the potential effects of SYKL on scRNA-seq identified immune cells.

#### 2.4.4. Molecular Docking of “Absorbed Components - Immune-Inflammatory Regulatory Genes”.

The DEGs from influenza patients were intersected with the predicted target proteins of SYKL absorbed components. The 3D structures of the intersecting target proteins were retrieved from the PDB database (https://www.rcsb.org/). Target structures were optimized (removing water molecules and small molecule ligands) using PyMOL 2.1.0. Hydrogen atoms and charges were added using AutoDock Tools 1.5.6, and files were saved in pdbqt format. SDF format files of SYKL absorbed components were obtained from the PubChem database. Using the intersection target proteins as receptors and the corresponding absorbed components as ligands, molecular docking was performed using Vina 2.0 integrated in PyRx software to calculate binding energies and output results. PyMOL was used for result visualization.

### 2.5. Cell experiments

#### 2.5.1. Isolation and culture of mouse BMDMs.

Mice were anesthetized with isoflurane (4% induction, 2% maintenance; oxygen flow rate 0.5 L/min). Under deep anesthesia (confirmed by loss of pedal reflex), the mouse was fixed in the supine position. Both femurs and tibiae were dissected aseptically, and the epiphyses were cut off. Bone marrow was flushed with sterile saline into a 15 mL centrifuge tube, passed through a 70 μm cell strainer, and centrifuged. Red blood cell lysis buffer was used to remove erythrocytes. After another centrifugation, the pellet was resuspended in DMEM complete medium containing 20 ng/mL macrophage colony-stimulating factor (M-CSF). Cells were cultured for one week to obtain bone marrow-derived macrophages (BMDMs). After tissue collection, mice were euthanized by cervical dislocation under deep isoflurane anesthesia.

#### 2.5.2. Cell viability assessment.

RAW264.7 and BMDMs in the logarithmic growth phase were seeded into 96-well plates at a density of 8 × 10⁴ cells/mL and incubated for 24 h at 37°C, 5% CO_2_. The supernatant was discarded. A blank control group, normal control group, and drug groups were set up, with 6 replicates per group. The blank group received 100 µL medium. Drug groups received different concentrations of loganic acid, 8-epiloganic acid, calycosin, atractylodin, or eucalyptol. After 24 h incubation, CCK-8 solution was added. After 2 h incubation, absorbance at 450 nm was measured using a microplate reader. Relative cell viability was calculated. A concentration resulting in relative cell viability ≥ 90% was considered non-toxic. The maximum non-toxic concentration for each compound was determined for subsequent experiments.

#### 2.5.3. Cell modeling and treatment.

RAW264.7 and BMDMs in the logarithmic growth phase were seeded into 96-well plates at a density of 8 × 10⁴ cells/mL and incubated for 24 h at 37°C, 5% CO_2_. The supernatant was discarded. A control group, model group, and drug treatment groups were established, with 6 replicates per group. The control group received 100 µL medium. The model group was treated with 50 µg/mL poly(I:C) to establish an inflammation model mimicking viral infection. Drug treatment groups were co-treated with the maximum non-toxic doses of loganic acid, 8-epiloganic acid, calycosin, atractylodin, or eucalyptol along with 50 µg/mL poly(I:C)(The optimal modeling conditions were established through preliminary experimental optimization and are detailed in S7 Table.). After 24 h incubation, cells and supernatants were collected for subsequent analyses.

#### 2.5.4. RT-qPCR.

RT-qPCR was used to detect the effects of loganic acid, 8-epiloganic acid, calycosin, atractylodin, and eucalyptol on the expression of immune regulatory genes DUSP6, MAPKAPK2, and NOD2 in the poly(I:C)-induced RAW264.7 and BMDM inflammation model. Total RNA (500 ng) was reverse transcribed into cDNA using a Takara reverse transcription kit. Amplification was performed using the SYBR Green I fluorescence dye method on an AB StepOne Plus real-time PCR system. The cycle threshold (Ct) values were obtained. RPS18 was used as the internal reference gene for normalization. Relative gene expression was calculated using the 2^(-ΔΔCt) method. Primers were designed based on the NCBI database and synthesized by Nanjing Qingke Biotechnology Co., Ltd. Primer names and sequences are listed in Table 1.

**Table 1:** RT-qPCR Primer Sequences.

Gene	Forward (5’-3’)	Reverse (5’-3’)	Product Length (bp)
DUSP6	TTCCTCTTGAGCAGCATCGAC	AGCAAATCTCTCCCTCCGTA	133
MAPKAPK2	GCTGAAGCCCTTAGACATCACC	CCGCCTTCCCTAGACCCTC	115
NOD2	AACCAACATAATACCCCGCTCT	CAAAGGCCATGTGTGATCCCA	121
RPS18	GTGGTGTTGAGGAAAGCAGACA	TGATCACACGTTCCACCTCATC	79

#### 2.5.5. ELISA.

Invitrogen ELISA kits were used to detect the effects of loganic acid, 8-epiloganic acid, calycosin, atractylodin, and eucalyptol on the secretion levels of TNF-α, IL-6, and IL-8 in the poly(I:C)-induced RAW264.7 and BMDMs inflammation model. Cell culture supernatants from each group were used. Procedures were strictly followed according to the respective kit instructions, including incubation with standards/samples, binding with biotinylated antibodies, reaction with enzyme-conjugated secondary antibodies, and TMB substrate color development. Absorbance at 450 nm was measured using a microplate reader. Cytokine concentrations were calculated based on standard curves, and statistical analysis was performed to compare differences between groups.

#### 2.5.6. Griess assay.

A Beyotime Nitric Oxide Assay Kit (Griess method) was used to assess the effects of loganic acid, 8-epiloganic acid, calycosin, atractylodin, and eucalyptol on nitric oxide (NO) release from poly(I:C)-induced RAW264.7 and BMDMs. Cell supernatants from each group were mixed sequentially with Griess Reagent I and II, incubated at room temperature, and absorbance at 540 nm was measured. The concentration of nitrite (NO_2_^‒^) in samples was calculated using a sodium nitrite standard curve. Statistical analysis was performed to evaluate the regulatory effects of the compounds on NO production in the inflammation model.

#### 2.5.7. Statistical analysis.

Statistical analysis and graphing were performed using GraphPad Prism 10 software. Due to the standardized nature of the in vitro cell culture experiments, formal randomization was not applicable. To minimize bias, data acquisition and analysis were performed by an investigator blinded to the treatment group assignments.All experiments were performed as three independent biological replicates (n = 3), with each biological replicate consisting of three technical replicates per condition. Data are presented as mean ± standard error of the mean (SEM) from the three independent biological replicates. One-way analysis of variance (ANOVA) was used for comparisons among multiple groups. P value < 0.05 was considered statistically significant.

## 3. Results

### 3.1. Study on In Vitro chemical components and absorbed components of SYKL

**Fig 1:**
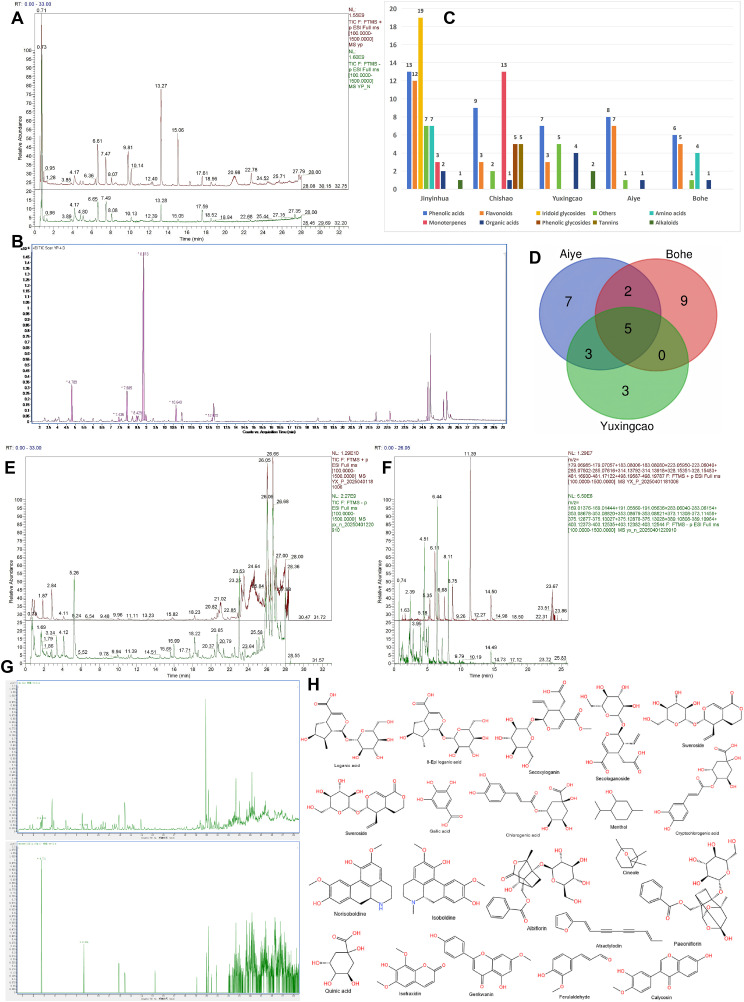
Chemical profiling of Shuangyu Granule (SYKL) in vitro and in vivo. (A) Total ion chromatograms (TIC) of in vitro chemical components of SYKL under positive (upper trace) and negative (lower trace) ionization modes by UPLC-QE-MS/MS. (B) TIC of in vitro volatile components of SYKL by GC/MS. (C) Classification and attribution of non-volatile components in SYKL in vitro. (D) Classification and attribution of volatile components in SYKL in vitro. (E) TIC of absorbed components of SYKL under positive and negative ionization modes by UPLC-QE-MS/MS. (F) Extracted ion chromatogram (EIC) of absorbed components of SYKL under positive and negative ionization modes by UPLC-QE-MS/MS. (G) TIC and EIC of absorbed components of SYKL by GC/MS. (H) Chemical structures of the 21 prototype absorbed components of SYKL.

UPLC-Orbitrap-MS/MS and GC-MS were used to identify the in vitro chemical components and absorbed components of SYKL. The total ion chromatograms (TICs) of SYKL in vitro components under positive and negative ionization modes by UPLC-Orbitrap-MS/MS are shown in Fig 1A. The TIC of SYKL in vitro components by GC/MS is shown in Fig 1B. A total of 148 in vitro chemical components were identified, including 119 non-volatile components and 29 volatile components. Identification results are listed in S1 Table and S2 Ttable. The classification and attribution of in vitro components are shown in Figs 1C and 1D. The TIC and extracted ion chromatograms (EICs) of SYKL absorbed components by UPLC-Orbitrap-MS/MS are shown in Figs 1E and 1F. The TIC and EIC of SYKL absorbed components by GC/MS are shown in Fig 1G. A total of 21 prototype absorbed components were identified, including 19 non-volatile components and 2 volatile components. Identification results are listed in S3 Table, and the chemical structures of the absorbed components are shown in Fig 1H.

### 3.2. Prediction of Targets for SYKL Absorbed Components

**Fig 2:**
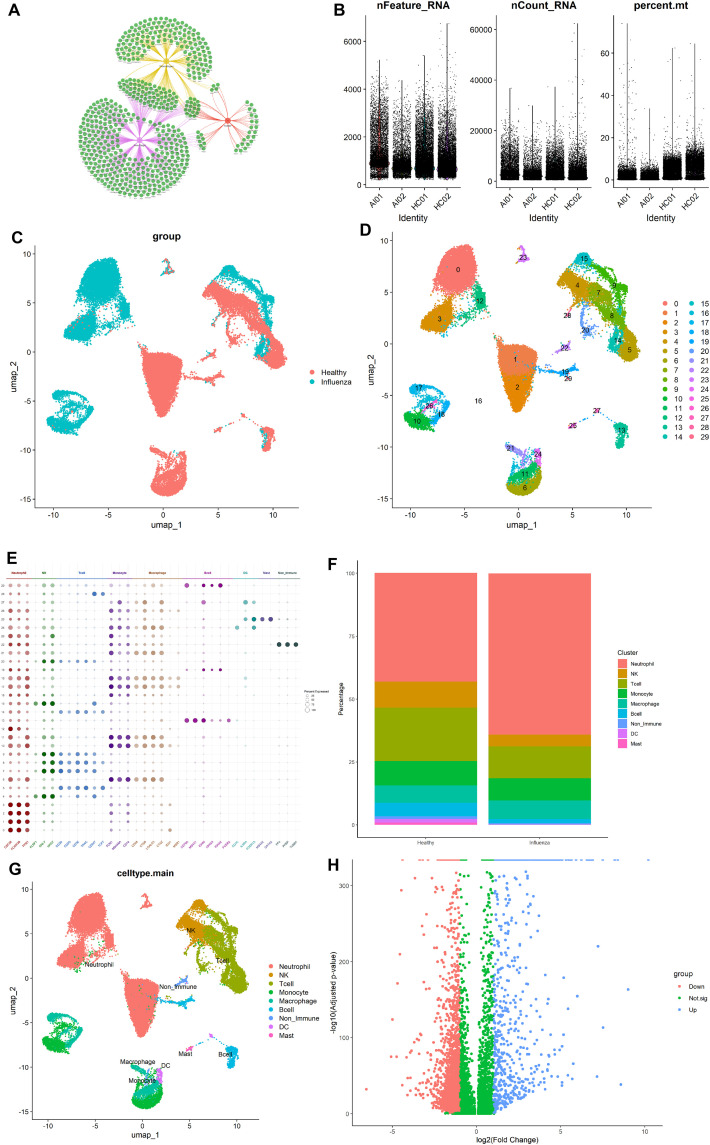
Single-cell RNA-seq analysis of peripheral blood immune cells from influenza patients and healthy controls. (A) Venn diagram showing overlapping target genes of SYKL absorbed components. (B) Quality control metrics: violin and scatter plots of nFeature_RNA, nCount_RNA, and percent.mt before and after filtering. (C) UMAP plot comparing cell distribution between influenza patients and healthy controls. (D) UMAP plot showing 30 distinct cell clusters after dimensionality reduction. (E) Expression of immune cell marker genes across clusters. (F) UMAP plot annotated with eight immune cell types. (G) Proportions of each immune cell type in patients versus controls. (H) Volcano plot of differentially expressed genes (red: upregulated; blue: downregulated).

The 21 absorbed components of SYKL were used for target prediction via the TCMSP, SwissTargetPrediction, and PharmMapper databases. After merging and deduplication, a total of 553 potential targets were obtained, as shown in the Venn network diagram (Fig 2A).

### 3.3. Acquisition of differential genes and cell cluster annotation in influenza patient peripheral blood

Quality control of scRNA-seq data from influenza patients and healthy controls was performed based on nFeature_RNA, nCount_RNA, and percent.mt. Violin plots and scatter plots of these metrics are shown in Fig 2B. Based on data distribution, quality control thresholds were set as: 200 < nFeature_RNA < 4000, nCount_RNA < 20000, percent.mt < 10%. After filtering, 36,964 cells were retained (16,801 from patients, 20,163 from controls). The UMAP plot comparing patients and controls is shown in Fig 2C. After PCA, unsupervised clustering, and UMAP dimensionality reduction, 30 clusters with distinct expression profiles were identified (Fig 2D). Using known immune cell marker genes, 8 immune cell types were identified (Fig 2E): Neutrophils (CSF3R), Natural Killer cells (KLRF1), T cells (CD247), Macrophages (MSR1), Monocytes (FCN1), B cells (IGHM), Dendritic cells (FCER1A), and Mast cells (GATA2). The UMAP plot annotated with immune cell types is shown in Fig 2F. The proportions of these immune cell types in patients and controls are shown in Fig 2G. Using the FindAllMarkers function, DEGs were identified with criteria: |log2FC| >=1 and p_val_adj <=0.05. A total of 3338 DEGs were obtained (797 upregulated, 2541 downregulated), as shown in the volcano plot (Fig 2H).

### 3.4. Network of SYKL absorbed components and influenza differential genes

**Fig 3:**
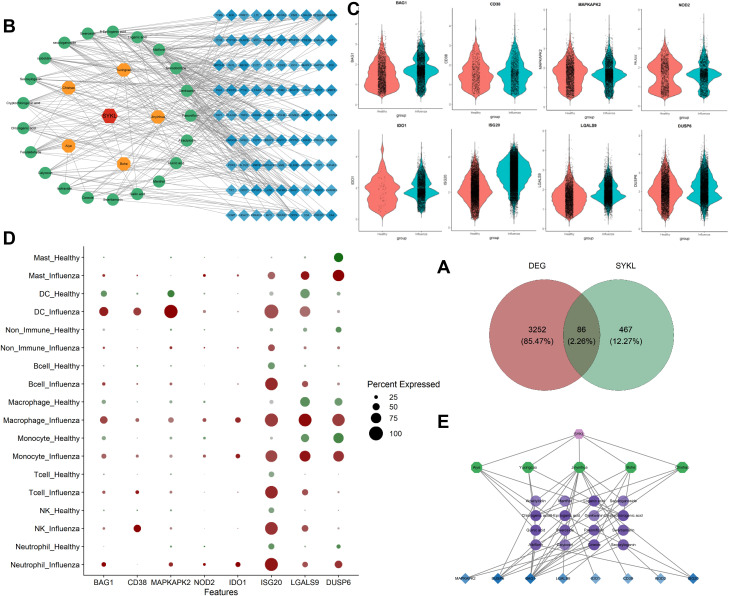
Network analysis linking SYKL absorbed components to influenza-related immune-inflammatory genes. (A) Venn diagram showing the intersection between predicted targets of SYKL components and influenza DEGs (86 common targets). (B) Interaction network of SYKL absorbed components with the 86 intersection targets. (C) Violin plots of expression distribution of eight immune-inflammatory regulatory genes (BAG1, CD38, MAPKAPK2, NOD2, IDO1, ISG20, LGALS9, DUSP6) in patients vs. controls. (D) Bubble plot of the expression levels of these genes across different immune cell types. (E) Interaction network linking SYKL absorbed components to the eight immune-inflammatory regulatory genes.

A Venn diagram shows the overlap between predicted targets of SYKL components and influenza DEGs, revealing 86 intersection targets (Fig 3A). The interaction network between SYKL absorbed components and these 86 intersection targets is shown in Fig 3B. Among the 86 intersection targets, immune-inflammatory regulatory genes (BAG1, CD38, MAPKAPK2, NOD2, IDO1, ISG20, LGALS9, DUSP6) were upregulated in influenza patients (Fig 3C). A bubble plot shows the expression levels of these genes across different immune cell types (Fig 3D). These genes were also the main targets predicted for SYKL absorbed components (Fig 3E).

### 3.5. Molecular Docking Results of SYKL Absorbed components with immune-inflammatory regulatory genes

**Fig 4:**
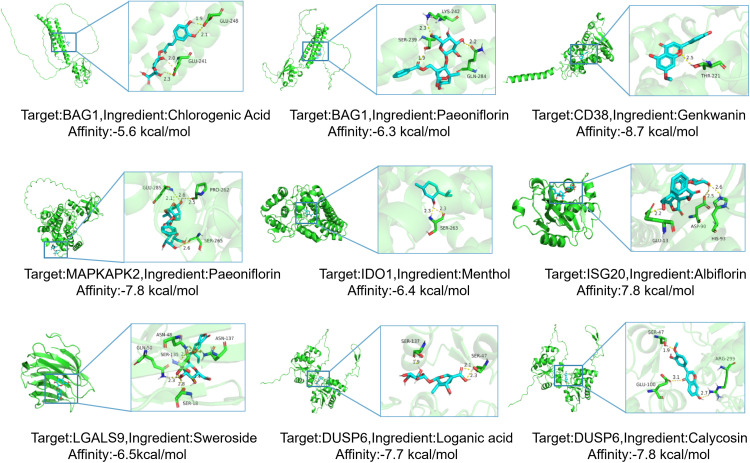
Molecular docking of SYKL absorbed components with immune-inflammatory regulatory target proteins. Representative docking diagrams of components (e.g., 8-epiloganic acid with BAG1, secoxyloganin with NOD2, paeoniflorin with MAPKAPK2) showing predicted binding modes. Binding energies are listed in Table 2.

The binding energies from molecular docking between SYKL absorbed components and immune-inflammatory regulatory genes are listed in Table 2. Lower binding energy indicates stronger and more stable binding, reflecting higher affinity. Representative docking diagrams are shown in Fig 4.

**Table 2:** Binding Energies (kcal/mol) of SYKL Absorbed Components with Immune-Inflammatory Regulatory Genes.

No.	Compound	Target	Binding Energy (kcal/mol)	No.	Compound	Target	Binding Energy (kcal/mol)
1	8-Epiloganic acid	BAG1	−5.3	14	Eucalyptol	MAPKAPK2	−5
2	Secoxyloganin	BAG1	−5.4	15	Paeoniflorin	MAPKAPK2	−7.8
3	Secoxyloganic acid	BAG1	−5.1	16	Menthol	IDO1	−6.4
4	Chlorogenic acid	BAG1	−5.6	17	Loganic acid	ISG20	−7.1
5	Loganic acid	BAG1	−5.3	18	8-Epiloganic acid	ISG20	−7.2
6	Paeoniflorin	BAG1	−6.3	19	Albiflorin	ISG20	−7.8
7	Albiflorin	BAG1	−6.1	20	Sweroside	LGALS9	−6.5
8	Cryptochlorogenic acid	BAG1	−5.6	21	Swertiamarin	LGALS9	−6.3
9	D-Quinic acid	BAG1	−5.4	22	Loganic acid	DUSP6	−7.7
10	Swertiamarin	BAG1	−5.2	23	8-Epiloganic acid	DUSP6	−7.6
11	Sweroside	BAG1	−5	24	Calycosin	DUSP6	−7.8
12	Genkwanin	CD38	−8.7	25	Atractylodin	DUSP6	−6.5
13	Secoxyloganin	NOD2	−6.7	26	Eucalyptol	DUSP6	−5.2

### 3.6. Experimental validation of selected immune-inflammatory regulatory genes

**Fig 5:**
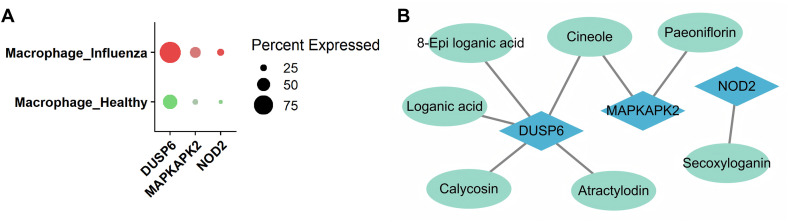
(A) Bubble plot showing expression levels of DUSP6, MAPKAPK2, and NOD2 in peripheral blood macrophages of influenza patients vs. healthy controls. (B) Network illustrating predicted interactions between the seven selected SYKL absorbed components (loganic acid, 8-epiloganic acid, calycosin, atractylodin, eucalyptol, secoxyloganin, paeoniflorin) and the three target genes DUSP6, MAPKAPK2, and NOD2.

Based on the above findings, three genes (DUSP6, MAPKAPK2, NOD2) and seven SYKL absorbed components predicted to interact with them (loganic acid, 8-epiloganic acid, calycosin, atractylodin, eucalyptol, secoxyloganin, paeoniflorin) were selected for experimental validation. A bubble plot shows the expression of DUSP6, MAPKAPK2, and NOD2 in peripheral blood macrophages of patients vs. controls (Fig 5A). The network of DUSP6, MAPKAPK2, NOD2, and the predicted SYKL absorbed components is shown in Fig 5B.

### 3.7. Effect on the relative viability of RAW264.7 and BMDMs

The relative viability of RAW264.7 and BMDMs treated for 24 h with various concentrations of loganic acid, 8-epiloganic acid, calycosin, atractylodin and eucalyptol was assessed using the CCK-8 assay. Concentrations that maintained cell viability above 90% were considered non-toxic. The maximum non-toxic concentration (MTC), defined as the highest concentration maintaining cell viability ≥90% as determined by the CCK-8 assay, was selected as the treatment concentration for the inflammation model to ensure that the observed anti-inflammatory effects were pharmacological rather than cytotoxic. The maximum non-toxic concentrations of each compound are listed in Table 3.

**Table 3:** Maximum non-toxic concentrations of compounds on RAW264.7 and BMDMs cell viability.

RAW264.7		BMDM	
Compound	Max. Non-toxic Conc.	Compound	Max. Non-toxic Conc.
Loganic acid	200 µmol/L	Loganic acid	100 µmol/L
8-epiloganic acid	200 µmol/L	8-epiloganic acid	100 µmol/L
Eucalyptol	500 µmol/L	Eucalyptol	400 µmol/L
Secoxyloganin	200 µmol/L	Secoxyloganin	100 µmol/L
Calycosin	50 µmol/L	Calycosin	25 µmol/L
Atractylodin	25 µmol/L	Atractylodin	15 µmol/L
Paeoniflorin	200 µmol/L	Paeoniflorin	100 µmol/L

### 3.8. Effects on DUSP6, MAPKAPK2, and NOD2 mRNA expression

**Fig 6:**
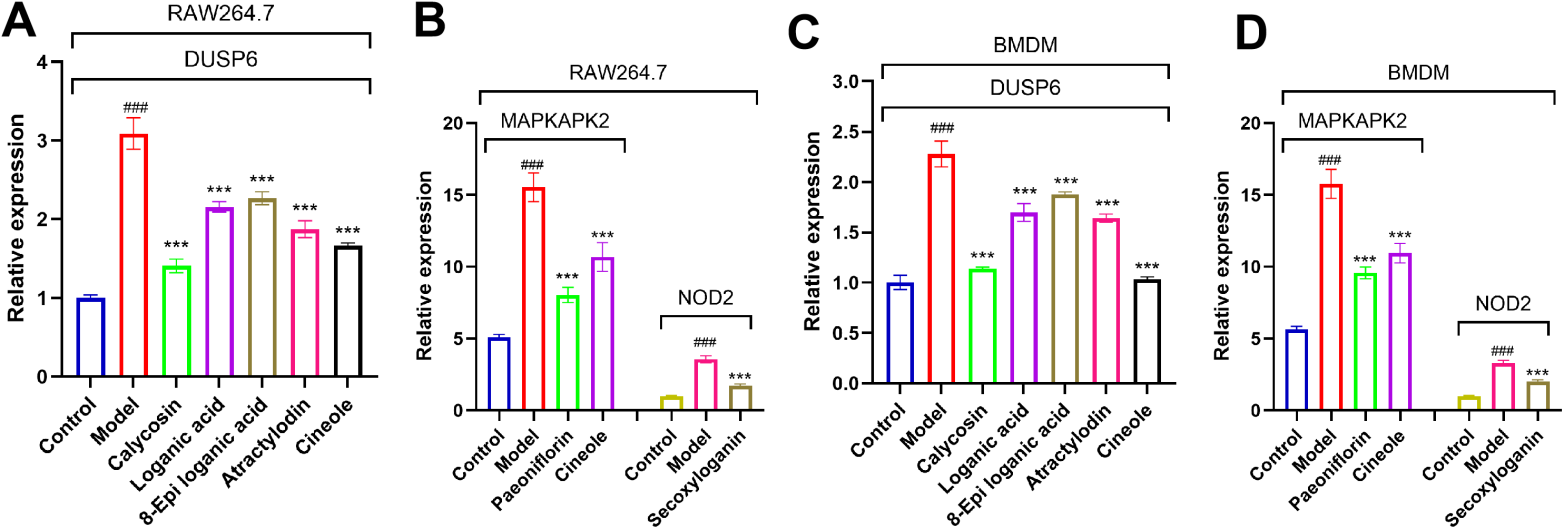
Effects of SYKL absorbed components on DUSP6, MAPKAPK2, and NOD2 mRNA expression in poly(I:C)-induced macrophages. (A) Effect on DUSP6 mRNA in RAW264.7 cells. (B) Effect on MAPKAPK2 and NOD2 mRNA in RAW264.7 cells. (C) Effect on DUSP6 mRNA in mouse bone marrow-derived macrophages (BMDMs). (D) Effect on MAPKAPK2 and NOD2 mRNA in BMDMs. All data are presented as mean ± SEM from three independent biological replicates (n = 3), each with triplicate technical replicates. Note: ###p < 0.001 vs. Control; **p < 0.01 vs. Model; ***p < 0.001 vs. Model.

Poly(I:C) significantly increased mRNA levels of DUSP6, MAPKAPK2, and NOD2 compared to the control group in both RAW264.7 (Fig 6A, B) and BMDMs (Fig 6C, D) Loganic acid, 8-epiloganic acid, calycosin, atractylodin, and eucalyptol significantly reduced DUSP6 mRNA expression. Paeoniflorin and eucalyptol significantly reduced MAPKAPK2 mRNA expression. Secoxyloganin significantly reduced NOD2 mRNA expression. The raw data are provided in S5 Table.

### 3.9. Effects on TNF-α, IL-6, IL-8 secretion and NO production

**Fig 7:**
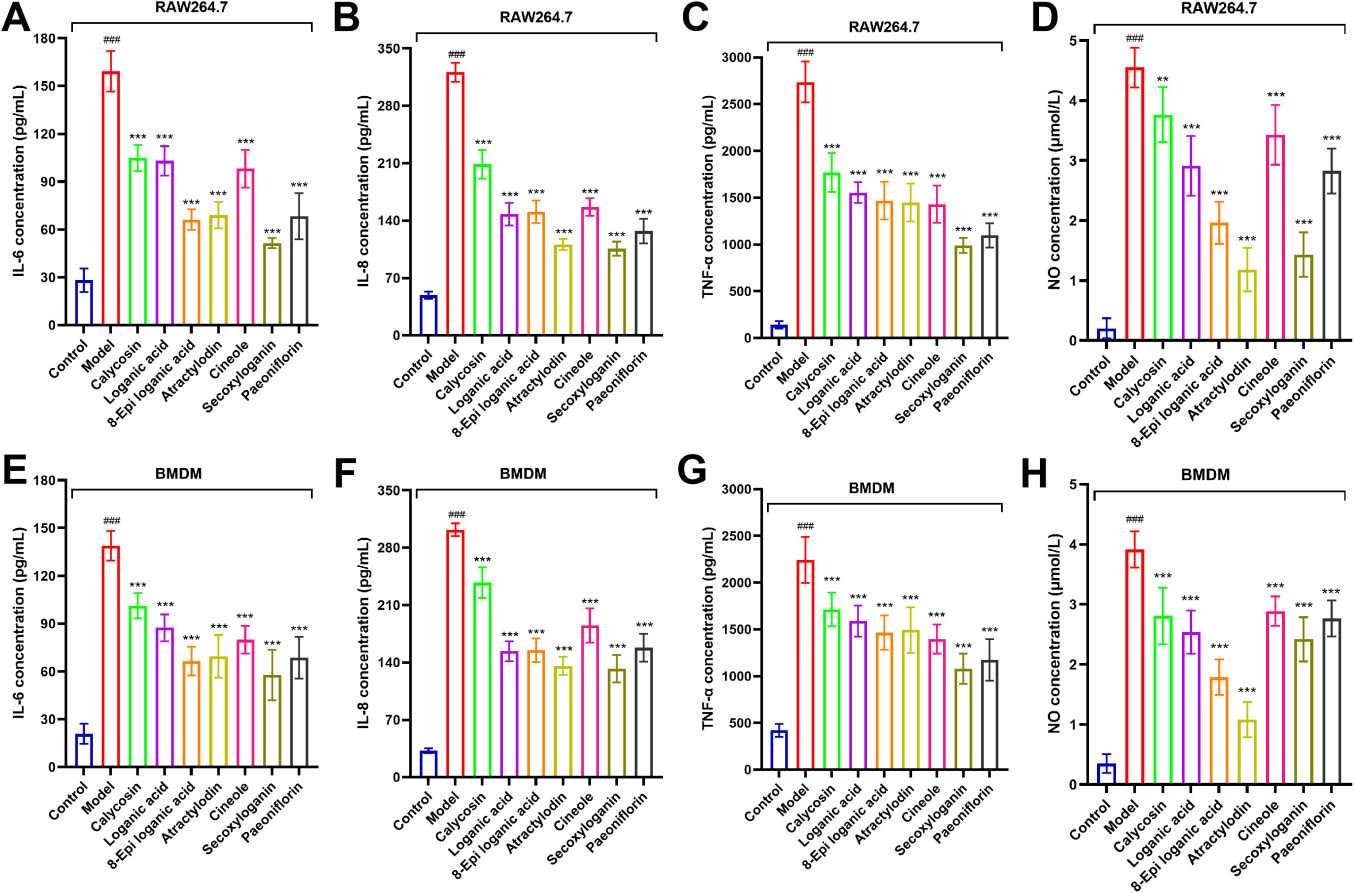
Effects of SYKL absorbed components on secretion of pro-inflammatory mediators in poly(I:C)-induced macrophages. (A-D) Effects on TNF-α, IL-6, IL-8, and NO in RAW264.7 cells. (E-H) Effects on TNF-α, IL-6, IL-8, and NO in BMDMs. All data are presented as mean ± SEM from three independent biological replicates (n = 3), each with triplicate technical replicates. Note: ###p < 0.001 vs. Control; **p < 0.01 vs. Model; ***p < 0.001 vs. Model.

Poly(I:C) successfully induced inflammation, significantly elevating TNF-α, IL-6, IL-8, and NO levels compared to the control in both RAW264.7 (Fig 7A-D) and BMDMs (Fig 7E-H). All seven tested SYKL absorbed components significantly reduced the secretion of TNF-α, IL-6, IL-8, and the production of NO compared to the model group. The raw data are available in S6 Table.

## 4. Discussion

This study integrated UPLC-Orbitrap-MS/MS, GC-MS, network target analysis, single-cell RNA sequencing, and cell experiments to preliminarily reveal the multi-component, multi-cell, multi-target mechanism by which SYKL regulates influenza immune inflammation. A total of 148 in vitro chemical components (119 non-volatile, 29 volatile) and 21 prototype absorbed components (19 non-volatile, 2 volatile) of SYKL were identified. Network target analysis predicted that the 21 absorbed components may participate in regulating the function of 8 types of immune cells in the peripheral blood of influenza patients. Focusing on macrophages, a key innate immune cell type, this study used poly(I:C)-induced inflammatory models in both RAW264.7 and BMDMs to validate the significant anti-inflammatory effects of the absorbed components loganic acid, 8-epiloganic acid, calycosin, atractylodin, eucalyptol, secoxyloganin, and paeoniflorin. These components not only inhibited the mRNA expression of key immune regulatory genes DUSP6, MAPKAPK2, and NOD2 in both cell types but also reduced the secretion of pro-inflammatory mediators NO, TNF-α, IL-6, and IL-8, thereby alleviating excessive inflammatory responses.

Mechanistically, MAPKAPK2 is a key kinase downstream of the p38 MAPK signaling pathway. It stabilizes p38 MAPK activity, forming a positive feedback loop that enhances inflammatory signal transduction, participates in regulating multiple signaling pathways involved in inflammation, and is a key factor controlling the synthesis and release of pro-inflammatory proteins such as TNF-α, IL-6, and IL-1β [37–40]. DUSP6 is an ERK-specific phosphatase primarily negatively regulating ERK1/2 activity. The ERK pathway is involved in inflammatory cytokine expression, cell proliferation, and survival in immune cells. DUSP6 expression is induced by ERK signaling, forming a negative feedback loop that limits excessive or sustained ERK activation, thereby indirectly affecting the intensity and duration of inflammatory responses [41–42]. NOD2 plays a crucial immunomodulatory role in influenza virus infection: it recognizes viral RNA and induces type I interferon via the MAVS-IRF3 axis to initiate early antiviral responses; it also promotes dendritic cell activation and virus-specific CD8 ⁺ T cell proliferation to clear infected cells. Furthermore, NOD2 can induce mitophagy, inhibiting excessive inflammasome activation and reducing lung immunopathology. Studies show that its ligand MDP can enhance these protective responses, reducing viral load and improving outcomes [43–44]. Therefore, NOD2 acts both as a sensor for influenza virus recognition and initiation of adaptive immunity, and as a key regulator balancing antiviral defense and inflammatory injury. This study showed that SYKL absorbed components significantly downregulated the expression of these genes, suggesting that SYKL may balance immune responses and inhibit excessive inflammatory damage such as cytokine storms by modulating MAPK and NOD-like receptor-related pathways.

To further corroborate the anti-inflammatory effects of the seven SYKL absorbed components investigated in this study, we reviewed the relevant literature. Accumulating evidence indicates that these seven components exhibit clear anti-inflammatory activities across various inflammation models: loganic acid acts via inhibition of the TLR4/NF-κB pathway [45–46]; eucalyptol alleviates pulmonary inflammation by suppressing TLR4/NF-κB pathway activation and ameliorating oxidative stress [47–48]; calycosin attenuates acute lung injury by regulating the miR-375-3p/ROCK2 axis, blocking the HMGB1/MyD88/NF-κB pathway, and inhibiting NLRP3 inflammasome activation [49–52]; paeoniflorin exerts lung-protective effects by inhibiting p38 MAPK/JNK phosphorylation and NF-κB activation [53]; atractylodin blocks the p38 MAPK, ERK, and NF-κB pathways, exhibiting systemic anti-inflammatory effects [54–55]; secoxyloganin dose-dependently inhibits NO release [56]; and 8-epiloganic acid exerts anti-inflammatory effects through inhibition of the TLR/MyD88/NF-κB, NLRP3, and JAK-STAT signaling pathways [57]. These literature findings support our experimental results from multiple perspectives and further confirm that these seven components constitute an important pharmacodynamic material basis for the anti-influenza immune-regulatory effects of SYKL.

These findings align with TCM theories of “holistic regulation” and “multi-target intervention”. SYKL consists of five herbs: Lonicerae Japonicae Flos, Houttuyniae Herba, Paeoniae Radix Rubra, Artemisiae Argyi Folium, and Menthae Haplocalycis Herba. Previous studies suggest each herb possesses antiviral and immunomodulatory activities. This study is the first to systematically reveal, from a pharmacologically relevant (absorbed components) perspective, the material basis for SYKL’s immunomodulatory effects using a systems pharmacology approach. Both predictions and experiments suggest that SYKL’s action does not rely on a single component or target, but rather involves multiple active components synergistically regulating multiple immune-related signaling pathways (e.g., MAPK, NOD-like receptor pathways), thereby balancing immune responses and inhibiting excessive inflammatory damage like “cytokine storm”, which may underlie its clinical efficacy in alleviating influenza symptoms.

Notably, this study not only validated the direct inhibitory effects of SYKL on macrophage inflammation models but also predicted its potential regulatory effects on various immune cells like neutrophils, T cells, B cells, and NK cells through network analysis, reflecting the broad scope of SYKL’s immunomodulatory action. Molecular docking results further theoretically support the favorable predicted binding potential between absorbed components and key target proteins (e.g., BAG1, CD38, DUSP6), providing clues for future in-depth investigation of the mechanisms.It should be emphasized that molecular docking was used solely as a hypothesis-generating computational tool, and its results require further experimental validation.

This study has limitations. First, the scRNA-seq dataset includes only 2 influenza patients and 2 healthy controls (totaling 39,097 cells). The limited donor-level sample size reduces statistical power and may introduce inter-individual variability; therefore, these results should be considered exploratory and hypothesis-generating, and they await validation in larger independent cohorts.; Second, the study primarily focused on innate immune responses, especially macrophages; the effects on adaptive immune cells (e.g., T, B cells) have not been experimentally validated. Third, cell experiments are in vitro models and cannot fully replicate the complex in vivo immune microenvironment and inter-organ interactions. Fourth, no in vivo disease model was employed; this is a key direction for our ongoing and future studies.

In summary, this study employed a systematic strategy of “chemical basis identification – network target prediction – cell experiment validation” to preliminarily elucidate the potential mechanism by which Shuangyu Granule (SYKL) regulates influenza immune inflammation. The research indicates that SYKL contains multiple active components that can be absorbed into the bloodstream. These components act on multiple immune-related targets, regulating the function of various immune cells, including macrophages, thereby inhibiting excessive inflammatory responses and alleviating immune pathological damage caused by influenza. This study not only offers preliminary evidence supporting the potential clinical application of SYKL in influenza treatment but also offers a referable approach for investigating the multi-component, multi-cell, multi-target mechanism of action of TCM formulas. Further in vivo experiments and clinical studies are needed to comprehensively evaluate the immunomodulatory efficacy of SYKL and its application value in influenza prevention and treatment.

## Supporting information

S1 Table The in vitro chemical constituents of SYKL were identified using UPLC-Orbitrap-MS/MS. (DOCX)

S2 Table The in vitro chemical constituents of SYKL were identified using GC-MS. (DOCX)

S3 Table The absorbed components of Shuangyu Keli in blood were identified using UPLC-Q-E-MS/MS and GC-MS. (DOCX)

S4 Code.R Code.R script used for single-cell RNA-seq data preprocessing, quality control, dimensionality reduction, clustering, and differential expression analysis. (DOCX)

S5 Table Raw qPCR data including Ct values, normalization to RPS18, and relative expression levels (2^(-ΔΔCt)) from three independent biological replicates. (XLSX)

S6 Table Raw ELISA data including standard curves, optical density (OD) values, and calculated concentrations of TNF-α, IL-6, and IL-8, as well as Griess assay data for NO, from three independent biological replicates. (XLSX)

S7 Table Optimization of poly(I:C) concentration and cell seeding density for establishing inflammation model in RAW264.7 macrophages and BMDMs. (XLSX)

S1 Fig Graphical Abstract. (TIF)

## References

[1] Brody H. Influenza. Nat. 2019;573(7774):S49. 10.1038/d41586-019-02750-x 31534258

[2] Wendel I, Matrosovich M, Klenk HD. SnapShot: Evolution of human influenza A viruses. Cell Host Microbe. 2015;17(3):416-416.e1. doi: 10.1016/j.chom.2015.02.001 25766297

[3] World Health Organization. Global Influenza Programme. 2017.

[4] Herold S, Becker C, Ridge KM, Budinger GRS. Influenza virus-induced lung injury: pathogenesis and implications for treatment. Eur Respir J. 2015;45(5):1463–78. doi: 10.1183/09031936.00186214 25792631

[5] Wei X, et al. Host recovery from respiratory viral infection. Annu Rev Immunol. 2023;41:277–300. 36716750 10.1146/annurev-immunol-101921-040450

[6] Oshansky CM, Gartland AJ, Wong S-S, Jeevan T, Wang D, Roddam PL, et al. Mucosal immune responses predict clinical outcomes during influenza infection independently of age and viral load. Am J Respir Crit Care Med. 2014;189(4):449–62. doi: 10.1164/rccm.201309-1616OC 24308446 PMC3977720

[7] Newton AH, Cardani A, Braciale TJ. The host immune response in respiratory virus infection: balancing virus clearance and immunopathology. Semin Immunopathol. 2016;38(4):471–82. doi: 10.1007/s00281-016-0558-0 26965109 PMC4896975

[8] Wong SS. Severe influenza is characterized by prolonged immune activation: results from the SHIVERS cohort study. J Infect Dis. 2018;217(2):245–56. 29112724 10.1093/infdis/jix571 PMC7335675

[9] Garcinuño S. Immune dysregulation is an important factor in the underlying complications in influenza infection. Front Immunol. 2024;15:1443096. 39176097 10.3389/fimmu.2024.1443096 PMC11339618

[10] Liang Y. Pathogenicity and virulence of influenza. Virulence. 2023;14(1):2223057. doi: 10.1080/21505594.2023.2223057 37339323 PMC10283447

[11] Mifsud EJ, Kuba M, Barr IG. Innate Immune Responses to Influenza Virus Infections in the Upper Respiratory Tract. Viruses. 2021;13(10):2090. doi: 10.3390/v13102090 34696520 PMC8541359

[12] Jiang H, Zhang Z. Immune response in influenza virus infection and modulation of immune injury by viral neuraminidase. Virol J. 2023;20(1):193. doi: 10.1186/s12985-023-02164-2 37641134 PMC10463456

[13] Short KR, Kroeze EJBV, Fouchier RAM, Kuiken T. Pathogenesis of influenza-induced acute respiratory distress syndrome. Lancet Infect Dis. 2014;14(1):57–69. doi: 10.1016/S1473-3099(13)70286-X 24239327

[14] Matute-Bello G, Liles WC, Radella F 2nd, Steinberg KP, Ruzinski JT, Jonas M, et al. Neutrophil apoptosis in the acute respiratory distress syndrome. Am J Respir Crit Care Med. 1997;156(6):1969–77. doi: 10.1164/ajrccm.156.6.96-12081 9412582

[15] Narasaraju T, Yang E, Samy RP, Ng HH, Poh WP, Liew A-A, et al. Excessive neutrophils and neutrophil extracellular traps contribute to acute lung injury of influenza pneumonitis. Am J Pathol. 2011;179(1):199–210. doi: 10.1016/j.ajpath.2011.03.013 21703402 PMC3123873

[16] Högner K, Wolff T, Pleschka S, Plog S, Gruber AD, Kalinke U, et al. Macrophage-expressed IFN-β contributes to apoptotic alveolar epithelial cell injury in severe influenza virus pneumonia. PLoS Pathog. 2013;9(2):e1003188. doi: 10.1371/journal.ppat.1003188 23468627 PMC3585175

[17] Weaver JJA, Smith AM. Quantitatively Mapping Immune Control during Influenza. Curr Opin Syst Biol. 2024;38:100516. doi: 10.1016/j.coisb.2024.100516 39430368 PMC11488648

[18] Yunis J, Short KR, Yu D. Severe respiratory viral infections: T-cell functions diverging from immunity to inflammation. Trends Microbiol. 2023;31(6):644–56. doi: 10.1016/j.tim.2022.12.008 36635162 PMC9829516

[19] Liu QQ, et al. Clinical Practice Guideline for Influenza Treatment with Traditional Chinese Medicine. Zhong Yi Za Zhi. 2022;63(01):85–98.

[20] Yang M, Wang Y, Yue Y, Liang L, Peng M, Zhao M, et al. Traditional Chinese medicines as effective agents against influenza virus-induced pneumonia. Biomed Pharmacother. 2022;153:113523. doi: 10.1016/j.biopha.2022.113523 36076605

[21] Liu L. Research progress on anti-influenza virus effects of TCM active components and formulas by regulating innate immunity. Zhong Cheng Yao. 2025;47(05):1564–9.

[22] Lin X, et al. Fingerprint and quantitative analysis of volatile components in Shuangyu granules. Zhong Cao Yao. 2019;50(09):2081–6.

[23] Xi ZY, et al. Randomized, double-blind, multicenter efficacy observation of Shuangyu granules in treating influenza (wind-heat invading the defense syndrome). Xian Dai Yao Wu Yu Lin Chuang. 2025;40(01):128–33.

[24] Gao J. Research progress on chemical constituents, pharmacological effects and modern application of Lonicerae Japonicae Flos. Zhong Guo Shi Yan Fang Ji Xue Za Zhi. 2025;1–15.

[25] Ni JT. Research progress on the material basis and mechanism of anti-inflammatory activity of Paeoniae Radix Rubra. Zhong Yi Yao Xin Xi. 2024;41(02):87–93.

[26] Wang LR. Research progress on Houttuyniae herba and prediction of its quality markers. Zhong Yao Xin Yao Yu Lin Chuang Yao Li. 2024;35(07):1084–92.

[27] Sun WH, et al. Research progress on pharmacological effects of active components from Menthae haplocalycis herba. Jiang Su Zhong Yi Yao. 2023;55(05):78–82.

[28] Zhang XX, et al. Research progress on flavonoids in Artemisiae Argyi folium. Zhong Guo Zhong Yi Yao Xian Dai Yuan Cheng Jiao Yu. 2025;23(07):187–9.

[29] Xiao M, Huang T, Qin S, Zhang T, Li R, Zhao X, et al. Anti-influenza formula medicines based on warm disease theory of traditional Chinese medicine. Chin Herb Med. 2026;18(2):231–40. doi: 10.1016/j.chmed.2026.02.008 41971581 PMC13069622

[30] Bai Y, Liu T, Zhang S, Shi Y, Yang Y, Ding M, et al. Traditional Chinese Medicine for Viral Pneumonia Therapy: Pharmacological Basis and Mechanistic Insights. Int J Biol Sci. 2025;21(3):989–1013. doi: 10.7150/ijbs.105086 39897040 PMC11781171

[31] Yang M, Wang Y, Yue Y, Liang L, Peng M, Zhao M, et al. Traditional Chinese medicines as effective agents against influenza virus-induced pneumonia. Biomed Pharmacother. 2022;153:113523. doi: 10.1016/j.biopha.2022.113523 36076605

[32] Yao Z, Liang Z, Li M, Wang H, Ma Y, Guo Y, et al. Aluminum oxyhydroxide-Poly(I:C) combination adjuvant with balanced immunostimulatory potentials for prophylactic vaccines. J Control Release. 2024;372:482–93. doi: 10.1016/j.jconrel.2024.06.054 38914205

[33] Liu L, Wang D, Fu Y, Duan Z, Adetula AA, Liu H, et al. Transcriptional analyses provide novel insights into the transgenerational effects of Poly (I:C) on chickens. Ecotoxicol Environ Saf. 2022;247:114216. doi: 10.1016/j.ecoenv.2022.114216 36288637

[34] Singh V, Chernatynskaya A, Qi L, Chuang H-Y, Cole T, Jeyalatha VM, et al. Liposomes-Encapsulating Double-Stranded Nucleic Acid (Poly I:C) for Head and Neck Cancer Treatment. ACS Pharmacol Transl Sci. 2024;7(5):1612–23. doi: 10.1021/acsptsci.4c00121 38751634 PMC11092114

[35] Lamoot A, Jangra S, Laghlali G, Warang P, Singh G, Chang LA, et al. Lipid Nanoparticle Encapsulation Empowers Poly(I:C) to Activate Cytoplasmic RLRs and Thereby Increases Its Adjuvanticity. Small. 2024;20(10):e2306892. doi: 10.1002/smll.202306892 37867244 PMC7617129

[36] Zhang Y, Zong L, Zheng Y, Zhang Y, Li N, Li Y, et al. A single-cell atlas of the peripheral immune response in patients with influenza A virus infection. iScience. 2023;26(12):108507. doi: 10.1016/j.isci.2023.108507 38089584 PMC10711475

[37] Ronkina N, Menon MB, Schwermann J, Tiedje C, Hitti E, Kotlyarov A, et al. MAPKAP kinases MK2 and MK3 in inflammation: complex regulation of TNF biosynthesis via expression and phosphorylation of tristetraprolin. Biochem Pharmacol. 2010;80(12):1915–20. doi: 10.1016/j.bcp.2010.06.021 20599781

[38] Fiore M. Targeting mitogen-activated protein kinase-activated protein kinase 2 (MAPKAPK2, MK2): medicinal chemistry efforts to lead small molecule inhibitors to clinical trials. J Med Chem. 2016;59(8):3609–34. 26502061 10.1021/acs.jmedchem.5b01457 PMC4850115

[39] Gurgis FMS, Ziaziaris W, Munoz L. Mitogen-activated protein kinase-activated protein kinase 2 in neuroinflammation, heat shock protein 27 phosphorylation, and cell cycle: role and targeting. Mol Pharmacol. 2014;85(2):345–56. doi: 10.1124/mol.113.090365 24296859

[40] Gorska MM. MK2 controls the level of negative feedback in the NF-kappaB pathway and is essential for vascular permeability and airway inflammation. J Exp Med. 2007;204(7):1637–52. 17576778 10.1084/jem.20062621 PMC2118652

[41] Caunt CJ, Keyse SM. Dual-specificity MAP kinase phosphatases (MKPs): shaping the outcome of MAP kinase signalling. FEBS J. 2013;280(2):489–504. doi: 10.1111/j.1742-4658.2012.08716.x 22812510 PMC3594966

[42] Ekerot M, Stavridis MP, Delavaine L, Mitchell MP, Staples C, Owens DM, et al. Negative-feedback regulation of FGF signalling by DUSP6/MKP-3 is driven by ERK1/2 and mediated by Ets factor binding to a conserved site within the DUSP6/MKP-3 gene promoter. Biochem J. 2008;412(2):287–98. doi: 10.1042/BJ20071512 18321244 PMC2474557

[43] Egarnes B, Gosselin J. Contribution of Regulatory T Cells in Nucleotide-Binding Oligomerization Domain 2 Response to Influenza Virus Infection. Front Immunol. 2018;9:132. doi: 10.3389/fimmu.2018.00132 29445379 PMC5797787

[44] Trindade BC, Chen GY. NOD1 and NOD2 in inflammatory and infectious diseases. Immunol Rev. 2020;297(1):139–61. doi: 10.1111/imr.12902 32677123 PMC8928416

[45] Prakash AN, Prasad N, Puppala ER, Panda SR, Jain S, Ravichandiran V, et al. Loganic acid protects against ulcerative colitis by inhibiting TLR4/NF-κB mediated inflammation and activating the SIRT1/Nrf2 anti-oxidant responses in-vitro and in-vivo. Int Immunopharmacol. 2023;122:110585. doi: 10.1016/j.intimp.2023.110585 37421777

[46] Aktar A, Bhuia S, Chowdhury R, Ferdous J, Khatun M, Hasan SA, et al. An Insight of Plant Source, Toxicological Profile, and Pharmacological Activities of Iridoid Loganic Acid: A ComprehensiveReview. Chem Biodivers. 2024;21(12):e202400874. doi: 10.1002/cbdv.202400874 39113595

[47] Zhao C, Sun J, Fang C, Tang F. 1,8-cineol attenuates LPS-induced acute pulmonary inflammation in mice. Inflammation. 2014;37(2):566–72. doi: 10.1007/s10753-013-9770-4 24197825

[48] Kennedy-Feitosa E, Okuro RT, Pinho Ribeiro V, Lanzetti M, Barroso MV, Zin WA, et al. Eucalyptol attenuates cigarette smoke-induced acute lung inflammation and oxidative stress in the mouse. Pulm Pharmacol Ther. 2016;41:11–8. doi: 10.1016/j.pupt.2016.09.004 27599597

[49] Chen G, Hou Y, Li X, Pan R, Zhao D. Sepsis-induced acute lung injury in young rats is relieved by calycosin through inactivating the HMGB1/MyD88/NF-κB pathway and NLRP3 inflammasome. Int Immunopharmacol. 2021;96:107623. doi: 10.1016/j.intimp.2021.107623 33857805

[50] Zhu C-J, Yang W-G, Li D-J, Song Y-D, Chen S-Y, Wang Q-F, et al. Calycosin attenuates severe acute pancreatitis-associated acute lung injury by curtailing high mobility group box 1 - induced inflammation. World J Gastroenterol. 2021;27(44):7669–86. doi: 10.3748/wjg.v27.i44.7669 34908806 PMC8641048

[51] Yao J, et al. Calycosin attenuates lipopolysaccharide-induced acute lung injury in mice through the miR-375-3p/ROCK2 axis. J Invest Surg. 2023;36(1):2211166. 37400250 10.1080/08941939.2023.2211166

[52] Xia Y, Cao Y, Sun Y, Hong X, Tang Y, Yu J, et al. Calycosin Alleviates Sepsis-Induced Acute Lung Injury via the Inhibition of Mitochondrial ROS-Mediated Inflammasome Activation. Front Pharmacol. 2021;12:690549. doi: 10.3389/fphar.2021.690549 34737695 PMC8560711

[53] Zhou H, Bian D, Jiao X, Wei Z, Zhang H, Xia Y, et al. Paeoniflorin protects against lipopolysaccharide-induced acute lung injury in mice by alleviating inflammatory cell infiltration and microvascular permeability. Inflamm Res. 2011;60(10):981–90. doi: 10.1007/s00011-011-0359-9 21744312

[54] Qu L, Lin X, Liu C, Ke C, Zhou Z, Xu K, et al. Atractylodin Attenuates Dextran Sulfate Sodium-Induced Colitis by Alleviating Gut Microbiota Dysbiosis and Inhibiting Inflammatory Response Through the MAPK Pathway. Front Pharmacol. 2021;12:665376. doi: 10.3389/fphar.2021.665376 34335244 PMC8320761

[55] Chuang CH, Cheng Y-C, Lin S-C, Lehman CW, Wang S-P, Chen D-Y, et al. Atractylodin Suppresses Dendritic Cell Maturation and Ameliorates Collagen-Induced Arthritis in a Mouse Model. J Agric Food Chem. 2019;67(24):6773–84. doi: 10.1021/acs.jafc.9b01163 31154759

[56] Jiang XH, et al. Chemical constituents from the stems and leaves of Lonicera hypoglauca and their anti-inflammatory activities. Zhong Cheng Yao (In Chinese). 2024;46(02):484–9.

[57] Guo MX. Pharmacological material basis and molecular mechanism of Lagotis brachystachya in the treatment of chronic alcoholic liver injury combined with gouty arthritis D. Jiangxi University of Chinese Medicine. 2023.

