# Supplementary material for: Exploring the mechanism of Shuangyu Granule in regulating immune-inflammatory responses in influenza through UPLC-Orbitrap-MS/MS, GC-MS, and network target analysis
Source: PLoS One. 2026 Jul 27;21(7):e0353259. doi: 10.1371/journal.pone.0353259 (PMC13405112; doi:10.1371/journal.pone.0353259)
Supplement: S1 Table — (DOCX) [file pone.0353259.s001.docx]

| The in vitro chemical constituents of SYKL were identified using UPLC-Orbitrap-MS/MS | | | | | | | | | | | |
| --- | --- | --- | --- | --- | --- | --- | --- | --- | --- | --- | --- |
| **No.** | **Identification** | **tR** | **Formulas** | **Selected ion** | **Measured** | **ppm** | **Fragmentation** | **Source** | **Types** | **Remarks** | **Ref** |
| 1 | Arginine | 0.65 | C6H14N4O2 | [M+H]+ | 175.11871 | -1.383059897 | 158.09215、130.09747、116.07053 | Jinyinhua | Amino Acids | / | Li Panlin, Li Chuyuan, Liu Menghua, et al. Comparison of Chemical Constituents between Honeysuckle and Lonicerae Flos Based on UFLC-Triple-Q-TOF-MS/MS Technology [J]. Central South Pharmacy, 2016, 14(04): 363-369. |
| 2 | Fructose | 0.68 | [C6H12O6](https://pubchem.ncbi.nlm.nih.gov/#query=C6H12O6) | [M-H]- | 179.0562 | 6.620870146 | 161.04568、89.02453、87.00880、71.01389、59.01386 | Jinyinhua | Others | / | Duan Huifang. Research on the Quality Standard for the Production of Honeysuckle, the Raw Material of Reduning Injection [D]. Nanjing University of Chinese Medicine, 2020. DOI:10.27253/d.cnki.gnjzu.2020.000111. |
| 3 | Proline | 0.71 | C5H9NO2 | [M+H]+ | 116.07046 | -1.250101177 | 116.07047、70.06503 | Jinyinhua、Bohe | Amino Acids | / | Chen Qiulin, Yao Cheng. Determination of Amino Acids in the Chinese Medicine Mentha Herb [J]. Journal of Nanjing Institute of Physical Education (Natural Science Edition), 2003, (04): 60-61+56. |
|  |  |  | C5H9NO2 | [M+H]+ | 116.07046 | -1.250101177 | 116.07047、70.06503 | Jinyinhua、Bohe | Amino Acids | / | Cai Z, Liao H, Wang C, Chen J, Tan M, Mei Y, Wei L, Chen H, Yang R, Liu X. A comprehensive study of the aerial parts of Lonicera japonica Thunb. based on metabolite profiling coupled with PLS-DA. Phytochem Anal. 2020 Nov;31(6):786-800. doi: 10.1002/pca.2943. Epub 2020 Apr 27. PMID: 32342594. |
| 4 | Phloroglucinol | 0.71 | C6H6O3 | [M+H]+ | 127.03881 | -1.264179009 | 99.04401、69.03347 | Jinyinhua | Phenolic Acids | / | Zhang X, Yu X, Sun X, Meng X, Fan J, Zhang F, Zhang Y. Comparative study on chemical constituents of different medicinal parts of Lonicera japonica Thunb. Based on LC-MS combined with multivariate statistical analysis. Heliyon. 2024 May 29;10(12):e31722. doi: 10.1016/j.heliyon.2024.e31722. PMID: 38975169; PMCID: PMC11225679. |
| 5 | 5-Hydroxymethylfurfural | 0.72 | C6H6O3 | [M+H]+ | 127.0389 | -0.555734982 | 109.02831、81.03339 | Chishao | Others | / | Fu M,Sang X,Cheng H.Total glucosides of peony induce fibroblast-like synovial apoptosis, and ameliorate cartilage injury via blocking the NF-κB/STAT3 pathway.Ann Transl Med.2022 Jan;10(2):51. |
| 6 | L-Leucine | 0.73 | C6H13NO2 | [M+H]+ | 132.10164 | -2.007541069 | 86.09637、69.06972 | Jinyinhua、Bohe | Amino Acids | / | Chen Qiulin, Yao Cheng. Determination of Amino Acids in the Chinese Medicine Mentha Herb [J]. Journal of Nanjing Institute of Physical Education (Natural Science Edition), 2003, (04): 60-61+56. |
|  |  |  | C6H13NO2 | [M+H]+ | 132.10164 | -2.007541069 | 86.09637、69.06972 | Jinyinhua、Bohe | Amino Acids | / | Li Panlin, Li Chuyuan, Liu Menghua, et al. Comparison of Chemical Constituents between Honeysuckle and Lonicerae Flos Based on UFLC-Triple-Q-TOF-MS/MS Technology [J]. Central South Pharmacy, 2016, 14(04): 363-369. |
| 7 | Quinic acid | 0.73 | C7H12O6 | [M-H]- | 191.05594 | 4.844154457 | 127.04015、93.03468、87.00880、85.02958、59.01382 | Jinyinhua、Yuxingcao、Aiye | Organic Acids | / | Han B,Xin Z,Ma S,et al.Comprehensive characterization and identification of antioxidants in Folium Artemisiae Argyi using high-resolution tandem mass spectrometry.J Chromatogr B Analyt Technol Biomed Life Sci.2017 Sep 15;1063:84-92. |
|  |  |  | C7H12O6 | [M-H]- | 191.05594 | 4.844154457 | 127.04015、93.03468、87.00880、85.02958、59.01382 | Jinyinhua、Yuxingcao、Aiye | Organic Acids | / | Zhang Y,Huang X,Zhao F,et al.Study on the Chemical Markers of Caulis Lonicerae Japonicae for Quality Control By Hplc-qtof/ms/ms and Chromatographic Fingerprints Combined with Chemometrics Methods. Analytical Methods, 00, 2064-2076. http://dx.doi.org/10.1039/C4AY02744B. 10.1039/C4AY02744B. |
|  |  |  | C7H12O6 | [M-H]- | 191.05594 | 4.844154457 | 127.04015、93.03468、87.00880、85.02958、59.01382 | Jinyinhua、Yuxingcao、Aiye | Organic Acids | / | Mei Qianggen, Zhang Lu, Ma Tianxin, et al. Analysis of Antioxidant, Anti-diabetic Activities and Chemical Composition of the Extracted Fractions from Houttuynia cordata Aqueous Extract [J]. Food and Fermentation Industries, 2023, 49(11): 70-78. DOI:10.13995/j.cnki.11-1802/ts.030543. |
| 8 | Citric acid | 0.960 | C6H8O7 | [M-H]- | 191.01974 | 5.816186651 | 111.00881、87.00883、85.02958 | Yuxingcao | Organic Acids | / | Ju L, Zhang J, Wang F, Zhu D, Pei T, He Z, Han Z, Wang M, Ma Y, Xiao W. Chemical profiling of Houttuynia cordata Thunb. by UPLC-Q-TOF-MS and analysis of its antioxidant activity in C2C12 cells. J Pharm Biomed Anal. 2021 Sep 10;204:114271. doi: 10.1016/j.jpba.2021.114271. Epub 2021 Jul 16. PMID: 34325249. |
| 9 | Valine | 0.74 | C5H11NO2 | [M+H]+ | 118.08612 | -1.144925798 | 100.07568、72.08076 | Bohe | Amino Acids | / | Chen Qiulin, Yao Cheng. Determination of Amino Acids in the Chinese Medicine Mentha Herb [J]. Journal of Nanjing Institute of Physical Education (Natural Science Edition), 2003, (04): 60-61+56. |
| 10 | （+）-Malic acid | 0.74 | C4H6O5 | [M-H]- | 133.01416 | 7.595489636 | 133.01424、115.00365、71.01388 | Jinyinhua、Yuxingcao | Organic Acids | / | Zhang Y,Huang X,Zhao F,et al.Study on the Chemical Markers of Caulis Lonicerae Japonicae for Quality Control By Hplc-qtof/ms/ms and Chromatographic Fingerprints Combined with Chemometrics Methods. Analytical Methods, 00, 2064-2076. http://dx.doi.org/10.1039/C4AY02744B. 10.1039/C4AY02744B. |
|  |  |  | C4H6O5 | [M-H]- | 133.01416 | 7.595489636 | 133.01424、115.00365、71.01388 | Jinyinhua、Yuxingcao | Organic Acids | / | Ju L, Zhang J, Wang F, Zhu D, Pei T, He Z, Han Z, Wang M, Ma Y, Xiao W. Chemical profiling of Houttuynia cordata Thunb. by UPLC-Q-TOF-MS and analysis of its antioxidant activity in C2C12 cells. J Pharm Biomed Anal. 2021 Sep 10;204:114271. doi: 10.1016/j.jpba.2021.114271. Epub 2021 Jul 16. PMID: 34325249. |
| 11 | Sucrose* | 0.74 | C12H22O11 | [M-H]- | 341.1087 | 2.527646405 | 119.03503、、101.02448、89.02451、71.01392 | Jinyinhua | Others | Verified by reference standards. | Gong Xingcheng, Liu Wenjing, Cao Libo, et al. Rapid Qualitative Analysis of Chemical Constituents in Honeysuckle by DI-MS/MSALL [J]. China Journal of Chinese Materia Medica, 2021, 46(09): 2220-2228. DOI:10.19540/j.cnki.cjcmm.20210220.302. |
| 12 | 1-O-caffeoylquinic acid | 2.8 | C16H18O9 | [M-H]- | 353.08759 | 2.496553902 | 191.05618、179.05618 | Jinyinhua | Phenolic Acids | / | Cai Z, Liao H, Wang C, Chen J, Tan M, Mei Y, Wei L, Chen H, Yang R, Liu X. A comprehensive study of the aerial parts of Lonicera japonica Thunb. based on metabolite profiling coupled with PLS-DA. Phytochem Anal. 2020 Nov;31(6):786-800. doi: 10.1002/pca.2943. Epub 2020 Apr 27. PMID: 32342594. |
| 13 | galloylsucrose | 0.74 | C19H26O15 | [M-H]- | 493.11975 | 1.934016726 | 331.06677、313.05658、271.04590、169.01425、151.00415 | Chishao | Tannins | / | Tanaka T, Kataoka M, Tsuboi N, Kouno I. New monoterpene glycoside esters and phenolic constituents of Paeoniae radix, and increase of water solubility of proanthocyanidins in the presence of paeoniflorin. Chem Pharm Bull (Tokyo). 2000 Feb;48(2):201-7. doi: 10.1248/cpb.48.201. PMID: 10705504. |
| 14 | 1-O-galloyl-β-D-glucose | 0.75 | C13H16O10 | [M-H]- | 331.06674 | 2.316758781 | 271.04602、241.03535、211.02483、169.01427、125.02451 | Chishao | Phenolic Acids | / | Erşan S, Güçlü Üstündağ Ö, Carle R, Schweiggert RM. Identification of Phenolic Compounds in Red and Green Pistachio (Pistacia vera L.) Hulls (Exo- and Mesocarp) by HPLC-DAD-ESI-(HR)-MS(n). J Agric Food Chem. 2016 Jul 6;64(26):5334-44. doi: 10.1021/acs.jafc.6b01745. Epub 2016 Jun 23. PMID: 27292533. |
| 15 | 6"-O-galloylsucrose | 0.76 | C19H26O15 | [M-H]- | 493.11957 | 1.568993123 | 313.05658、169.01425、125.02438、123.00893 | Chishao | Tannins | / | Xiong P,Qin S,Li K,et al.Identification of the Tannins in Traditional Chinese Medicine Paeoniae Radix Alba By UHPLC-Q-Exactive Orbitrap Ms. Arabian Journal of Chemistry,14,103398. |
| 16 | galloylglucose | 0.79 | C13H16O10 | [M-H]- | 311.09799 | -60314.21115 | 271.14602、241.03535、169.01427、125.02451 | Chishao | Flavonoids | / | Xiong P,Qin S,Li K,et al.Identification of the Tannins in Traditional Chinese Medicine Paeoniae Radix Alba By UHPLC-Q-Exactive Orbitrap Ms. Arabian Journal of Chemistry,14,103398. |
| 17 | Paeonilactone B | 0.81 | C10H12O4 | [M-H]- | 195.05099 | -72.77208374 | 177.04062、87.08898、59.01395 | Chishao | Monoterpenes | / | Jin Zhongxian, Yu Jiahe, Liu Jinfeng, et al. Chemical Composition Analysis of Paeoniae Radix Rubra Based on UHPLC-Q-Exactive-Orbitrap-MS and Its Anti-psoriatic Activity Study [J]. Central South Pharmacy, 2023, 21(04): 894-902. |
| 18 | Threonine | 0.97 | C4H9NO3 | [M+H]+ | 120.06573 | 1.751543662 | 93.06973、74.06010 | Jinyinhua | Amino Acids | / | Zhang X, Yu X, Sun X, Meng X, Fan J, Zhang F, Zhang Y. Comparative study on chemical constituents of different medicinal parts of Lonicera japonica Thunb. Based on LC-MS combined with multivariate statistical analysis. Heliyon. 2024 May 29;10(12):e31722. doi: 10.1016/j.heliyon.2024.e31722. PMID: 38975169; PMCID: PMC11225679. |
| 19 | 8-Debenzoylpaeoniflorin | 0.97 | C16H24O10 | [M+HCOO]- | 421.13516 | 2.629803962 | 375.12946、345.11890、195.06622 | Chishao | Monoterpenes | / | He Xiao, Wu Huimin, Xiong Lele, et al. Study on Monoterpene Components in Paeoniae Radix Rubra Based on UPLC-Q-TOF-MS Technology [J]. China Journal of Chinese Materia Medica, 2023, 48(04): 1005-1013. DOI:10.19540/j.cnki.cjcmm.20220728.201. |
| 20 | Uridine* | 0.99 | C9H12N2O6 | [M-H]- | 243.06233 | 4.803317766 | 200.05577、152.03531、140.03596、110.02486 | Jinyinhua、Yuxingcao | Others | Verified by reference standards. | Shi Yuwen. Pharmaceutical Research of Yinqiao Mabo Granules [D]. Changchun University of Chinese Medicine, 2024. DOI:10.26980/d.cnki.gcczc.2024.000774. |
|  |  |  | C9H12N2O6 | [M-H]- | 243.06233 | 4.803317766 | 200.05577、152.03531、140.03596、110.02486 | Jinyinhua、Yuxingcao | Others | Verified by reference standards. | Ju L, Zhang J, Wang F, Zhu D, Pei T, He Z, Han Z, Wang M, Ma Y, Xiao W. Chemical profiling of Houttuynia cordata Thunb. by UPLC-Q-TOF-MS and analysis of its antioxidant activity in C2C12 cells. J Pharm Biomed Anal. 2021 Sep 10;204:114271. doi: 10.1016/j.jpba.2021.114271. Epub 2021 Jul 16. PMID: 34325249. |
| 21 | Isoleucine | 1.12 | C6H13NO2 | [M+H]+ | 132.10181 | -0.720655768 | 86.09634、69.09682 | Jinyinhua、Bohe | Amino Acids | / | Chen Qiulin, Yao Cheng. Determination of Amino Acids in the Chinese Medicine Mentha Herb [J]. Journal of Nanjing Institute of Physical Education (Natural Science Edition), 2003, (04): 60-61+56. |
|  |  |  | C6H13NO2 | [M+H]+ | 132.10181 | -0.720655768 | 86.09634、69.09682 | Jinyinhua、Bohe | Amino Acids | / | Cai Z, Liao H, Wang C, Chen J, Tan M, Mei Y, Wei L, Chen H, Yang R, Liu X. A comprehensive study of the aerial parts of Lonicera japonica Thunb. based on metabolite profiling coupled with PLS-DA. Phytochem Anal. 2020 Nov;31(6):786-800. doi: 10.1002/pca.2943. Epub 2020 Apr 27. PMID: 32342594. |
| 22 | [Guanine](https://www.chemsrc.com/en/cas/73-40-5_894557.html) | 1.17 | C5H5N5O | [M+H]+ | 152.05655 | -0.897033884 | 135.03110、110.97517 | Jinyinhua | Alkaloids | / | Zhang X, Yu X, Sun X, Meng X, Fan J, Zhang F, Zhang Y. Comparative study on chemical constituents of different medicinal parts of Lonicera japonica Thunb. Based on LC-MS combined with multivariate statistical analysis. Heliyon. 2024 May 29;10(12):e31722. doi: 10.1016/j.heliyon.2024.e31722. PMID: 38975169; PMCID: PMC11225679. |
| 23 | guanosine | 1.19 | C10H13N5O5 | [M-H]- | 282.08441 | 3.952733181 | 150.4221 | Yuxingcao | Others | / | Ju L, Zhang J, Wang F, Zhu D, Pei T, He Z, Han Z, Wang M, Ma Y, Xiao W. Chemical profiling of Houttuynia cordata Thunb. by UPLC-Q-TOF-MS and analysis of its antioxidant activity in C2C12 cells. J Pharm Biomed Anal. 2021 Sep 10;204:114271. doi: 10.1016/j.jpba.2021.114271. Epub 2021 Jul 16. PMID: 34325249. |
| 24 | Gallic acid* | 1.25 | C7H6O5 | [M-H]- | 169.01418 | 6.095975383 | 126.02795、125.02444、97.02960、81.03466、69.03469 | Yuxingcao、Chishao | Phenolic Acids | Verified by reference standards. | Xiong P,Qin S,Li K,et al.Identification of the Tannins in Traditional Chinese Medicine Paeoniae Radix Alba By UHPLC-Q-Exactive Orbitrap Ms. Arabian Journal of Chemistry,14,103398. |
|  |  |  | C7H6O5 | [M-H]- | 169.01418 | 6.095975383 | 126.02795、125.02444、97.02960、81.03466、69.03469 | Yuxingcao、Chishao | Phenolic Acids | Verified by reference standards. | Ju L, Zhang J, Wang F, Zhu D, Pei T, He Z, Han Z, Wang M, Ma Y, Xiao W. Chemical profiling of Houttuynia cordata Thunb. by UPLC-Q-TOF-MS and analysis of its antioxidant activity in C2C12 cells. J Pharm Biomed Anal. 2021 Sep 10;204:114271. doi: 10.1016/j.jpba.2021.114271. Epub 2021 Jul 16. PMID: 34325249. |
| 25 | pyrogallol | 1.26 | C6H6O3 | [M-H]- | 125.02439 | 8.554404056 | 81.03466 | Chishao | Phenolic Acids | / | Jin Zhongxian, Yu Jiahe, Liu Jinfeng, et al. Chemical Composition Analysis of Paeoniae Radix Rubra Based on UHPLC-Q-Exactive-Orbitrap-MS and Its Anti-psoriatic Activity Study [J]. Central South Pharmacy, 2023, 21(04): 894-902. |
| 26 | L-Phenylalanine | 1.74 | C9H11NO2 | [M+H]+ | 166.08611 | -0.874244529 | 149.05956、120.08070 | Jinyinhua | Amino Acids | / | Cai Z, Liao H, Wang C, Chen J, Tan M, Mei Y, Wei L, Chen H, Yang R, Liu X. A comprehensive study of the aerial parts of Lonicera japonica Thunb. based on metabolite profiling coupled with PLS-DA. Phytochem Anal. 2020 Nov;31(6):786-800. doi: 10.1002/pca.2943. Epub 2020 Apr 27. PMID: 32342594. |
| 27 | 6-O-galloylsucrose | 1.79 | C19H26O15 | [M-H]- | 493.11993 | 2.29904033 | 331.06711、313.05661、283.04602、169.01419 | Chishao | Tannins | / | Xiong P,Qin S,Li K,et al.Identification of the Tannins in Traditional Chinese Medicine Paeoniae Radix Alba By UHPLC-Q-Exactive Orbitrap Ms. Arabian Journal of Chemistry,14,103398. |
| 28 | 1-O-galloylsucrose | 1.97 | C19H26O15 | [M-H]- | 493.11981 | 2.055691261 | 313.05667、169.01427、151.00383、125.02457 | Chishao | Tannins | / | Xiong P,Qin S,Li K,et al.Identification of the Tannins in Traditional Chinese Medicine Paeoniae Radix Alba By UHPLC-Q-Exactive Orbitrap Ms. Arabian Journal of Chemistry,14,103398. |
| 29 | Danshensu | 2.04 | C9H10O5 | [M-H]- | 197.04553 | 5.481504303 | 197.04556、179.03494、123.04533、107.90759 | Bohe | Phenolic Acids | / | Shan QY, Cao G, Cai H, Cong XD, Cai BC. Novel software-based method to classify structurally similar compounds combined with high performance liquid chromatography-quadrupole time of flight mass spectrometry to identify complex components of herbal medicines. J Chromatogr A. 2012 Nov 16;1264:13-21. doi: 10.1016/j.chroma.2012.09.045. Epub 2012 Sep 23. PMID: 23062874. |
| 30 | Protocatechuic acid* | 2.220 | C7H6O4 | [M-H]- | 153.01929 | 6.893949596 | 109.02953、81.03474 | Jinyinhua、Yuxingcao、Chishao、Bohe | Phenolic Acids | Verified by reference standards. | Liu R,Wang Y,Liang C,et al.Morphology and mass spectrometry-based chemical profiling of peltate glandular trichomes on Mentha haplocalyx Briq leaves.Food Res Int.2023 Feb;164:112323. |
|  |  |  | C7H6O4 | [M-H]- | 153.01929 | 6.893949596 | 109.02953、81.03474 | Jinyinhua、Yuxingcao、Chishao、Bohe | Phenolic Acids | Verified by reference standards. | Liu Jie, Chen Lin, Fan Cairong, et al. Qualitative and Quantitative Study on Main Components of Paeoniae Radix Alba and Paeoniae Radix Rubra Based on HPLC-DAD-Q-TOF-MS/MS [J]. China Journal of Chinese Materia Medica, 2015, 40(09): 1762-1770. |
|  |  |  | C7H6O4 | [M-H]- | 153.01929 | 6.893949596 | 109.02953、81.03474 | Jinyinhua、Yuxingcao、Chishao、Bohe | Phenolic Acids | Verified by reference standards. | Gong Xingcheng, Liu Wenjing, Cao Libo, et al. Rapid Qualitative Analysis of Chemical Constituents in Honeysuckle by DI-MS/MSALL [J]. China Journal of Chinese Materia Medica, 2021, 46(09): 2220-2228. DOI:10.19540/j.cnki.cjcmm.20210220.302. |
|  |  |  | C7H6O4 | [M-H]- | 153.01929 | 6.893949596 | 109.02953、81.03474 | Jinyinhua、Yuxingcao、Chishao、Bohe | Phenolic Acids | Verified by reference standards. | Mei Qianggen, Zhang Lu, Ma Tianxin, et al. Analysis of Antioxidant, Anti-diabetic Activities and Chemical Composition of the Extracted Fractions from Houttuynia cordata Aqueous Extract [J]. Food and Fermentation Industries, 2023, 49(11): 70-78. DOI:10.13995/j.cnki.11-1802/ts.030543. |
| 31 | Scandoside | 2.24 | [C16H22O11](https://pubchem.ncbi.nlm.nih.gov/#query=C16H22O11) | [M-H]- | 389.10876 | 2.37003707 | 227.05673、183.06622、165.05545、121.06598 | Jinyinhua | Iridoid Glycosides | / | Zhang Y., Huang X., Zhao F., Tang Y., & Yin L. (2015). Study on the Chemical Markers of Caulis Lonicerae Japonicae for Quality Control By Hplc-qtof/ms/ms and Chromatographic Fingerprints Combined with Chemometrics Methods. Analytical Methods, 00, 2064-2076. http://dx.doi.org/10.1039/C4AY02744B. 10.1039/C4AY02744B. |
| 32 | Neochlorogenic acid* | 2.77 | C16H18O9 | [M+H]+ | 355.1021 | -0.728240728 | 163.03902 | Jinyinhua、Yuxingcao、Aiye | Phenolic Acids | Verified by reference standards. | Lan Xiaoyan, Zhu Longbo, Huang Xianzhang, et al. Identification and Content Determination of Main Chemical Components in Artemisiae Argyi Folium [J]. Chinese Traditional and Herbal Drugs, 2021, 52(24): 7630-7637. |
|  |  |  | C16H18O9 | [M+H]+ | 355.1021 | -0.728240728 | 163.03902 | Jinyinhua、Yuxingcao、Aiye | Phenolic Acids | Verified by reference standards. | Zhang Y,Huang X,Zhao F,et al.Study on the Chemical Markers of Caulis Lonicerae Japonicae for Quality Control By Hplc-qtof/ms/ms and Chromatographic Fingerprints Combined with Chemometrics Methods. Analytical Methods, 00, 2064-2076. http://dx.doi.org/10.1039/C4AY02744B. 10.1039/C4AY02744B. |
|  |  |  | C16H18O9 | [M+H]+ | 355.1021 | -0.728240728 | 163.03902 | Jinyinhua、Yuxingcao、Aiye | Phenolic Acids | Verified by reference standards. | Ahn J,Kim J.Chemical constituents from Houttuynia cordata[J].Planta Med,2016,81(S1):S1-S381. |
| 33 | 5-O-caffeoylquinic acid | 2.83 | C16H18O9 | [M-H]- | 353.08752 | 2.298302316 | 191.05612、179.03499、135.0452 | Jinyinhua | Phenolic Acids | / | Zhang Y,Huang X,Zhao F,et al.Study on the Chemical Markers of Caulis Lonicerae Japonicae for Quality Control By Hplc-qtof/ms/ms and Chromatographic Fingerprints Combined with Chemometrics Methods. Analytical Methods, 00, 2064-2076. http://dx.doi.org/10.1039/C4AY02744B. 10.1039/C4AY02744B. |
| 34 | Protocatechualdehyde | 3.06 | C7H6O3 | [M-H]- | 137.0244 | 7.878220992 | 109.02946 | Yuxingcao、Chishao、Bohe | Phenolic Acids | / | Wu Jiani, Xia Shuxue, Li Xuemei, et al. Fingerprint and Component Analysis of Mint Standard Decoction [J]. Chinese Journal of Experimental Traditional Medical Formulae, 2019, 25(16): 128-134. DOI:10.13422/j.cnki.syfjx.20191612. |
|  |  |  | C7H6O3 | [M-H]- | 137.0244 | 7.878220992 | 109.02946 | Yuxingcao、Chishao、Bohe | Phenolic Acids | / | Liu Jie, Chen Lin, Fan Cairong, et al. Qualitative and Quantitative Study on Main Components of Paeoniae Radix Alba and Paeoniae Radix Rubra Based on HPLC-DAD-Q-TOF-MS/MS [J]. China Journal of Chinese Materia Medica, 2015, 40(09): 1762-1770. |
|  |  |  | C7H6O3 | [M-H]- | 137.0244 | 7.878220992 | 109.02946 | Yuxingcao、Chishao、Bohe | Phenolic Acids | / | Mei Qianggen, Zhang Lu, Ma Tianxin, et al. Analysis of Antioxidant, Anti-diabetic Activities and Chemical Composition of the Extracted Fractions from Houttuynia cordata Aqueous Extract [J]. Food and Fermentation Industries, 2023, 49(11): 70-78. DOI:10.13995/j.cnki.11-1802/ts.030543. |
| 35 | Salicylic acid | 3.12 | C7H6O3 | [M-H]- | 137.02437 | 7.659280159 | 109.02983、93.03463 | Chishao、Aiye | Phenolic Acids | / | ZHANG LB,LV JL,CHEN HL,et al.Chemical constituentsfrom Artemisia argyi and their chemotaxonomic significance[J].Biochem Syst Ecol,2013,50:455. |
|  |  |  | C7H6O3 | [M-H]- | 137.02437 | 7.659280159 | 109.02983、93.03463 | Chishao、Aiye | Phenolic Acids | / | Xu C, Wang X, Han J, Gu Z, Guo Q. LMD and LC-MS-based chemical constituents and pharmacological effects assessment for two different processing methods of the root of Paeonia lactiflora Pall. J Pharm Biomed Anal. 2024 Aug 1;245:116184. doi: 10.1016/j.jpba.2024.116184. Epub 2024 Apr 27. PMID: 38692214. |
| 36 | 4-Hydroxybenzoic acid | 3.18 | C7H6O3 | [M-H]- | 137.02425 | 6.783516825 | 93.03457 | Jinyinhua、Yuxingcao、Chishao、Aiye | Phenolic Acids | / | Kim KO,Lee D,Hiep NT,et al.Protective Effect of Phenolic Compounds Isolated from Mugwort(Artemisia argyi)against Contrast-Induced Apoptosis in Kidney Epithelium Cell Line LLC-PK1.Molecules.2019 Jan 7;24(1):195. |
|  |  |  | C7H6O3 | [M-H]- | 137.02425 | 6.783516825 | 93.03457 | Jinyinhua、Yuxingcao、Chishao、Aiye | Phenolic Acids | / | Jin Zhongxian, Yu Jiahe, Liu Jinfeng, et al. Chemical Composition Analysis of Paeoniae Radix Rubra Based on UHPLC-Q-Exactive-Orbitrap-MS and Its Anti-psoriatic Activity Study [J]. Central South Pharmacy, 2023, 21(04): 894-902. |
|  |  |  | C7H6O3 | [M-H]- | 137.02425 | 6.783516825 | 93.03457 | Jinyinhua、Yuxingcao、Chishao、Aiye | Phenolic Acids | / | Zhang X, Yu X, Sun X, Meng X, Fan J, Zhang F, Zhang Y. Comparative study on chemical constituents of different medicinal parts of Lonicera japonica Thunb. Based on LC-MS combined with multivariate statistical analysis. Heliyon. 2024 May 29;10(12):e31722. doi: 10.1016/j.heliyon.2024.e31722. PMID: 38975169; PMCID: PMC11225679. |
|  |  |  | C7H6O3 | [M-H]- | 137.02425 | 6.783516825 | 93.03457 | Jinyinhua、Yuxingcao、Chishao、Aiye | Phenolic Acids | / | Ju L, Zhang J, Wang F, Zhu D, Pei T, He Z, Han Z, Wang M, Ma Y, Xiao W. Chemical profiling of Houttuynia cordata Thunb. by UPLC-Q-TOF-MS and analysis of its antioxidant activity in C2C12 cells. J Pharm Biomed Anal. 2021 Sep 10;204:114271. doi: 10.1016/j.jpba.2021.114271. Epub 2021 Jul 16. PMID: 34325249. |
| 37 | Secologanate | 3.49 | C16H22O10 | [M-H]- | 373.11383 | 2.43036342 | 211.06041、167.07130、149.06131、123.04526 | Jinyinhua | Iridoid Glycosides | / | Zhang Y,Huang X,Zhao F,et al.Study on the Chemical Markers of Caulis Lonicerae Japonicae for Quality Control By Hplc-qtof/ms/ms and Chromatographic Fingerprints Combined with Chemometrics Methods. Analytical Methods, 00, 2064-2076. http://dx.doi.org/10.1039/C4AY02744B. 10.1039/C4AY02744B. |
| 38 | Geniposidic acid* | 3.5 | C16H22O10 | [M-H]- | 373.11377 | 2.269554195 | 167.07137、149.06083、123.04524、89.02452 | Jinyinhua | Iridoid Glycosides | Verified by reference standards. | Cai Z, Liao H, Wang C, Chen J, Tan M, Mei Y, Wei L, Chen H, Yang R, Liu X. A comprehensive study of the aerial parts of Lonicera japonica Thunb. based on metabolite profiling coupled with PLS-DA. Phytochem Anal. 2020 Nov;31(6):786-800. doi: 10.1002/pca.2943. Epub 2020 Apr 27. PMID: 32342594. |
| 39 | Secologanic acid | 3.55 | C16H22O10 | [M-H]- | 373.11389 | 2.591172645 | 211.06134、167.07137、149.06082、123.04524 | Jinyinhua | Iridoid Glycosides | / | Cai Z, Liao H, Wang C, Chen J, Tan M, Mei Y, Wei L, Chen H, Yang R, Liu X. A comprehensive study of the aerial parts of Lonicera japonica Thunb. based on metabolite profiling coupled with PLS-DA. Phytochem Anal. 2020 Nov;31(6):786-800. doi: 10.1002/pca.2943. Epub 2020 Apr 27. PMID: 32342594. |
| 40 | methyl 4-acetoxy-3-hydroxybutanoate | 3.63 | C7H12O5 | [M-H]- | 175.06093 | 4.741800105 | 115.04015、85.06598 | Yuxingcao | Others | / | Ju L, Zhang J, Wang F, Zhu D, Pei T, He Z, Han Z, Wang M, Ma Y, Xiao W. Chemical profiling of Houttuynia cordata Thunb. by UPLC-Q-TOF-MS and analysis of its antioxidant activity in C2C12 cells. J Pharm Biomed Anal. 2021 Sep 10;204:114271. doi: 10.1016/j.jpba.2021.114271. Epub 2021 Jul 16. PMID: 34325249. |
| 41 | Mudanpioside F | 3.74 | C16H24O8 | [M+HCOO]- | 389.14514 | 2.355424917 | 343.13879、181.08540 | Chishao | Monoterpenes | / | Jin Zhongxian, Yu Jiahe, Liu Jinfeng, et al. Chemical Composition Analysis of Paeoniae Radix Rubra Based on UHPLC-Q-Exactive-Orbitrap-MS and Its Anti-psoriatic Activity Study [J]. Central South Pharmacy, 2023, 21(04): 894-902. |
| 42 | 6'-O-Galloyldebenzoylpaeoniflorin | 3.85 | C23H28O14 | [M-H]- | 527.14050 | 1.836705353 | 313.05618、271.04721、169.01465 | Chishao | Monoterpenes | / | He Xiao, Wu Huimin, Xiong Lele, et al. Study on Monoterpene Components in Paeoniae Radix Rubra Based on UPLC-Q-TOF-MS Technology [J]. China Journal of Chinese Materia Medica, 2023, 48(04): 1005-1013. DOI:10.19540/j.cnki.cjcmm.20220728.201. |
| 43 | Loganic acid* | 3.89 | C16H24O10 | [M-H]- | 375.12939 | 2.177120215 | 213.07689、169.08708、113.02455、69.03466 | Jinyinhua | Iridoid Glycosides | Verified by reference standards. | Gong Xingcheng, Liu Wenjing, Cao Libo, et al. Rapid Qualitative Analysis of Chemical Constituents in Honeysuckle by DI-MS/MSALL [J]. China Journal of Chinese Materia Medica, 2021, 46(09): 2220-2228. DOI:10.19540/j.cnki.cjcmm.20210220.302. |
| 44 | Chlorogenic acid* | 4.17 | C16H18O9 | [M-H]- | 353.08719 | 1.363687696 | 191.05606、179.03519、170.94159、161.02452、135.04543 | Jinyinhua、Yuxingcao、Chishao、Aiye、Bohe | Phenolic Acids | Verified by reference standards. | Lan Xiaoyan, Zhu Longbo, Huang Xianzhang, et al. Identification and Content Determination of Main Chemical Components in Artemisiae Argyi Folium [J]. Chinese Traditional and Herbal Drugs, 2021, 52(24): 7630-7637. |
|  |  |  | C16H18O9 | [M-H]- | 353.08719 | 1.363687696 | 191.05606、179.03519、170.94159、161.02452、135.04543 | Jinyinhua、Yuxingcao、Chishao、Aiye、Bohe | Phenolic Acids | Verified by reference standards. | Wu Jiani, Xia Shuxue, Li Xuemei, et al. Fingerprint and Component Analysis of Mint Standard Decoction [J]. Chinese Journal of Experimental Traditional Medical Formulae, 2019, 25(16): 128-134. DOI:10.13422/j.cnki.syfjx.20191612. |
|  |  |  | C16H18O9 | [M-H]- | 353.08719 | 1.363687696 | 191.05606、179.03519、170.94159、161.02452、135.04543 | Jinyinhua、Yuxingcao、Chishao、Aiye、Bohe | Phenolic Acids | Verified by reference standards. | Fu M,Sang X,Cheng H.Total glucosides of peony induce fibroblast-like synovial apoptosis, and ameliorate cartilage injury via blocking the NF-κB/STAT3 pathway.Ann Transl Med.2022 Jan;10(2):51. |
|  |  |  | C16H18O9 | [M-H]- | 353.08719 | 1.363687696 | 191.05606、179.03519、170.94159、161.02452、135.04543 | Jinyinhua、Yuxingcao、Chishao、Aiye、Bohe | Phenolic Acids | Verified by reference standards. | Ge L,Wan H,Tang S,et al.Novel caffeoylquinic acid derivatives from Lonicera japonica Thunb. flower buds exert pronounced anti-HBV activities. RSC Adv. 2018 Oct 15;8(62):35374-35385. |
|  |  |  | C16H18O9 | [M-H]- | 353.08719 | 1.363687696 | 191.05606、179.03519、170.94159、161.02452、135.04543 | Jinyinhua、Yuxingcao、Chishao、Aiye、Bohe | Phenolic Acids | Verified by reference standards. | Wu Xianjin, Li Shenghua, Li Aiming, et al. Study on the Chemical Constituents of Houttuynia cordata [J]. Journal of Chinese Medicinal Materials, 2008, (08): 1168-1170. |
| 45 | Morroniside | 4.198 | C17H26O11 | [M+HCOO]- | 451.14514 | 1.158830185 | 243.08752、155.03499、141.05573、101.02448 | Jinyinhua | Iridoid Glycosides | / | Cai Z, Liao H, Wang C, Chen J, Tan M, Mei Y, Wei L, Chen H, Yang R, Liu X. A comprehensive study of the aerial parts of Lonicera japonica Thunb. based on metabolite profiling coupled with PLS-DA. Phytochem Anal. 2020 Nov;31(6):786-800. doi: 10.1002/pca.2943. Epub 2020 Apr 27. PMID: 32342594. |
| 46 | Oxypaeoniflorin | 4.31 | C23H28O12 | [M-H]- | 495.15073 | 2.074928036 | 333.09830、195.06635、165.05579、137.02438 | Chishao | Monoterpenes | / | Liu Jie, Chen Lin, Fan Cairong, et al. Qualitative and Quantitative Study on Main Components of Paeoniae Radix Alba and Paeoniae Radix Rubra Based on HPLC-DAD-Q-TOF-MS/MS [J]. China Journal of Chinese Materia Medica, 2015, 40(09): 1762-1770. |
| 47 | Caffeic acid* | 4.44 | C9H8O4 | [M-H]- | 179.03491 | 5.724056085 | 150.95329、136.04854、135.04518、90.99812 | Jinyinhua | Phenolic Acids | Verified by reference standards. | Gong Xingcheng, Liu Wenjing, Cao Libo, et al. Rapid Qualitative Analysis of Chemical Constituents in Honeysuckle by DI-MS/MSALL [J]. China Journal of Chinese Materia Medica, 2021, 46(09): 2220-2228. DOI:10.19540/j.cnki.cjcmm.20210220.302. |
| 48 | 8-Epi loganic acid | 4.48 | [C16H24O10](https://pubchem.ncbi.nlm.nih.gov/#query=C16H24O10) | [M-H]- | 375.12930 | 1.937202473 | 169.08688、151.07634、59.01384 | Jinyinhua | Iridoid Glycosides | / | Cai Z, Liao H, Wang C, Chen J, Tan M, Mei Y, Wei L, Chen H, Yang R, Liu X. A comprehensive study of the aerial parts of Lonicera japonica Thunb. based on metabolite profiling coupled with PLS-DA. Phytochem Anal. 2020 Nov;31(6):786-800. doi: 10.1002/pca.2943. Epub 2020 Apr 27. PMID: 32342594. |
| 49 | Cryptochlorogenic acid* | 4.75 | C16H18O9 | [M-H]- | 353.0875 | 2.241659006 | 191.05620、179.03505、173.04561、155.03517、135.04527 | Yuxingcao、Bohe、Aiye | Phenolic Acids | Verified by reference standards. | Lan Xiaoyan, Zhu Longbo, Huang Xianzhang, et al. Identification and Content Determination of Main Chemical Components in Artemisiae Argyi Folium [J]. Chinese Traditional and Herbal Drugs, 2021, 52(24): 7630-7637. |
|  |  |  | C16H18O9 | [M-H]- | 353.0875 | 2.241659006 | 191.05620、179.03505、173.04561、155.03517、135.04527 | Yuxingcao、Bohe、Aiye | Phenolic Acids | Verified by reference standards. | Liu R,Wang Y,Liang C,et al.Morphology and mass spectrometry-based chemical profiling of peltate glandular trichomes on Mentha haplocalyx Briq leaves.Food Res Int.2023 Feb;164:112323. |
|  |  |  | C16H18O9 | [M-H]- | 353.0875 | 2.241659006 | 191.05620、179.03505、173.04561、155.03517、135.04527 | Yuxingcao、Bohe、Aiye | Phenolic Acids | Verified by reference standards. | Ahn J,Kim J.Chemical constituents from Houttuynia cordata[J].Planta Med,2016,81(S1):S1-S381. |
| 50 | secologanoside | 4.8 | C16H22O11 | [M-H]- | 389.10855 | 1.830340925 | 209.04498、183.06647、165.05577、121.06598 | Jinyinhua | Iridoid Glycosides | / | Ren MT, Chen J, Song Y, Sheng LS, Li P, Qi LW. Identification and quantification of 32 bioactive compounds in Lonicera species by high performance liquid chromatography coupled with time-of-flight mass spectrometry. J Pharm Biomed Anal. 2008 Dec 15;48(5):1351-60. doi: 10.1016/j.jpba.2008.09.037. Epub 2008 Sep 30. PMID: 18977626. |
| 51 | caffeic acid methyl ester | 5.06 | C10H10O4 | [M+H]+ | 195.06493 | -1.308793261 | 177.05443、151.03886、109.02831 | Jinyinhua | Phenolic Acids | / | Xiong Lewen, Zhang Longfei, Li Jia, et al. Extraction Optimization and Component Distribution of Phenolic Acids in Honeysuckle [J]. Journal of Chinese Medicinal Materials, 2022, 45(08): 1939-1945. DOI:10.13863/j.issn1001-4454.2022.08.029. |
| 52 | roseoside | 5.07 | C20H32O10 | [M-H]- | 431.07263 | -274.92098 | 193.05106、149.06082 | Yuxingcao | Others | / | Ju L, Zhang J, Wang F, Zhu D, Pei T, He Z, Han Z, Wang M, Ma Y, Xiao W. Chemical profiling of Houttuynia cordata Thunb. by UPLC-Q-TOF-MS and analysis of its antioxidant activity in C2C12 cells. J Pharm Biomed Anal. 2021 Sep 10;204:114271. doi: 10.1016/j.jpba.2021.114271. Epub 2021 Jul 16. PMID: 34325249. |
| 53 | [Trigallic Acid](https://www.chemsrc.com/en/cas/2131-66-0_1085531.html) | 4.19 | [C21H14O13](https://pubchem.ncbi.nlm.nih.gov/#query=C21H14O13) | [M-H]- | 473.03485 | -0.458316973 | 101.02463、71.01391、59.01393 | Chishao | Tannins | / | Xiong P,Qin S,Li K,et al.Identification of the Tannins in Traditional Chinese Medicine Paeoniae Radix Alba By UHPLC-Q-Exactive Orbitrap Ms. Arabian Journal of Chemistry,14,103398. |
| 54 | Swertiamarin | 5.09 | C16H22O10 | [M-H]- | 373.11362 | 1.867531133 | 193.05054、149.06064、119.03494、97.02953、89.02451 | Jinyinhua | Iridoid Glycosides | / | Jin Ying, Xiong Lewen, Pu Gaobin, et al. Comparison of Chemical Constituents between Lonicera fragrantissima Buds and Honeysuckle Based on Liquid Chromatography-Mass Spectrometry [J]. Chinese Traditional Patent Medicine, 2024, 46(03): 850-859. |
| 55 | Norisoboldine | 5.22 | C18H19NO4 | [M+H]+ | 314.13849 | -0.61947162 | 297.11157、282.08734、265.08594 | Yuxingcao | Alkaloids | / | Ju L, Zhang J, Wang F, Zhu D, Pei T, He Z, Han Z, Wang M, Ma Y, Xiao W. Chemical profiling of Houttuynia cordata Thunb. by UPLC-Q-TOF-MS and analysis of its antioxidant activity in C2C12 cells. J Pharm Biomed Anal. 2021 Sep 10;204:114271. doi: 10.1016/j.jpba.2021.114271. Epub 2021 Jul 16. PMID: 34325249. |
| 56 | Mudanpioside E | 5.29 | C24H30O13 | [M-H]- | 525.16132 | 2.004530932 | 495.14749、363.10745、195.06561、167.03494 | Chishao | Phenolic Glycosides | / | He Xiao, Wu Huimin, Xiong Lele, et al. Study on Monoterpene Components in Paeoniae Radix Rubra Based on UPLC-Q-TOF-MS Technology [J]. China Journal of Chinese Materia Medica, 2023, 48(04): 1005-1013. DOI:10.19540/j.cnki.cjcmm.20220728.201. |
| 57 | lonijaposide N | 5.65 | C26H33NO11 | [M+H]+ | 536.21204 | -1.113737272 | 304.11771、218.08148 | Jinyinhua | Monoterpenes | / | Zhang X, Yu X, Sun X, Meng X, Fan J, Zhang F, Zhang Y. Comparative study on chemical constituents of different medicinal parts of Lonicera japonica Thunb. Based on LC-MS combined with multivariate statistical analysis. Heliyon. 2024 May 29;10(12):e31722. doi: 10.1016/j.heliyon.2024.e31722. PMID: 38975169; PMCID: PMC11225679. |
| 58 | isoboldine | 5.98 | C19H21NO4 | [M+H]+ | 328.15414 | -0.593318385 | 297.11206、265.08594、237.09109 | Yuxingcao | Alkaloids | / | Wu Yingxue. Study on Alkaloid Components and Safety in Houttuynia cordata [D]. National Institutes for Food and Drug Control, 2022. DOI:10.27651/d.cnki.gzyss.2022.000006. |
| 59 | 7-O-ethyl sweroside | 6 | C18H26O10 | [M+HCOO]- | 447.15088 | 2.633122628 | 401.14331、269.10364 | Jinyinhua | Iridoid Glycosides | / | Zhang X, Yu X, Sun X, Meng X, Fan J, Zhang F, Zhang Y. Comparative study on chemical constituents of different medicinal parts of Lonicera japonica Thunb. Based on LC-MS combined with multivariate statistical analysis. Heliyon. 2024 May 29;10(12):e31722. doi: 10.1016/j.heliyon.2024.e31722. PMID: 38975169; PMCID: PMC11225679. |
| 60 | Sweroside | 6.38 | C16H22O9 | [M+HCOO]- | 403.12427 | 1.940100301 | 195.06708、151.07648、125.02451 | Jinyinhua | Iridoid Glycosides | / | Cai Z, Liao H, Wang C, Chen J, Tan M, Mei Y, Wei L, Chen H, Yang R, Liu X. A comprehensive study of the aerial parts of Lonicera japonica Thunb. based on metabolite profiling coupled with PLS-DA. Phytochem Anal. 2020 Nov;31(6):786-800. doi: 10.1002/pca.2943. Epub 2020 Apr 27. PMID: 32342594. |
| 61 | Albiflorin* | 6.62 | C23H28O11 | [M+H]+ | 481.16974 | -1.45083726 | 197.08069、179.07021、151.07533、133.06477、105.03343 | Chishao | Monoterpenes | Verified by reference standards. | Liu Jie, Chen Lin, Fan Cairong, et al. Qualitative and Quantitative Study on Main Components of Paeoniae Radix Alba and Paeoniae Radix Rubra Based on HPLC-DAD-Q-TOF-MS/MS [J]. China Journal of Chinese Materia Medica, 2015, 40(09): 1762-1770. |
| 62 | Loganin | 6.77 | C17H26O10 | [M+HCOO]- | 435.15076 | 2.429968339 | 227.09256、101.02422 | Jinyinhua | Iridoid Glycosides | / | Cai Z, Liao H, Wang C, Chen J, Tan M, Mei Y, Wei L, Chen H, Yang R, Liu X. A comprehensive study of the aerial parts of Lonicera japonica Thunb. based on metabolite profiling coupled with PLS-DA. Phytochem Anal. 2020 Nov;31(6):786-800. doi: 10.1002/pca.2943. Epub 2020 Apr 27. PMID: 32342594. |
| 63 | Isomaltopaeoniflorin | 6.95 | [C29H38O16](https://pubchem.ncbi.nlm.nih.gov/#query=C29H38O16) | [M+HCOO]- | 687.21448 | 2.021789193 | 165.05588、121.02953 | Chishao | Monoterpenes | / | Jin Zhongxian, Yu Jiahe, Liu Jinfeng, et al. Chemical Composition Analysis of Paeoniae Radix Rubra Based on UHPLC-Q-Exactive-Orbitrap-MS and Its Anti-psoriatic Activity Study [J]. Central South Pharmacy, 2023, 21(04): 894-902. |
| 64 | ethyl gallate | 6.97 | C9H10O5 | [M-H]- | 197.04549 | 5.278504421 | 125.95553、169.94527 | Chishao | Phenolic Acids | / | Liu Jie, Chen Lin, Fan Cairong, et al. Qualitative and Quantitative Study on Main Components of Paeoniae Radix Alba and Paeoniae Radix Rubra Based on HPLC-DAD-Q-TOF-MS/MS [J]. China Journal of Chinese Materia Medica, 2015, 40(09): 1762-1770. |
| 65 | Ferulaldehyde | 7.47 | C10H10O3 | [M+H]+ | 179.0699 | -2.070137039 | 161.06000、133.06468、105.06976 | Chishao | Others | / | Fan Weitong, Liao Caicen, Zhou Yan, et al. Chemical Constituents and Anti-inflammatory Activity of Paeoniae Radix Rubra [J]. Journal of Kunming University of Science and Technology (Natural Science Edition), 2022, 47(03): 117-122. DOI:10.16112/j.cnki.53-1223/n.2022.03.252. |
| 66 | paeonoside | 7.48 | C15H20O8 | [M-H]- | 327.10843 | 3.014300096 | 121.94346 | Chishao | Phenolic Glycosides | / | Liu Jie, Chen Lin, Fan Cairong, et al. Qualitative and Quantitative Study on Main Components of Paeoniae Radix Alba and Paeoniae Radix Rubra Based on HPLC-DAD-Q-TOF-MS/MS [J]. China Journal of Chinese Materia Medica, 2015, 40(09): 1762-1770. |
| 67 | Paeoniflorin* | 7.48 | C23H28O11 | [M+NH4]+ | 498.1967 | -0.576478797 | 179.07014、151.07524 | Chishao | Monoterpenes | Verified by reference standards. | Liu Jie, Chen Lin, Fan Cairong, et al. Qualitative and Quantitative Study on Main Components of Paeoniae Radix Alba and Paeoniae Radix Rubra Based on HPLC-DAD-Q-TOF-MS/MS [J]. China Journal of Chinese Materia Medica, 2015, 40(09): 1762-1770. |
| 68 | lonijaposide T | 7.97 | C28H35NO13 | [M+H]+ | 594.21722 | -1.508705263 | 432.16501、362.12320 | Jinyinhua | Monoterpenes | / | Zhang X, Yu X, Sun X, Meng X, Fan J, Zhang F, Zhang Y. Comparative study on chemical constituents of different medicinal parts of Lonicera japonica Thunb. Based on LC-MS combined with multivariate statistical analysis. Heliyon. 2024 May 29;10(12):e31722. doi: 10.1016/j.heliyon.2024.e31722. PMID: 38975169; PMCID: PMC11225679. |
| 69 | Benzoic acid | 8.07 | C7H6O2 | [M+H]+ | 123.04395 | -0.861480054 | 95.04919、82.01368、81.06955、67.05421 | Bohe | Others | / | Xu LL, Xu JJ, Zhong KR, Shang ZP, Wang F, Wang RF, Zhang L, Zhang JY, Liu B. Analysis of Non-Volatile Chemical Constituents of Menthae Haplocalycis Herba by Ultra-High Performance Liquid Chromatography-High Resolution Mass Spectrometry. Molecules. 2017 Oct 19;22(10):1756. doi: 10.3390/molecules22101756. PMID: 29048378; PMCID: PMC6151415. |
| 70 | 4-Hydroxycinnamic acid | 8.07 | [C9H8O3](https://pubchem.ncbi.nlm.nih.gov/#query=C9H8O3) | [M+H]+ | 165.05438 | -1.458305129 | 147.08025、91.05387 | Jinyinhua | Others | / | Xiong Lewen, Zhang Longfei, Li Jia, et al. Extraction Optimization and Component Distribution of Phenolic Acids in Honeysuckle [J]. Journal of Chinese Medicinal Materials, 2022, 45(08): 1939-1945. DOI:10.13863/j.issn1001-4454.2022.08.029. |
| 71 | Secoxyloganin | 8.08 | C17H24O11 | [M-H]- | 403.12418 | 1.716843649 | 371.09772、223.06122、165.05562、149.02438、121.02953 | Jinyinhua | Iridoid Glycosides | / | Cai Z, Liao H, Wang C, Chen J, Tan M, Mei Y, Wei L, Chen H, Yang R, Liu X. A comprehensive study of the aerial parts of Lonicera japonica Thunb. based on metabolite profiling coupled with PLS-DA. Phytochem Anal. 2020 Nov;31(6):786-800. doi: 10.1002/pca.2943. Epub 2020 Apr 27. PMID: 32342594. |
| 72 | Sarracenin | 8.402 | C11H14O5 | [M+H]+ | 227.0912 | -0.88070266 | 195.06523、177.05453、167.07002、151.03889 | Jinyinhua | Iridoid Glycosides | / | Shi Yuwen. Pharmaceutical Research of Yinqiao Mabo Granules [D]. Changchun University of Chinese Medicine, 2024. DOI:10.26980/d.cnki.gcczc.2024.000774. |
| 73 | 7-Ketologanin | 8.48 | [C17H24O10](https://pubchem.ncbi.nlm.nih.gov/#query=C17H24O10) | [M+HCOO]- | 433.13507 | 2.349157251 | 225.07680、123.04537、101.02458 | Jinyinhua | Iridoid Glycosides | / | Zhang Y., Huang X., Zhao F., Tang Y., & Yin L. (2015). Study on the Chemical Markers of Caulis Lonicerae Japonicae for Quality Control By Hplc-qtof/ms/ms and Chromatographic Fingerprints Combined with Chemometrics Methods. Analytical Methods, 00, 2064-2076. http://dx.doi.org/10.1039/C4AY02744B. 10.1039/C4AY02744B. |
| 74 | Schaftoside | 8.54 | C26H28O14 | [M-H]- | 563.13983 | 0.529531285 | 473.10812、443.09821、383.07773、353.06729 | Aiye | Flavonoids | / | Chang Y, Fan W, Shi H, Feng X, Zhang D, Wang L, Zheng Y, Guo L. Characterization of phenolics and discovery of 伪-glucosidase inhibitors in Artemisia argyi leaves based on ultra-performance liquid chromatography-tandem mass spectrometry and relevance analysis. J Pharm Biomed Anal. 2022 Oct 25;220:114982. doi: 10.1016/j.jpba.2022.114982. Epub 2022 Aug 4. PMID: 35944337. |
| 75 | Isoschaftoside | 8.62 | C26H28O14 | [M-H]- | 563.14063 | 1.950138355 | 473.10870、443.09811、383.07693、353.06631 | Aiye | Flavonoids | / | Lan Xiaoyan, Zhu Longbo, Huang Xianzhang, et al. Identification and Content Determination of Main Chemical Components in Artemisiae Argyi Folium [J]. Chinese Traditional and Herbal Drugs, 2021, 52(24): 7630-7637. |
| 76 | Isofraxidin* | 8.680 | C11H10O5 | [M+H]+ | 223.05988 | -0.985832967 | 208.03752、190.02638 | Aiye | Others | Verified by reference standards. | Kim KO,Lee D,Hiep NT,et al.Protective Effect of Phenolic Compounds Isolated from Mugwort(Artemisia argyi)against Contrast-Induced Apoptosis in Kidney Epithelium Cell Line LLC-PK1.Molecules.2019 Jan 7;24(1):195. |
| 77 | L-Phenylalaninosecologanin | 9.29 | C26H35NO11 | [M-H]- | 536.21393 | 2.410983834 | 356.15134、312.16049、272.09155、164.07166 | Jinyinhua | Iridoid Glycosides | / | Zhang Y., Huang X., Zhao F., Tang Y., & Yin L. (2015). Study on the Chemical Markers of Caulis Lonicerae Japonicae for Quality Control By Hplc-qtof/ms/ms and Chromatographic Fingerprints Combined with Chemometrics Methods. Analytical Methods, 00, 2064-2076. http://dx.doi.org/10.1039/C4AY02744B. 10.1039/C4AY02744B. |
| 78 | Lonijaposide B | 3.93 | C25H31NO12 | [M+H]+ | 538.19104 | -1.601287565 | 376.17520、358.16476 | Jinyinhua | Monoterpenes | / | Zhang X, Yu X, Sun X, Meng X, Fan J, Zhang F, Zhang Y. Comparative study on chemical constituents of different medicinal parts of Lonicera japonica Thunb. Based on LC-MS combined with multivariate statistical analysis. Heliyon. 2024 May 29;10(12):e31722. doi: 10.1016/j.heliyon.2024.e31722. PMID: 38975169; PMCID: PMC11225679. |
| 79 | Hyperoside* | 9.59 | C21H20O12 | [M-H]- | 463.0883 | 2.586122554 | 300.02759、178.99835、151.00337 | Jinyinhua、Yuxingcao | Flavonoids | Verified by reference standards. | Li XQ, Sun XH, Cai S, Ying XX, Li FM. Investigation on the chemical constituents and variation of the flower buds of Lonicera species by UPLC-ESI-MS/MS and principle component analysis. Yao Xue Xue Bao. 2009 Aug;44(8):895-904. PMID: 20055159. |
|  |  |  | C21H20O12 | [M-H]- | 463.0883 | 2.586122554 | 300.02759、178.99835、151.00337 | Jinyinhua、Yuxingcao | Flavonoids | Verified by reference standards. | Meng Jiang, Dong Xiaoping, Jiang Zhihong, et al. Study on Flavonoids from Fresh Houttuynia cordata [J]. China Journal of Chinese Materia Medica, 2006, (16): 1335-1337. |
| 80 | Rutin* | 9.71 | C27H30O16 | [M-H]- | 609.14636 | 2.214415247 | 343.04605、300.02753、178.99890、151.00369 | Jinyinhua、Aiye、Bohe | Flavonoids | Verified by reference standards. | Liu Qian, Fu Juan, Hu Junhua, et al. Chemical Component Analysis and Multi-index Quantitative Determination of Kechuanning Granules Based on HPLC-Q-TOF-MS/MS Technology [J]. Drug Evaluation Research, 2023, 46(05): 1012-1023. |
|  |  |  | C27H30O16 | [M-H]- | 609.14636 | 2.214415247 | 343.04605、300.02753、178.99890、151.00369 | Jinyinhua、Aiye、Bohe | Flavonoids | Verified by reference standards. | Lan Xiaoyan, Zhu Longbo, Huang Xianzhang, et al. Identification and Content Determination of Main Chemical Components in Artemisiae Argyi Folium [J]. Chinese Traditional and Herbal Drugs, 2021, 52(24): 7630-7637. |
|  |  |  | C27H30O16 | [M-H]- | 609.14636 | 2.214415247 | 343.04605、300.02753、178.99890、151.00369 | Jinyinhua、Aiye、Bohe | Flavonoids | Verified by reference standards. | Xu Jingjing. Study on Quality Control and Evaluation Methods for Mentha Herb Based on Spectrum-Effect Relationship Analysis of Antioxidant Activity [D]. Beijing University of Chinese Medicine, 2014. |
| 81 | Lithospermic acid | 9.88 | C27H22O12 | [M-H]- | 537.10388 | 2.099412068 | 313.07217、295.06113 | Bohe | Organic Acids | / | Liu R, Wang Y, Liang C, Zheng Z, Du X, Cui Z, Zhang Y, Liu H. Morphology and mass spectrometry-based chemical profiling of peltate glandular trichomes on Mentha haplocalyx Briq leaves. Food Res Int. 2023 Feb;164:112323. doi: 10.1016/j.foodres.2022.112323. Epub 2022 Dec 10. PMID: 36737916. |
| 82 | Isoquercitrin* | 9.9 | C21H20O12 | [M-H]- | 463.0882 | 2.370180457 | 300.02759、190.92828、146.93867 | Jinyinhua、Chishao、Aiye | Flavonoids | Verified by reference standards. | Lan Xiaoyan, Zhu Longbo, Huang Xianzhang, et al. Identification and Content Determination of Main Chemical Components in Artemisiae Argyi Folium [J]. Chinese Traditional and Herbal Drugs, 2021, 52(24): 7630-7637. |
|  |  |  | C21H20O12 | [M-H]- | 463.0882 | 2.370180457 | 300.02759、190.92828、146.93867 | Jinyinhua、Chishao、Aiye | Flavonoids | Verified by reference standards. | Fu M,Sang X,Cheng H.Total glucosides of peony induce fibroblast-like synovial apoptosis, and ameliorate cartilage injury via blocking the NF-κB/STAT3 pathway.Ann Transl Med.2022 Jan;10(2):51. |
|  |  |  | C21H20O12 | [M-H]- | 463.0882 | 2.370180457 | 300.02759、190.92828、146.93867 | Jinyinhua、Chishao、Aiye | Flavonoids | Verified by reference standards. | Zhang X, Yu X, Sun X, Meng X, Fan J, Zhang F, Zhang Y. Comparative study on chemical constituents of different medicinal parts of Lonicera japonica Thunb. Based on LC-MS combined with multivariate statistical analysis. Heliyon. 2024 May 29;10(12):e31722. doi: 10.1016/j.heliyon.2024.e31722. PMID: 38975169; PMCID: PMC11225679. |
| 83 | Astragalin | 10.23 | C21H20O11 | [M-H]- | 447.09329 | 2.465263384 | 285.04041、284.03287、174.95592 | Jinyinhua | Flavonoids | / | Cai Z, Liao H, Wang C, Chen J, Tan M, Mei Y, Wei L, Chen H, Yang R, Liu X. A comprehensive study of the aerial parts of Lonicera japonica Thunb. based on metabolite profiling coupled with PLS-DA. Phytochem Anal. 2020 Nov;31(6):786-800. doi: 10.1002/pca.2943. Epub 2020 Apr 27. PMID: 32342594. |
| 84 | Cynaroside* | 10.25 | C21H20O11 | [M+H]+ | 449.1023 | -12.33066879 | 287.05481、90.97650 | Jinyinhua | Others | Verified by reference standards. | Liu Shupeng, Huang Houyu, Wang Xiaoxue, et al. Revealing the Differences in Chemical Constituents and Anti-aging Mechanisms between Honeysuckle and Lonicerae Folium Based on UHPLC-QE-Orbitrap-MS Technology and Network Pharmacology [J]. Food Science, 2024, 45(20): 1-11. |
| 85 | galloylpaeoniflorin | 10.28 | C30H32O15 | [M-H]- | 631.16711 | 2.160288336 | 613.15295、509.13089、491.11557、271.04535 | Chishao | Monoterpenes | / | He Xiao, Wu Huimin, Xiong Lele, et al. Study on Monoterpene Components in Paeoniae Radix Rubra Based on UPLC-Q-TOF-MS Technology [J]. China Journal of Chinese Materia Medica, 2023, 48(04): 1005-1013. DOI:10.19540/j.cnki.cjcmm.20220728.201. |
| 86 | 5-Carboxystrictosidine | 10.56 | C28H34N2O11 | [M+H]+ | 575.22327 | -0.462950459 | 413.16980、396.14349、188.07045 | Jinyinhua | Iridoid Glycosides | / | Santos CLG, Angolini CFF, Neves KOG, Costa EV, de Souza ADL, Pinheiro MLB, Koolen HHF, da Silva FMA. Molecular networking-based dereplication of strictosidine-derived monoterpene indole alkaloids from the curare ingredient Strychnos peckii. Rapid Commun Mass Spectrom. 2020 Sep;34 Suppl 3:e8683. doi: 10.1002/rcm.8683. Epub 2020 Feb 18. PMID: 31783430. |
| 87 | Lonicerin | 10.64 | C27H30O15 | [M-H]- | 593.15149 | 2.349321037 | 447.09381、285.04050、284.03287 | Jinyinhua | Flavonoids | / | Cai Z, Liao H, Wang C, Chen J, Tan M, Mei Y, Wei L, Chen H, Yang R, Liu X. A comprehensive study of the aerial parts of Lonicera japonica Thunb. based on metabolite profiling coupled with PLS-DA. Phytochem Anal. 2020 Nov;31(6):786-800. doi: 10.1002/pca.2943. Epub 2020 Apr 27. PMID: 32342594. |
| 88 | Kaempferol-3-O-rutinoside | 10.93 | C27H30O15 | [M-H]- | 593.15137 | 2.147011368 | 285.04037、255.03078 | Jinyinhua、Aiye | Flavonoids | / | Kim SM,Lee SJ,Venkatarame Gowda Saralamma V,et al.Polyphenol mixture of a native Korean variety of Artemisia argyi H.(Seomae mugwort)and its anti‑inflammatory effects.Int J Mol Med.2019 Nov;44(5):1741-1752. |
|  |  |  | C27H30O15 | [M-H]- | 593.15137 | 2.147011368 | 285.04037、255.03078 | Jinyinhua、Aiye | Flavonoids | / | Cai Z, Liao H, Wang C, Chen J, Tan M, Mei Y, Wei L, Chen H, Yang R, Liu X. A comprehensive study of the aerial parts of Lonicera japonica Thunb. based on metabolite profiling coupled with PLS-DA. Phytochem Anal. 2020 Nov;31(6):786-800. doi: 10.1002/pca.2943. Epub 2020 Apr 27. PMID: 32342594. |
| 89 | Datiscin | 10.95 | C27H30O15 | [M-H]- | 593.15137 | 2.147011368 | 327.05267、285.04037 | Aiye | Flavonoids | / | Chang Y, Fan W, Shi H, Feng X, Zhang D, Wang L, Zheng Y, Guo L. Characterization of phenolics and discovery of 伪-glucosidase inhibitors in Artemisia argyi leaves based on ultra-performance liquid chromatography-tandem mass spectrometry and relevance analysis. J Pharm Biomed Anal. 2022 Oct 25;220:114982. doi: 10.1016/j.jpba.2022.114982. Epub 2022 Aug 4. PMID: 35944337. |
| 90 | [1,5-Dicaffeoylquinic acid](https://www.chemsrc.com/en/cas/30964-13-7_314962.html) | 11.13 | C25H24O12 | [M-H]- | 515.11938 | 1.897621974 | 353.08755、335.07755、191.05606、179.03493、161.03454、135.04510 | Jinyinhua、Aiye | Phenolic Acids | / | Lan Xiaoyan, Zhu Longbo, Huang Xianzhang, et al. Identification and Content Determination of Main Chemical Components in Artemisiae Argyi Folium [J]. Chinese Traditional and Herbal Drugs, 2021, 52(24): 7630-7637. |
|  |  |  | C25H24O12 | [M-H]- | 515.11938 | 1.897621974 | 353.08755、335.07755、191.05606、179.03493、161.03454、135.04510 | Jinyinhua、Aiye | Phenolic Acids | / | Jin Ying, Xiong Lewen, Pu Gaobin, et al. Comparison of Chemical Constituents between Lonicera fragrantissima Buds and Honeysuckle Based on Liquid Chromatography-Mass Spectrometry [J]. Chinese Traditional Patent Medicine, 2024, 46(03): 850-859. |
| 91 | Linarin* | 11.21 | C28H32O14 | [M+H]+ | 539.18524 | -91035.86079 | 447.12677、285.07541、85.02808 | Yuxingcao、Bohe | Flavonoids | Verified by reference standards. | Xu Jingjing. Study on Quality Control and Evaluation Methods for Mentha Herb Based on Spectrum-Effect Relationship Analysis of Antioxidant Activity [D]. Beijing University of Chinese Medicine, 2014. |
|  |  |  | C28H32O14 | [M+H]+ | 539.18524 | -91035.86079 | 447.12677、285.07541、85.02808 | Yuxingcao、Bohe | Flavonoids | Verified by reference standards. | Li D,Liu J,Han X,et al.Chemical constituents of the whole plants of Houttuynia cordata.[J]Chem Nat Compd,2017,53(2):365-367. |
| 92 | 3,5-Dicaffeoylquinic acid* | 11.22 | C25H24O12 | [M-H]- | 515.11957 | 2.266469212 | 353.08743、191.05605、179.03490、135.04517 | Jinyinhua、Aiye | Phenolic Acids | Verified by reference standards. | Lan Xiaoyan, Zhu Longbo, Huang Xianzhang, et al. Identification and Content Determination of Main Chemical Components in Artemisiae Argyi Folium [J]. Chinese Traditional and Herbal Drugs, 2021, 52(24): 7630-7637. |
|  |  |  | C25H24O12 | [M-H]- | 515.11957 | 2.266469212 | 353.08743、191.05605、179.03490、135.04517 | Jinyinhua、Aiye | Phenolic Acids | Verified by reference standards. | Jin Ying, Xiong Lewen, Pu Gaobin, et al. Comparison of Chemical Constituents between Lonicera fragrantissima Buds and Honeysuckle Based on Liquid Chromatography-Mass Spectrometry [J]. Chinese Traditional Patent Medicine, 2024, 46(03): 850-859. |
| 93 | Quercitrin* | 11.23 | C21H20O11 | [M-H]- | 447.09314 | 2.129762107 | 302.03778、301.03516、300.02753 | Yuxingcao | Flavonoids | Verified by reference standards. | Xu Guijun, Li Zhijun, Wang Qi, et al. Anti-inflammatory Components of Houttuynia cordata [J]. Journal of China Pharmaceutical University, 2016, 47(03): 294-298. |
| 94 | Azelaic acid | 11.30 | C9H16O4 | [M-H]- | 187.09753 | 5.583215515 | 169.08701、143.10762、125.09721 | Yuxingcao | Organic Acids | / | Mei Qianggen, Zhang Lu, Ma Tianxin, et al. Analysis of Antioxidant, Anti-diabetic Activities and Chemical Composition of the Extracted Fractions from Houttuynia cordata Aqueous Extract [J]. Food and Fermentation Industries, 2023, 49(11): 70-78. DOI:10.13995/j.cnki.11-1802/ts.030543. |
| 95 | Narcissin | 11.33 | [C28H32O16](https://pubchem.ncbi.nlm.nih.gov/#query=C28H32O16) | [M-H]- | 623.16217 | 2.42136594 | 315.05103、299.02173 | Jinyinhua | Flavonoids | / | Zhang X, Yu X, Sun X, Meng X, Fan J, Zhang F, Zhang Y. Comparative study on chemical constituents of different medicinal parts of Lonicera japonica Thunb. Based on LC-MS combined with multivariate statistical analysis. Heliyon. 2024 May 29;10(12):e31722. doi: 10.1016/j.heliyon.2024.e31722. PMID: 38975169; PMCID: PMC11225679. |
| 96 | 1,3-Dicaffeoylquinic acid | 11.67 | C25H24O12 | [M-H]- | 515.11938 | 1.897621974 | 191.05609、179.03497、135.04524 | Jinyinhua | Phenolic Acids | / | Jin Ying, Xiong Lewen, Pu Gaobin, et al. Comparison of Chemical Constituents between Lonicera fragrantissima Buds and Honeysuckle Based on Liquid Chromatography-Mass Spectrometry [J]. Chinese Traditional Patent Medicine, 2024, 46(03): 850-859. |
| 97 | [Galloylalbiflorin](https://www.chemsrc.com/en/cas/929042-36-4_1713718.html) | 11.71 | [C30H32O15](https://pubchem.ncbi.nlm.nih.gov/#query=C30H32O15) | [M-H]- | 631.16687 | 1.780039564 | 313.05566、169.01442、151.00366、121.02954 | Chishao | Monoterpenes | / | Li P,Zhang ZM,Li T,et al.Monoterpene derivatives from the roots of Paeonia lactiflora and their anti-proliferative activity. Fitoterapia.2014,Oct;98:124-9. |
| 98 | Rhoifolin* | 11.79 | C27H30O14 | [M+H]+ | 579.17029 | -0.935647947 | 271.05984、85.02827 | Jinyinhua | Flavonoids | Verified by reference standards. | Liu Shupeng, Huang Houyu, Wang Xiaoxue, et al. Revealing the Differences in Chemical Constituents and Anti-aging Mechanisms between Honeysuckle and Lonicerae Folium Based on UHPLC-QE-Orbitrap-MS Technology and Network Pharmacology [J]. Food Science, 2024, 45(20): 1-11. |
| 99 | Rosmarinic acid* | 12.07 | C18H16O8 | [M-H]- | 359.0771 | 2.662944939 | 197.04593、179.03471、161.02443、135.04514 | Bohe | Phenolic Acids | Verified by reference standards. | Xu Jingjing. Study on Quality Control and Evaluation Methods for Mentha Herb Based on Spectrum-Effect Relationship Analysis of Antioxidant Activity [D]. Beijing University of Chinese Medicine, 2014. |
| 100 | Hesperidin* | 12.08 | C28H34O15 | [M-H]- | 609.1704 | -18.05143765 | 325.07248、302.07526、301.07181、286.04721 | Bohe | Flavonoids | Verified by reference standards. | Xu Jingjing. Study on Quality Control and Evaluation Methods for Mentha Herb Based on Spectrum-Effect Relationship Analysis of Antioxidant Activity [D]. Beijing University of Chinese Medicine, 2014. |
| 101 | Diosmin | 12.12 | C28H32O15 | [M-H]- | 607.1673 | 2.558609422 | 299.05612、284.03351 | Jinyinhua、Bohe | Flavonoids | / | Wu Jiani, Xia Shuxue, Li Xuemei, et al. Fingerprint and Component Analysis of Mint Standard Decoction [J]. Chinese Journal of Experimental Traditional Medical Formulae, 2019, 25(16): 128-134. DOI:10.13422/j.cnki.syfjx.20191612. |
|  |  |  | C28H32O15 | [M-H]- | 607.1673 | 2.558609422 | 299.05612、284.03351 | Jinyinhua、Bohe | Flavonoids | / | Cai Z, Liao H, Wang C, Chen J, Tan M, Mei Y, Wei L, Chen H, Yang R, Liu X. A comprehensive study of the aerial parts of Lonicera japonica Thunb. based on metabolite profiling coupled with PLS-DA. Phytochem Anal. 2020 Nov;31(6):786-800. doi: 10.1002/pca.2943. Epub 2020 Apr 27. PMID: 32342594. |
| 102 | mudanpioside D | 12.22 | C24H30O12 | [M-H]- | 509.16647 | 2.194375548 | 463.21844、311.17642 | Chishao | Phenolic Glycosides | / | Liu Jie, Chen Lin, Fan Cairong, et al. Qualitative and Quantitative Study on Main Components of Paeoniae Radix Alba and Paeoniae Radix Rubra Based on HPLC-DAD-Q-TOF-MS/MS [J]. China Journal of Chinese Materia Medica, 2015, 40(09): 1762-1770. |
| 103 | Mudanpioside I | 12.25 | C23H28O11 | [M+HCOO]- | 525.16132 | 2.004530932 | 479.15762、283.08273、121.02955 | Chishao | Phenolic Glycosides | / | He Xiao, Wu Huimin, Xiong Lele, et al. Study on Monoterpene Components in Paeoniae Radix Rubra Based on UPLC-Q-TOF-MS Technology [J]. China Journal of Chinese Materia Medica, 2023, 48(04): 1005-1013. DOI:10.19540/j.cnki.cjcmm.20220728.201. |
| 104 | (Z)-Aldosecologanin | 12.38 | C34H46O19 | [M-H]- | 757.25604 | 1.432278511 | 595.19830、525.16174 | Jinyinhua | Iridoid Glycosides | / | Li XQ, Sun XH, Cai S, Ying XX, Li FM. Investigation on the chemical constituents and variation of the flower buds of Lonicera species by UPLC-ESI-MS/MS and principle component analysis. Yao Xue Xue Bao. 2009 Aug;44(8):895-904. PMID: 20055159. |
| 105 | (E)-Aldosecologanin | 12.4 | C34H46O19 | [M-H]- | 757.25598 | 1.353044959 | 595.20459、493.17303、179.05647 | Jinyinhua | Iridoid Glycosides | / | Zhang Y., Huang X., Zhao F., Tang Y., & Yin L. (2015). Study on the Chemical Markers of Caulis Lonicerae Japonicae for Quality Control By Hplc-qtof/ms/ms and Chromatographic Fingerprints Combined with Chemometrics Methods. Analytical Methods, 00, 2064-2076. http://dx.doi.org/10.1039/C4AY02744B. 10.1039/C4AY02744B. |
| 106 | Isochlorogenic acid C | 13.28 | C25H24O12 | [M-H]- | 515.11914 | 1.431709674 | 353.08725、191.05602、179.03496、161.02455、135.04547 | Jinyinhua、Aiye | Phenolic Acids | / | Lan Xiaoyan, Zhu Longbo, Huang Xianzhang, et al. Identification and Content Determination of Main Chemical Components in Artemisiae Argyi Folium [J]. Chinese Traditional and Herbal Drugs, 2021, 52(24): 7630-7637. |
|  |  |  | C25H24O12 | [M-H]- | 515.11914 | 1.431709674 | 353.08725、191.05602、179.03496、161.02455、135.04547 | Jinyinhua、Aiye | Phenolic Acids | / | Cai Z, Liao H, Wang C, Chen J, Tan M, Mei Y, Wei L, Chen H, Yang R, Liu X. A comprehensive study of the aerial parts of Lonicera japonica Thunb. based on metabolite profiling coupled with PLS-DA. Phytochem Anal. 2020 Nov;31(6):786-800. doi: 10.1002/pca.2943. Epub 2020 Apr 27. PMID: 32342594. |
| 107 | Lactiflorin | 13.37 | C23H26O10 | [M+HCOO]- | 507.15073 | 2.02583181 | 461.20343、177.05568、121.02953 | Chishao | Monoterpenes | / | Jin Zhongxian, Yu Jiahe, Liu Jinfeng, et al. Chemical Composition Analysis of Paeoniae Radix Rubra Based on UHPLC-Q-Exactive-Orbitrap-MS and Its Anti-psoriatic Activity Study [J]. Central South Pharmacy, 2023, 21(04): 894-902. |
| 108 | Calycosin* | 14.48 | C16H12O5 | [M-H]- | 283.0611 | 3.533171932 | 269.04114、268.03778、240.04114 | Jinyinhua | Flavonoids | Verified by reference standards. | Liu Hui, Wu Yuefeng, Zhu Zhifei, et al. Study on the "Heterogeneity but Equivalence" of Honeysuckle and Lonicerae Flos Based on Molecular Imprinting Technology [J]. China Journal of Traditional Chinese Medicine and Pharmacy, 2022, 37(02): 1027-1035. |
| 109 | Genkwanin | 14.52 | C16H12O5 | [M+H]+ | 285.07544 | -1.087430271 | 270.05225、225.05450 | Bohe | Flavonoids | / | Liu R, Wang Y, Liang C, Zheng Z, Du X, Cui Z, Zhang Y, Liu H. Morphology and mass spectrometry-based chemical profiling of peltate glandular trichomes on Mentha haplocalyx Briq leaves. Food Res Int. 2023 Feb;164:112323. doi: 10.1016/j.foodres.2022.112323. Epub 2022 Dec 10. PMID: 36737916. |
| 110 | Naringenin | 15.88 | C15H12O5 | [M-H]- | 271.0612 | 4.058509535 | 177.01929、151.00371 | Jinyinhua、Chishao、Aiye | Flavonoids | / | Tan R,Jia Z.Eudesmanolides and Other Constituents from Artemisia argyi.Planta Med.1992 Aug;58(4):370-2. |
|  |  |  | C15H12O5 | [M-H]- | 271.0612 | 4.058509535 | 177.01929、151.00371 | Jinyinhua、Chishao、Aiye | Flavonoids | / | Jin Zhongxian, Yu Jiahe, Liu Jinfeng, et al. Chemical Composition Analysis of Paeoniae Radix Rubra Based on UHPLC-Q-Exactive-Orbitrap-MS and Its Anti-psoriatic Activity Study [J]. Central South Pharmacy, 2023, 21(04): 894-902. |
|  |  |  | C15H12O5 | [M-H]- | 271.0612 | 4.058509535 | 177.01929、151.00371 | Jinyinhua、Chishao、Aiye | Flavonoids | / | Ge L,Xiao L,Wan H,et al.Chemical constituents from Lonicera japonica flower buds and their anti-hepatoma and anti-HBV activities. Bioorg Chem. 2019 Nov;92:103198. |
| 111 | 6'-O-benzoylpaeoniflorin | 17.15 | C30H32O12 | [M+HCOO]- | 629.18768 | 1.904045993 | 431.13733、195.06656、165.05565、121.02954 | Chishao | Monoterpenes | / | Shi YH, Zhu S, Ge YW, Toume K, Wang Z, Batkhuu J, Komatsu K. Characterization and quantification of monoterpenoids in different types of peony root and the related Paeonia species by liquid chromatography coupled with ion trap and time-of-flight mass spectrometry. J Pharm Biomed Anal. 2016 Sep 10;129:581-592. doi: 10.1016/j.jpba.2016.07.031. Epub 2016 Jul 20. PMID: 27521818. |
| 112 | Benzoylpaeoniflorin* | 17.18 | C30H32O12 | [M+NH4]+ | 602.22241 | -1.314960961 | 249.07564、179.07014、151.07533 | Chishao | Monoterpenes | Verified by reference standards. | Liu Jie, Chen Lin, Fan Cairong, et al. Qualitative and Quantitative Study on Main Components of Paeoniae Radix Alba and Paeoniae Radix Rubra Based on HPLC-DAD-Q-TOF-MS/MS [J]. China Journal of Chinese Materia Medica, 2015, 40(09): 1762-1770. |
| 113 | Mudanpioside J | 17.46 | [C31H34O14](https://pubchem.ncbi.nlm.nih.gov/#query=C31H34O14) | [M-H]- | 629.18774 | 1.999407228 | 165.0555、121.02951 | Chishao | Phenolic Glycosides | / | He Xiao, Wu Huimin, Xiong Lele, et al. Study on Monoterpene Components in Paeoniae Radix Rubra Based on UPLC-Q-TOF-MS Technology [J]. China Journal of Chinese Materia Medica, 2023, 48(04): 1005-1013. DOI:10.19540/j.cnki.cjcmm.20220728.201. |
| 114 | Stevioside | 17.62 | C38H60O18 | [M-H]- | 803.37024 | 0.807598404 | 641.31757、479.26425、317.21265 | Jinyinhua | Others | / | Liu Zhenying. Comparative Study on the Quality of Honeysuckle Medicinal Materials with Different Colors and Their Dynamic Changes During Storage [D]. China Academy of Chinese Medical Sciences, 2024. DOI:10.27658/d.cnki.gzzyy.2024.000101. |
| 115 | malyngic acid | 18.140 | C18H32O5 | [M-H]- | 327.21768 | 3.299038002 | 309.20685、291.19650、229.14452、211.13400、171.10269 | Chishao | Organic Acids | / | Jin Zhongxian, Yu Jiahe, Liu Jinfeng, et al. Chemical Composition Analysis of Paeoniae Radix Rubra Based on UHPLC-Q-Exactive-Orbitrap-MS and Its Anti-psoriatic Activity Study [J]. Central South Pharmacy, 2023, 21(04): 894-902. |
| 116 | Tricin | 10.84 | C17H14O7 | [M-H]- | 329.06662 | 3.162895379 | 229.14453、211.13397、183.13921、171.10260 | Jinyinhua | Flavonoids | / | Zhang X, Yu X, Sun X, Meng X, Fan J, Zhang F, Zhang Y. Comparative study on chemical constituents of different medicinal parts of Lonicera japonica Thunb. Based on LC-MS combined with multivariate statistical analysis. Heliyon. 2024 May 29;10(12):e31722. doi: 10.1016/j.heliyon.2024.e31722. PMID: 38975169; PMCID: PMC11225679. |
| 117 | arteether | 22.98 | C17H28O5 | [M-H]- | 311.18655 | 4.015613843 | 293.17566、267.19717 | Yuxingcao | Others | / | Ju L, Zhang J, Wang F, Zhu D, Pei T, He Z, Han Z, Wang M, Ma Y, Xiao W. Chemical profiling of Houttuynia cordata Thunb. by UPLC-Q-TOF-MS and analysis of its antioxidant activity in C2C12 cells. J Pharm Biomed Anal. 2021 Sep 10;204:114271. doi: 10.1016/j.jpba.2021.114271. Epub 2021 Jul 16. PMID: 34325249. |
| 118 | Tyrosine | 22.98 | C9H11NO3 | [M+H]+ | 182.08099 | -0.987471687 | 165.05370、136.07605、123.04409 | Jinyinhua | Amino Acids | / | Li Panlin, Li Chuyuan, Liu Menghua, et al. Comparison of Chemical Constituents between Honeysuckle and Lonicerae Flos Based on UFLC-Triple-Q-TOF-MS/MS Technology [J]. Central South Pharmacy, 2016, 14(04): 363-369. |
| 119 | Atractylodin* | 23.64 | C13H10O | [M+H]+ | 183.08049 | 0.264910875 | 183.08049、141.95818、113.96367、105.03343 | Jinyinhua | Others | Verified by reference standards. | Liu Qian, Fu Juan, Hu Junhua, et al. Chemical Component Analysis and Multi-index Quantitative Determination of Kechuanning Granules Based on HPLC-Q-TOF-MS/MS Technology [J]. Drug Evaluation Research, 2023, 46(05): 1012-1023. |
