# Supplementary material for: Exploring the mechanism of Shuangyu Granule in regulating immune-inflammatory responses in influenza through UPLC-Orbitrap-MS/MS, GC-MS, and network target analysis
Source: PLoS One. 2026 Jul 27;21(7):e0353259. doi: 10.1371/journal.pone.0353259 (PMC13405112; doi:10.1371/journal.pone.0353259)
Supplement: S2 Table — (DOCX) [file pone.0353259.s002.docx]

The in vitro chemical constituents of SYKL were identified using GC-MS


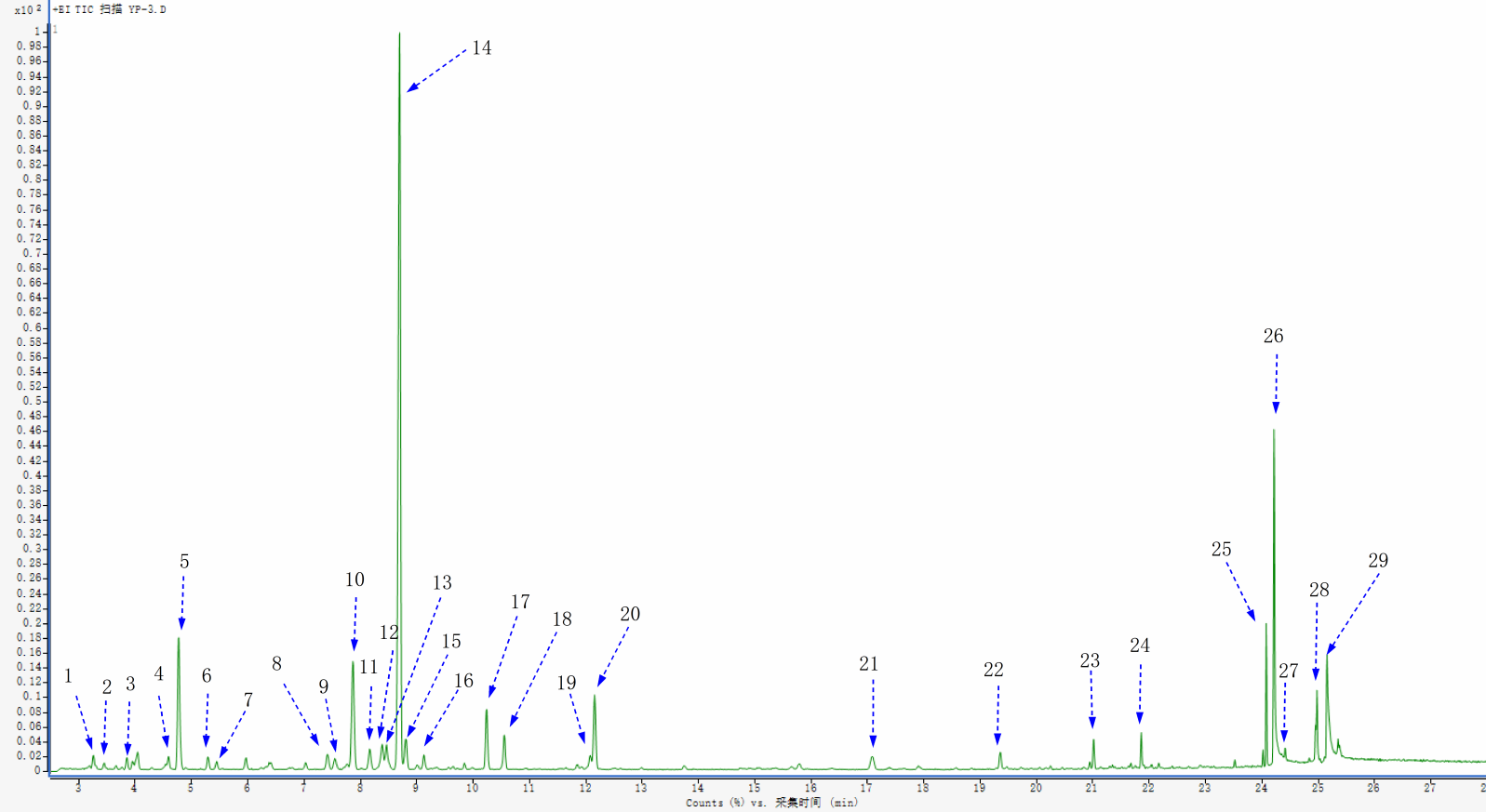


Figure. TIC chromatogram of volatile components in SYKL in vitro based on GC-MS, with the components corresponding to each peak listed in the table below.

| **No.** | **Identification** | **tR** | **Formulas** | **Source** | **Ref** |
| --- | --- | --- | --- | --- | --- |
| 1 | (+)-alpha-Pinene | 3.26 | C10H16 | Aiye | [Ning Erjuan, Li Xiao, Wang Wei, et al. Determination of Volatile Oil Components and Study on Antioxidant Activity of Artemisiae Argyi Folium from Nanyang with Different Aging Years [J/OL]. Journal of Chinese Medicinal Materials, 2024, (06): 1415-1419 [2025-04-28].https://doi.org/10.13863/j.issn1001-4454.2024.06.013.](https://doi.org/10.13863/j.issn1001-4454.2024.06.013) |
|  |  |  | C10H16 | Bohe | He Fengyan, He Yi, Wang Feifei, et al. Detection of Spearmint in Chinese Patent Medicines Containing Mentha by GC-MS/MS [J]. Chinese Traditional Patent Medicine, 2020, 42(02): 527-531. |
|  |  |  | C10H16 | Yuxingcao | Li Junmei, Zhao Yanglan, Hu Jiangning, et al. Prediction and Quantitative Analysis of Quality Markers in Houttuynia cordata Volatile Oil Based on GC Fingerprints and Chemometrics [J]. Strait Pharmaceutical Journal, 2024, 36(09): 18-24. |
| 2 | (+)-Camphene | 3.456 | C10H16 | Aiye | [Ning Erjuan, Li Xiao, Wang Wei, et al. Determination of Volatile Oil Components and Study on Antioxidant Activity of Artemisiae Argyi Folium from Nanyang with Different Aging Years [J/OL]. Journal of Chinese Medicinal Materials, 2024, (06): 1415-1419 [2025-04-28].https://doi.org/10.13863/j.issn1001-4454.2024.06.013.](https://doi.org/10.13863/j.issn1001-4454.2024.06.013) |
|  |  |  | C10H16 | Yuxingcao | Wang Bangyuan, Yang Yanfang, Pang Jianmei, et al. Simultaneous Determination of Nine Volatile Oil Components in Houttuynia cordata Extract by GC [J]. China Pharmacist, 2019, 22(07): 1261-1264. |
| 3 | Beta-pinene | 3.855 | C10H16 | Aiye | Zhang Wenjing, Li Haiyan, Wang Xiaowei, et al. Study on Differential Characteristic Components and Identification Rules of Artemisiae Argyi Folium and Its Adulterant Artemisia mongolica Leaves Based on Chemometrics and Gas Chromatography [J]. Chinese Journal of Pharmaceutical Analysis, 2024, 44(04): 649-662. DOI:10.16155/j.0254-1793.2024.04.12. |
|  |  |  | C10H16 | Bohe | He Fengyan, He Yi, Wang Feifei, et al. Detection of Spearmint in Chinese Patent Medicines Containing Mentha by GC-MS/MS [J]. Chinese Traditional Patent Medicine, 2020, 42(02): 527-531. |
|  |  |  | C10H16 | Yuxingcao | Wang Bangyuan, Yang Yanfang, Pang Jianmei, et al. Simultaneous Determination of Nine Volatile Oil Components in Houttuynia cordata Extract by GC [J]. China Pharmacist, 2019, 22(07): 1261-1264. |
| 4 | [m-cymene](https://www.chemsrc.com/en/cas/535-77-3_669123.html) | 4.594 | C10H14 | Yuxingcao | Li Junmei, Zhao Yanglan, Hu Jiangning, et al. Prediction and Quantitative Analysis of Quality Markers in Houttuynia cordata Volatile Oil Based on GC Fingerprints and Chemometrics [J]. Strait Pharmaceutical Journal, 2024, 36(09): 18-24. |
| 5 | Cineole | 4.775 | C10H18O | Aiye | Lin Xia, Cui Peichao, Wang Xue, et al. Fingerprint and Quantitative Analysis of Volatile Components in Shuangyu Granules [J]. Chinese Traditional and Herbal Drugs, 2019, 50(09): 2081-2086. |
|  |  |  | C10H18O | Bohe | Lin Xia, Cui Peichao, Wang Xue, et al. Fingerprint and Quantitative Analysis of Volatile Components in Shuangyu Granules [J]. Chinese Traditional and Herbal Drugs, 2019, 50(09): 2081-2086. |
| 6 | artemisyl ketone | 5.297 | C10H16O | Aiye | Zhang Wenjing, Li Haiyan, Wang Xiaowei, et al. Study on Differential Characteristic Components and Identification Rules of Artemisiae Argyi Folium and Its Adulterant Artemisia mongolica Leaves Based on Chemometrics and Gas Chromatography [J]. Chinese Journal of Pharmaceutical Analysis, 2024, 44(04): 649-662. DOI:10.16155/j.0254-1793.2024.04.12. |
| 7 | [γ-terpinene](https://www.chemsrc.com/en/cas/99-85-4_167556.html) | 5.45 | C10H16 | Aiye | Huang HC, Wang HF, Yih KH, Chang LZ, Chang TM. Dual bioactivities of essential oil extracted from the leaves of Artemisia argyi as an antimelanogenic versus antioxidant agent and chemical composition analysis by GC/MS. Int J Mol Sci. 2012 Nov 12;13(11):14679-97. doi: 10.3390/ijms131114679. PMID: 23203088; PMCID: PMC3509604. |
|  |  |  | C10H16 | Bohe | Zhang WJ,Yang K,You CX,et al.Contact toxicity and repellency of the essential oil from Mentha haplocalyx Briq.against Lasioderma serricorne.Chem Biodivers. 2015 May;12(5):832-9. |
| 8 | (+/-)-Camphor | 7.415 | C10H16O | Aiye | Lin Xia, Cui Peichao, Wang Xue, et al. Fingerprint and Quantitative Analysis of Volatile Components in Shuangyu Granules [J]. Chinese Traditional and Herbal Drugs, 2019, 50(09): 2081-2086. |
| 9 | laevo-pinocarveol | 7.545 | C10H16O | Aiye | Wang Jie, Chen Fei, Chen Guowei, et al. Preparation and Volatile Component Analysis of Essential Oil from Cultivated and Wild Artemisiae Argyi Folium in Linxia [J]. Fujian Agricultural Science and Technology, 2024, 55(11): 51-56. |
| 10 | (-)-menthone | 7.864 | C10H18O | Bohe | Lin Xia, Cui Peichao, Wang Xue, et al. Fingerprint and Quantitative Analysis of Volatile Components in Shuangyu Granules [J]. Chinese Traditional and Herbal Drugs, 2019, 50(09): 2081-2086. |
| 11 | iso-Mentone | 8.161 | C10H18O | Bohe | He Fengyan, He Yi, Wang Feifei, et al. Detection of Spearmint in Chinese Patent Medicines Containing Mentha by GC-MS/MS [J]. Chinese Traditional Patent Medicine, 2020, 42(02): 527-531. |
| 12 | borneol | 8.386 | C10H18O | Aiye | Lin Xia, Cui Peichao, Wang Xue, et al. Fingerprint and Quantitative Analysis of Volatile Components in Shuangyu Granules [J]. Chinese Traditional and Herbal Drugs, 2019, 50(09): 2081-2086. |
| 13 | neoisomenthol | 8.459 | C10H20O | Bohe | Liu Qi, Zhang Hua, Ye Yuanqing, et al. Study on Component Differences of Bursting Bead Flavors from Different Batches Using SPME-Arrow/GC-MS Combined with Multivariate Statistical Analysis [J]. Journal of Instrumental Analysis, 2025, 44(01): 135-144. |
| 14 | Menthol | 8.691 | C10H20O | Bohe | Lin Xia, Cui Peichao, Wang Xue, et al. Fingerprint and Quantitative Analysis of Volatile Components in Shuangyu Granules [J]. Chinese Traditional and Herbal Drugs, 2019, 50(09): 2081-2086. |
| 15 | (-)-terpinen-4-ol | 8.807 | C10H18O | Aiye | Guo Yuying, Chen Yulin, Wang Yifei. Study on the Determination of Eight Components in Artemisiae Argyi Folium Essential Oil by Quantitative Analysis of Multi-components by Single-marker (QAMS) [J]. Journal of Jinan University (Natural Science & Medicine Edition), 2024, 45(06): 641-650. |
|  |  |  | C10H18O | Yuxingcao | Jiao Jinying, Ji Xingyu, Li Zibo, et al. Analysis and Evaluation of Four Active Components in Houttuynia cordata from Different Habitats [J]. Jiangsu Agricultural Sciences, 2020, 48(01): 193-199. DOI:10.15889/j.issn.1002-1302.2020.01.037. |
| 16 | Terpineol | 9.126 | C10H18O | Aiye | Guo Yuying, Chen Yulin, Wang Yifei. Study on the Determination of Eight Components in Artemisiae Argyi Folium Essential Oil by Quantitative Analysis of Multi-components by Single-marker (QAMS) [J]. Journal of Jinan University (Natural Science & Medicine Edition), 2024, 45(06): 641-650. |
|  |  |  | C10H18O | Bohe | Xu Lulu. Chemical Characterization and In Vivo Metabolism Study of Mentha Water Extract [D]. Beijing University of Chinese Medicine, 2018. DOI:10.26973/d.cnki.gbjzu.2018.000141. |
|  |  |  | C10H18O | Yuxingcao | Jiao Jinying, Ji Xingyu, Li Zibo, et al. Analysis and Evaluation of Four Active Components in Houttuynia cordata from Different Habitats [J]. Jiangsu Agricultural Sciences, 2020, 48(01): 193-199. DOI:10.15889/j.issn.1002-1302.2020.01.037. |
| 17 | Pulegone | 10.235 | C10H16O | Bohe | Lin Xia, Cui Peichao, Wang Xue, et al. Fingerprint and Quantitative Analysis of Volatile Components in Shuangyu Granules [J]. Chinese Traditional and Herbal Drugs, 2019, 50(09): 2081-2086. |
| 18 | piperitone | 10.554 | C10H16O | Bohe | Ye Dan, Zhao Ming, Shao Yang, et al. Correlation Study on the Classification of Commercial Specifications of Mentha Herb Based on Chemical Analysis [J]. China Journal of Chinese Materia Medica, 2015, 40(02): 251-257. |
| 19 | Methyl nonyl ketone | 12.077 | C11H22O | Yuxingcao | Lin Xia, Cui Peichao, Wang Xue, et al. Fingerprint and Quantitative Analysis of Volatile Components in Shuangyu Granules [J]. Chinese Traditional and Herbal Drugs, 2019, 50(09): 2081-2086. |
| 20 | (-)-Menthyl Acetate | 12.156 | C12H22O2 | Bohe | Xu Yiming, Yue Wei, Sang Mengru, et al. Study on Commercial Quality and Differences of Mentha Herb from Different Producing Areas [J]. China Journal of Chinese Materia Medica, 2017, 42(17): 3391-3397. DOI:10.19540/j.cnki.cjcmm.2017.0132. |
| 21 | trans-Caryophyllene | 17.079 | C15H24 | Aiye | Guo Yuying, Chen Yulin, Wang Yifei. Study on the Determination of Eight Components in Artemisiae Argyi Folium Essential Oil by Quantitative Analysis of Multi-components by Single-marker (QAMS) [J]. Journal of Jinan University (Natural Science & Medicine Edition), 2024, 45(06): 641-650. |
|  |  |  | C15H24 | Bohe | He Fengyan, He Yi, Wang Feifei, et al. Detection of Spearmint in Chinese Patent Medicines Containing Mentha by GC-MS/MS [J]. Chinese Traditional Patent Medicine, 2020, 42(02): 527-531. |
|  |  |  | C15H24 | Yuxingcao | Li Junmei, Zhao Yanglan, Hu Jiangning, et al. Prediction and Quantitative Analysis of Quality Markers in Houttuynia cordata Volatile Oil Based on GC Fingerprints and Chemometrics [J]. Strait Pharmaceutical Journal, 2024, 36(09): 18-24. |
| 22 | beta-copaene | 19.356 | C15H24 | Aiye | Guo L, Zhang D, Wang L, Xue Z, Guo M, Duan L, Zheng Y. Comparison and Discrimination of Artemisia argyi and Artemisia lavandulifolia by Gas Chromatography-Mass Spectrometry-Based Metabolomic Approach. J AOAC Int. 2019 Nov 1;102(6):1814-1821. doi: 10.5740/jaoacint.19-0080. Epub 2019 Jul 9. PMID: 31288892. |
| 23 | Caryophyllene oxide | 21.016 | C15H24O | Aiye | [Ning Erjuan, Li Xiao, Wang Wei, et al. Determination of Volatile Oil Components and Study on Antioxidant Activity of Artemisiae Argyi Folium from Nanyang with Different Aging Years [J/OL]. Journal of Chinese Medicinal Materials, 2024, (06): 1415-1419 [2025-04-28].https://doi.org/10.13863/j.issn1001-4454.2024.06.013.](https://doi.org/10.13863/j.issn1001-4454.2024.06.013) |
|  |  |  | C15H24O | Yuxingcao | Jong T T,Jean M Y.Constituents of Houttuyniae cordata andthe crystal structure of vomifoliol[J].J Chin Chem Soc-Taip,1993,40(4):399-402. |
| 24 | [Neointermedeol](https://www.ncbi.nlm.nih.gov/pcsubstance/?term=) | 21.857 | C15H26O | Aiye | Guo L, Zhang D, Wang L, Xue Z, Guo M, Duan L, Zheng Y. Comparison and Discrimination of Artemisia argyi and Artemisia lavandulifolia by Gas Chromatography-Mass Spectrometry-Based Metabolomic Approach. J AOAC Int. 2019 Nov 1;102(6):1814-1821. doi: 10.5740/jaoacint.19-0080. Epub 2019 Jul 9. PMID: 31288892. |
| 25 | 1,2-Benzenedicarboxylic acid | 24.076 | C16H22O4 | Yuxingcao | Qi Shuai. Study on the Medicinal Quality of Houttuynia cordata and Its Suitable Growing Areas Based on Metabolomics and Network Pharmacology [D]. Shanghai Institute of Technology, 2023. DOI:10.27801/d.cnki.gshyy.2023.000522. |
| 26 | Palmitic acid | 24.214 | C16H32O2 | Aiye | Zhang Wenjing, Li Haiyan, Wang Xiaowei, et al. Study on Differential Characteristic Components and Identification Rules of Artemisiae Argyi Folium and Its Adulterant Artemisia mongolica Leaves Based on Chemometrics and Gas Chromatography [J]. Chinese Journal of Pharmaceutical Analysis, 2024, 44(04): 649-662. DOI:10.16155/j.0254-1793.2024.04.12. |
|  |  |  | C16H32O2 | Bohe | Xu Lulu. Chemical Characterization and In Vivo Metabolism Study of Mentha Water Extract [D]. Beijing University of Chinese Medicine, 2018. DOI:10.26973/d.cnki.gbjzu.2018.000141. |
|  |  |  | C16H32O2 | Yuxingcao | Meng Jie, Zhang Cun, Wang Lijun, et al. Effects of Different Drying Methods on Volatile Components in Houttuynia cordata [J]. West China Journal of Pharmaceutical Sciences, 2022, 37(04): 398-408. DOI:10.13375/j.cnki.wcjps.2022.04.010. |
| 27 | Ethyl palmitate | 24.409 | C18H36O2 | Aiye | Du Jiajun, Gao Rui, Wang Shaosheng, et al. GC-MS Analysis of Volatile Oil Components in Artemisiae Argyi Folium from Anhui [J]. Journal of Wannan Medical College, 2017, 36(01): 11-15. |
| 28 | Methyl Linolenate | 24.975 | C19H32O2 | Bohe | Xu Lulu. Chemical Characterization and In Vivo Metabolism Study of Mentha Water Extract [D]. Beijing University of Chinese Medicine, 2018. DOI:10.26973/d.cnki.gbjzu.2018.000141. |
| 29 | Linolelaidic acid | 25.163 | C18H32O2 | Bohe | Ping Sheng, Zhu Caihui, Yan Ting, et al. Comparative Study on Volatile Oil Components of Different Parts of Mentha [J]. Journal of Wuhan Polytechnic University, 2015, 34(02): 31-35. |
