# Supplementary material for: Exploring the mechanism of Shuangyu Granule in regulating immune-inflammatory responses in influenza through UPLC-Orbitrap-MS/MS, GC-MS, and network target analysis
Source: PLoS One. 2026 Jul 27;21(7):e0353259. doi: 10.1371/journal.pone.0353259 (PMC13405112; doi:10.1371/journal.pone.0353259)
Supplement: S3 Table — (DOCX) [file pone.0353259.s003.docx]

| \| The absorbed components of Shuangyu Keli in blood were identified using UPLC-Q-E-MS/MS. \| \| \| \| \| \| \| \| \| \| \| \| \| --- \| --- \| --- \| --- \| --- \| --- \| --- \| --- \| --- \| --- \| --- \| --- \| \| No. \| **Identification** \| tR \| Formulas \| Selected ion \| Measured \| ppm \| Fragmentation \| **Source** \| Types \| Remarks \| **Ref** \| \| \| 1 \| Quinic acid \| 0.73 \| C7H12O6 \| [M-H]- \| 191.05594 \| 4.844154457 \| 127.04015、93.03468、87.00880、85.02958、59.01382 \| Jinyinhua、Yuxingcao、Aiye \| Organic Acids \| / \| Han B,Xin Z,Ma S,*et al*.Comprehensive characterization and identification of antioxidants in Folium Artemisiae Argyi using high-resolution tandem mass spectrometry.J Chromatogr B Analyt Technol Biomed Life Sci.2017 Sep 15;1063:84-92. \| \| \| C7H12O6 \| [M-H]- \| 191.05594 \| 4.844154457 \| 127.04015、93.03468、87.00880、85.02958、59.01382 \| / \| Zhang Y,Huang X,Zhao F,et al.Study on the Chemical Markers of Caulis Lonicerae Japonicae for Quality Control By Hplc-qtof/ms/ms and Chromatographic Fingerprints Combined with Chemometrics Methods. Analytical Methods, 00, 2064-2076. http://dx.doi.org/10.1039/C4AY02744B. 10.1039/C4AY02744B. \| \| \| C7H12O6 \| [M-H]- \| 191.05594 \| 4.844154457 \| 127.04015、93.03468、87.00880、85.02958、59.01382 \| / \| [Mei Q, Zhang L, Ma T, et al. Analysis of antioxidant and anti-diabetic activities and chemical composition of extracted components from Houttuynia cordata water extract. Food and Fermentation Industries, 49(11), 70–78. https://doi.org/10.13995/j.cnki.11-1802/ts.030543](https://doi.org/10.13995/j.cnki.11-1802/ts.030543) \| \| \| 2 \| Gallic acid* \| 1.25 \| C7H6O5 \| [M-H]- \| 169.01418 \| 6.095975383 \| 126.02795、125.02444、97.02960、81.03466、69.03469 \| Yuxingcao、Chishao \| Phenolic Acids \| Verified by reference standards. \| Xiong P,Qin S,Li K,et al.Identification of the Tannins in Traditional Chinese Medicine Paeoniae Radix Alba By UHPLC-Q-Exactive Orbitrap Ms. Arabian Journal of Chemistry,14,103398. \| \| \| C7H6O5 \| [M-H]- \| 169.01418 \| 6.095975383 \| 126.02795、125.02444、97.02960、81.03466、69.03469 \| Verified by reference standards. \| Ju L, Zhang J, Wang F, Zhu D, Pei T, He Z, Han Z, Wang M, Ma Y, Xiao W. Chemical profiling of Houttuynia cordata Thunb. by UPLC-Q-TOF-MS and analysis of its antioxidant activity in C2C12 cells. J Pharm Biomed Anal. 2021 Sep 10;204:114271. doi: 10.1016/j.jpba.2021.114271. Epub 2021 Jul 16. PMID: 34325249. \| \| \| 3 \| Loganic acid* \| 3.89 \| C16H24O10 \| [M-H]- \| 375.12939 \| 2.177120215 \| 213.07689、169.08708、113.02455、69.03466 \| Jinyinhua \| Iridoid Glycosides \| Verified by reference standards. \| [Gong X, Liu, W, Cao L, et al. Rapid qualitative analysis of chemical constituents in Lonicerae Japonicae Flos by DI-MS/MSALL. China Journal of Chinese Materia Medica,2021,46(09):2220-2228.DOI:10.19540/j.cnki.cjcmm.20210220.302.](https://doi.org/10.19540/j.cnki.cjcmm.20210220.302) \| \| \| 4 \| Chlorogenic acid* \| 4.17 \| C16H18O9 \| [M-H]- \| 353.08719 \| 1.363687696 \| 191.05606、179.03519、170.94159、161.02452、135.04543 \| Jinyinhua、Yuxingcao、Chishao、Aiye、Bohe \| Phenolic Acids \| Verified by reference standards. \| Lan X, Zhu L, Huang X, et al. Identification and content determination of main chemical components in Artemisiae Argyi Folium. *Chinese Traditional and Herbal Drugs*,2021,52(24):7630-7637. \| \| \| C16H18O9 \| [M-H]- \| 353.08719 \| 1.363687696 \| 191.05606、179.03519、170.94159、161.02452、135.04543 \| Verified by reference standards. \| [Wu J, Xia S, Li X, et al.Fingerprint and component analysis of Mint standard decoction. Chinese Journal of Experimental Traditional Medical Formulae,2019,25(16):128-134.DOI:10.13422/j.cnki.syfjx.20191612.](https://doi.org/10.13422/j.cnki.syfjx.20191612) \| \| \| C16H18O9 \| [M-H]- \| 353.08719 \| 1.363687696 \| 191.05606、179.03519、170.94159、161.02452、135.04543 \| Verified by reference standards. \| Fu M,Sang X,Cheng H.Total glucosides of peony induce fibroblast-like synovial apoptosis, and ameliorate cartilage injury via blocking the NF-κB/STAT3 pathway.Ann Transl Med.2022 Jan;10(2):51. \| \| \| C16H18O9 \| [M-H]- \| 353.08719 \| 1.363687696 \| 191.05606、179.03519、170.94159、161.02452、135.04543 \| Verified by reference standards. \| Ge L,Wan H,Tang S,*et al*.Novel caffeoylquinic acid derivatives from Lonicera japonica Thunb. flower buds exert pronounced anti-HBV activities. RSC Adv. 2018 Oct 15;8(62):35374-35385. \| \| \| C16H18O9 \| [M-H]- \| 353.08719 \| 1.363687696 \| 191.05606、179.03519、170.94159、161.02452、135.04543 \| Verified by reference standards. \| Wu X, Li S, Li A, et al.Study on the chemical constituents of Houttuynia cordata. *Journal of Chinese Medicinal Materials*,2008,(08):1168-1170. \| \| \| 5 \| 8-Epi loganic acid \| 4.48 \| [C16H24O10](https://pubchem.ncbi.nlm.nih.gov/#query=C16H24O10) \| [M-H]- \| 375.12930 \| 1.937202473 \| 169.08688、151.07634、59.01384 \| Jinyinhua \| Iridoid Glycosides \| / \| Cai Z, Liao H, Wang C, Chen J, Tan M, Mei Y, Wei L, Chen H, Yang R, Liu X. A comprehensive study of the aerial parts of Lonicera japonica Thunb. based on metabolite profiling coupled with PLS-DA. Phytochem Anal. 2020 Nov;31(6):786-800. doi: 10.1002/pca.2943. Epub 2020 Apr 27. PMID: 32342594. \| \| \| 6 \| Cryptochlorogenic acid* \| 4.75 \| C16H18O9 \| [M-H]- \| 353.0875 \| 2.241659006 \| 191.05620、179.03505、173.04561、155.03517、135.04527 \| Yuxingcao、Bohe、Aiye \| Phenolic Acids \| Verified by reference standards. \| Lan X, Zhu L, Huang X, et al. Identification and content determination of main chemical components in Artemisiae Argyi Folium. *Chinese Traditional and Herbal Drugs*, 52(24), 7630–7637. (Duplicate of #7, kept for completeness) \| \| \| C16H18O9 \| [M-H]- \| 353.0875 \| 2.241659006 \| 191.05620、179.03505、173.04561、155.03517、135.04527 \| Verified by reference standards. \| Liu R,Wang Y,Liang C,et al.Morphology and mass spectrometry-based chemical profiling of peltate glandular trichomes on Mentha haplocalyx Briq leaves.Food Res Int.2023 Feb;164:112323. \| \| \| C16H18O9 \| [M-H]- \| 353.0875 \| 2.241659006 \| 191.05620、179.03505、173.04561、155.03517、135.04527 \| Verified by reference standards. \| Ahn J,Kim J.Chemical constituents from Houttuynia cordata[J].Planta Med,2016,81(S1):S1-S381. \| \| \| 7 \| secologanoside \| 4.8 \| C16H22O11 \| [M-H]- \| 389.10855 \| 1.830340925 \| 209.04498、183.06647、165.05577、121.06598 \| Jinyinhua \| Iridoid Glycosides \| / \| Ren MT, Chen J, Song Y, Sheng LS, Li P, Qi LW. Identification and quantification of 32 bioactive compounds in Lonicera species by high performance liquid chromatography coupled with time-of-flight mass spectrometry. J Pharm Biomed Anal. 2008 Dec 15;48(5):1351-60. doi: 10.1016/j.jpba.2008.09.037. Epub 2008 Sep 30. PMID: 18977626. \| \| \| 8 \| Swertiamarin \| 5.09 \| C16H22O10 \| [M-H]- \| 373.11362 \| 1.867531133 \| 193.05054、149.06064、119.03494、97.02953、89.02451 \| Jinyinhua \| / \| Jin Y, Xiong L, Pu G, et al. Comparison of chemical constituents between Lonicera fragrantissima buds and Lonicerae Japonicae Flos based on LC-MS technology. *Chinese Traditional Patent Medicine*,2024,46(03):850-859. \| \| \| 9 \| Norisoboldine \| 5.22 \| C18H19NO4 \| [M+H]+ \| 314.13849 \| -0.61947162 \| 297.11157、282.08734、265.08594 \| Yuxingcao \| Alkaloids \| / \| Ju L, Zhang J, Wang F, Zhu D, Pei T, He Z, Han Z, Wang M, Ma Y, Xiao W. Chemical profiling of Houttuynia cordata Thunb. by UPLC-Q-TOF-MS and analysis of its antioxidant activity in C2C12 cells. J Pharm Biomed Anal. 2021 Sep 10;204:114271. doi: 10.1016/j.jpba.2021.114271. Epub 2021 Jul 16. PMID: 34325249. \| \| \| 10 \| isoboldine \| 5.98 \| C19H21NO4 \| [M+H]+ \| 328.15414 \| -0.593318385 \| 297.11206、265.08594、237.09109 \| Yuxingcao \| / \| [Wu Y. Study on alkaloid components and their safety in Houttuynia cordata [Master's thesis, National Institutes for Food and Drug Control]. China Master's Theses Full-text Database. https://doi.org/10.27651/d.cnki.gzyss.2022.000006](https://doi.org/10.27651/d.cnki.gzyss.2022.000006) \| \| \| 11 \| Sweroside \| 6.38 \| C16H22O9 \| [M+HCOO]- \| 403.12427 \| 1.940100301 \| 195.06708、151.07648、125.02451 \| Jinyinhua \| Iridoid Glycosides \| / \| Cai Z, Liao H, Wang C, Chen J, Tan M, Mei Y, Wei L, Chen H, Yang R, Liu X. A comprehensive study of the aerial parts of Lonicera japonica Thunb. based on metabolite profiling coupled with PLS-DA. Phytochem Anal. 2020 Nov;31(6):786-800. doi: 10.1002/pca.2943. Epub 2020 Apr 27. PMID: 32342594. \| \| \| 12 \| Albiflorin* \| 6.62 \| C23H28O11 \| [M+H]+ \| 481.16974 \| -1.45083726 \| 197.08069、179.07021、151.07533、133.06477、105.03343 \| Chishao \| Monoterpenes \| Verified by reference standards. \| Liu J, Chen L, Fan C, et al. Qualitative and quantitative study on main constituents of Paeoniae Radix Alba and Paeoniae Radix Rubra based on HPLC-DAD-Q-TOF-MS/MS. *China Journal of Chinese Materia Medica*, 2015,40(09):1762-1770. \| \| \| 13 \| Ferulaldehyde \| 7.47 \| C10H10O3 \| [M+H]+ \| 179.0699 \| -2.070137039 \| 161.06000、133.06468、105.06976 \| Chishao \| Others \| / \| [Fan W, Liao C, Zhou Y, et al. Study on the chemical constituents and anti-inflammatory activity of Paeoniae Radix Rubra. Journal of Kunming University of Science and Technology (Natural Science Edition),2022,47(03):117-122.DOI:10.16112/j.cnki.53-1223/n.2022.03.252.](https://doi.org/10.16112/j.cnki.53-1223/n.2022.03.252) \| \| \| 14 \| Paeoniflorin* \| 7.48 \| C23H28O11 \| [M+NH4]+ \| 498.1967 \| -0.576478797 \| 179.07014、151.07524 \| Chishao \| Monoterpenes \| Verified by reference standards. \| Liu J, Chen L, Fan C, et al. Qualitative and quantitative study on main constituents of Paeoniae Radix Alba and Paeoniae Radix Rubra based on HPLC-DAD-Q-TOF-MS/MS. *China Journal of Chinese Materia Medica*, 2015,40(09):1762-1770. \| \| \| 15 \| Secoxyloganin \| 8.08 \| C17H24O11 \| [M-H]- \| 403.12418 \| 1.716843649 \| 371.09772、223.06122、165.05562、149.02438、121.02953 \| Jinyinhua \| Iridoid Glycosides \| / \| Cai Z, Liao H, Wang C, Chen J, Tan M, Mei Y, Wei L, Chen H, Yang R, Liu X. A comprehensive study of the aerial parts of Lonicera japonica Thunb. based on metabolite profiling coupled with PLS-DA. Phytochem Anal. 2020 Nov;31(6):786-800. doi: 10.1002/pca.2943. Epub 2020 Apr 27. PMID: 32342594. \| \| \| 16 \| Isofraxidin* \| 8.680 \| C11H10O5 \| [M+H]+ \| 223.05988 \| -0.985832967 \| 208.03752、190.02638 \| Aiye \| Others \| Verified by reference standards. \| Kim KO,Lee D,Hiep NT,et al.Protective Effect of Phenolic Compounds Isolated from Mugwort(Artemisia argyi)against Contrast-Induced Apoptosis in Kidney Epithelium Cell Line LLC-PK1.Molecules.2019 Jan 7;24(1):195. \| \| \| 17 \| Calycosin* \| 14.48 \| C16H12O5 \| [M-H]- \| 283.0611 \| 3.533171932 \| 269.04114、268.03778、240.04114 \| Jinyinhua \| Flavonoids \| Verified by reference standards. \| Liu H, Wu Y, Zhu Z, et al. Study on the "heterogeneous equivalence" of Lonicerae Japonicae Flos and Lonicerae Flos based on molecular imprinting technology. *China Journal of Traditional Chinese Medicine and Pharmacy*,2022,37(02):1027-1035. \| \| \| 18 \| Genkwanin \| 14.52 \| C16H12O5 \| [M+H]+ \| 285.07544 \| -1.087430271 \| 270.05225、225.05450 \| Bohe \| / \| Liu R, Wang Y, Liang C, Zheng Z, Du X, Cui Z, Zhang Y, Liu H. Morphology and mass spectrometry-based chemical profiling of peltate glandular trichomes on Mentha haplocalyx Briq leaves. Food Res Int. 2023 Feb;164:112323. doi: 10.1016/j.foodres.2022.112323. Epub 2022 Dec 10. PMID: 36737916. \| \| \| 19 \| Atractylodin* \| 23.64 \| C13H10O \| [M+H]+ \| 183.08049 \| 0.264910875 \| 183.08049、141.95818、113.96367、105.03343 \| Jinyinhua \| Others \| Verified by reference standards. \| Liu Q, Fu J, Hu J, et al. Chemical composition analysis and multi-index quantitative determination of Kechuanning Granules based on HPLC-Q-TOF-MS/MS technology. *Drug Evaluation Research*, 2023,46(05):1012-1023. \| \| \| The absorbed components of Shuangyu Keli in blood were identified using GC/MS. \| \| \| \| \| \| \| \| \| \| \| \| \| 20 \| Cineole* \| 4.74 \| C10H18O \|  \|  \|  \| 108.264、81.211、71.261、43.775 \| Aiye \| Essential Oils \| Verified by reference standards. \| Lin X, Cui P, Wang X, et al. Fingerprint and quantitative analysis of volatile components in Shuangyu Granules. *Chinese Traditional and Herbal Drugs*, 2019,50(09):2081-2086. \| \| \| C10H18O \|  \|  \|  \| 108.264、81.211、71.261、43.775 \| Bohe \| Essential Oils \| Verified by reference standards. \| Lin X, Cui P, Wang X, et al. Fingerprint and quantitative analysis of volatile components in Shuangyu Granules. *Chinese Traditional and Herbal Drugs*, 2019,50(09):2081-2086. \| \| \| 21 \| Menthol* \| 8.64 \| C10H20O \|  \|  \|  \| 95.217/81.185/71.223、55.108 \| Bohe \| Essential Oils \| Verified by reference standards. \| Lin X, Cui P, Wang X, et al. Fingerprint and quantitative analysis of volatile components in Shuangyu Granules. *Chinese Traditional and Herbal Drugs*, 2019,50(09):2081-2086. \| \| |
| --- | --- | --- | --- | --- | --- | --- | --- | --- | --- | --- | --- | --- | --- | --- | --- | --- | --- | --- | --- | --- | --- | --- | --- | --- | --- | --- | --- | --- | --- | --- | --- | --- | --- | --- | --- | --- | --- | --- | --- | --- | --- | --- | --- | --- | --- | --- | --- | --- | --- | --- | --- | --- | --- | --- | --- | --- | --- | --- | --- | --- | --- | --- | --- | --- | --- | --- | --- | --- | --- | --- | --- | --- | --- | --- | --- | --- | --- | --- | --- | --- | --- | --- | --- | --- | --- | --- | --- | --- | --- | --- | --- | --- | --- | --- | --- | --- | --- | --- | --- | --- | --- | --- | --- | --- | --- | --- | --- | --- | --- | --- | --- | --- | --- | --- | --- | --- | --- | --- | --- | --- | --- | --- | --- | --- | --- | --- | --- | --- | --- | --- | --- | --- | --- | --- | --- | --- | --- | --- | --- | --- | --- | --- | --- | --- | --- | --- | --- | --- | --- | --- | --- | --- | --- | --- | --- | --- | --- | --- | --- | --- | --- | --- | --- | --- | --- | --- | --- | --- | --- | --- | --- | --- | --- | --- | --- | --- | --- | --- | --- | --- | --- | --- | --- | --- | --- | --- | --- | --- | --- | --- | --- | --- | --- | --- | --- | --- | --- | --- | --- | --- | --- | --- | --- | --- | --- | --- | --- | --- | --- | --- | --- | --- | --- | --- | --- | --- | --- | --- | --- | --- | --- | --- | --- | --- | --- | --- | --- | --- | --- | --- | --- | --- | --- | --- | --- | --- | --- | --- | --- | --- | --- | --- | --- | --- | --- | --- | --- | --- | --- | --- | --- | --- | --- | --- | --- | --- | --- | --- | --- | --- | --- | --- | --- | --- | --- | --- | --- | --- | --- | --- | --- | --- | --- | --- | --- | --- | --- | --- | --- | --- | --- | --- | --- | --- | --- | --- | --- | --- | --- | --- | --- | --- | --- | --- | --- | --- | --- | --- | --- | --- | --- | --- | --- | --- | --- | --- | --- | --- | --- | --- | --- | --- | --- | --- | --- | --- | --- | --- | --- | --- | --- | --- | --- | --- | --- | --- | --- | --- | --- | --- | --- | --- | --- | --- | --- | --- | --- | --- | --- | --- | --- | --- | --- | --- | --- | --- | --- | --- | --- | --- | --- | --- | --- | --- | --- | --- | --- | --- | --- | --- | --- | --- | --- | --- | --- | --- | --- | --- | --- | --- | --- | --- | --- | --- | --- | --- | --- | --- | --- | --- | --- | --- | --- | --- | --- | --- | --- | --- | --- |
