# Supplementary material for: Exploring the mechanism of Shuangyu Granule in regulating immune-inflammatory responses in influenza through UPLC-Orbitrap-MS/MS, GC-MS, and network target analysis
Source: PLoS One. 2026 Jul 27;21(7):e0353259. doi: 10.1371/journal.pone.0353259 (PMC13405112; doi:10.1371/journal.pone.0353259)

ibrary(Seurat)

library(SeuratObject)

library(ggplot2)

library(cowplot)

library(Matrix)

library(dplyr)

library(ggsci)

AI01<-Read10X("../GSM7792039/")

AI02<-Read10X("../GSM7792040/")

HC01<-Read10X("../GSM7792046/")

HC02<-Read10X("../GSM7792047/")


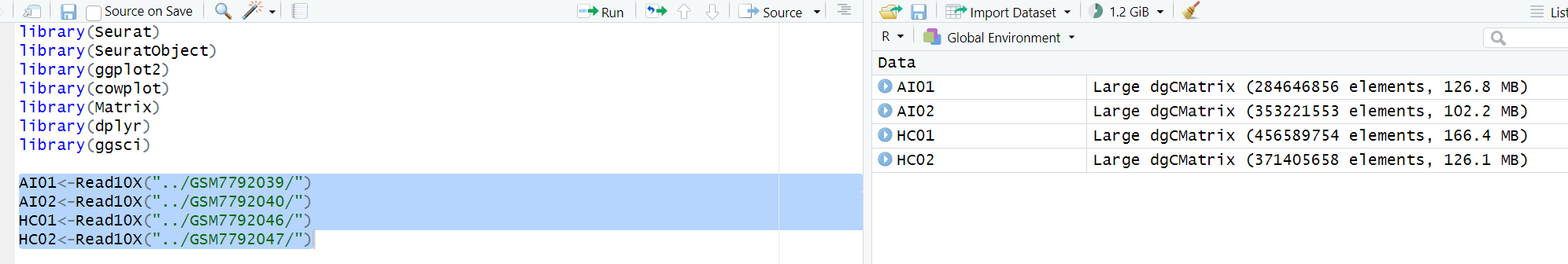


dim(AI01)

dim(AI02)

dim(HC01)

dim(HC02)


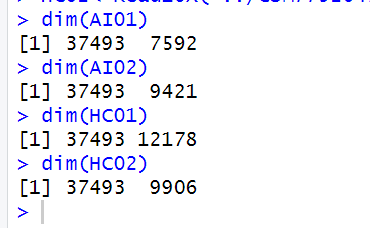


colnames(AI01) <- paste(colnames(AI01),"AI01",sep = "_")

colnames(AI02) <- paste(colnames(AI02),"AI02",sep = "_")

colnames(HC01) <- paste(colnames(HC01),"HC01",sep = "_")

colnames(HC02) <- paste(colnames(HC02),"HC02",sep = "_")

colnames(AI01)[1:10]

rownames(AI01)[1:10]


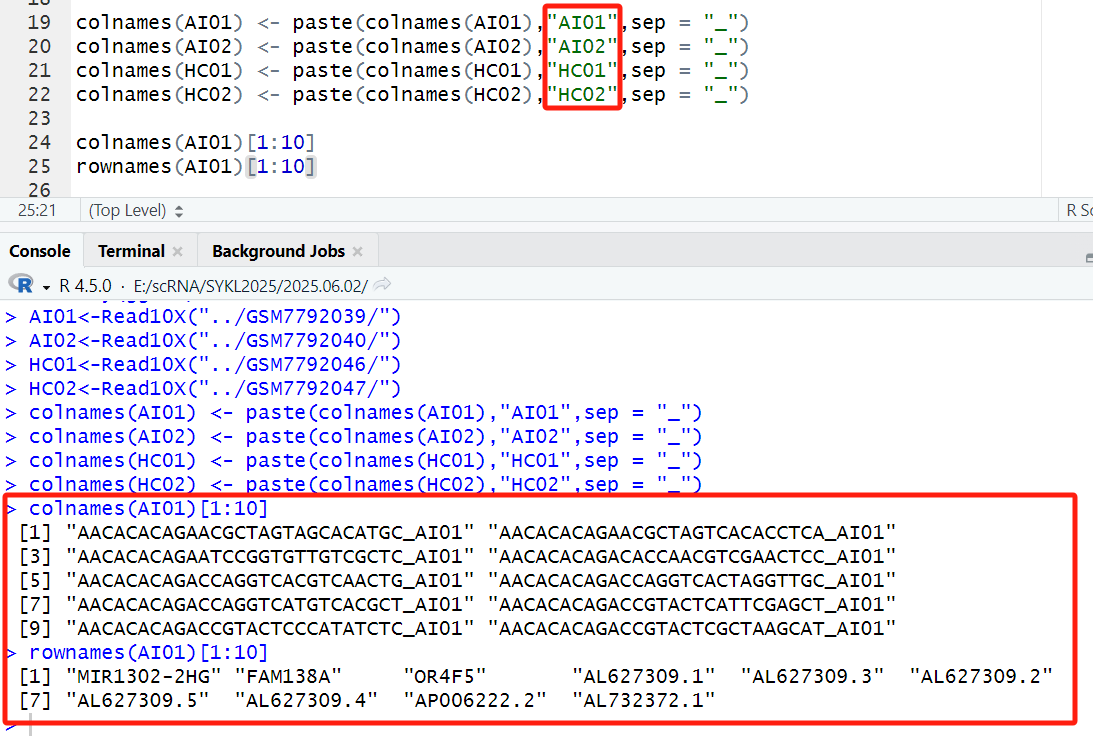


AH<-cbind(AI01,AI02,HC01,HC02)

rm(AI01)

rm(AI02)

rm(HC01)

rm(HC02)

sam.name <- "result"

if(!dir.exists(sam.name)){

dir.create(sam.name)

}

AH<- CreateSeuratObject(

AH,

project = "result",

min.cells = 10,

min.features = 200,

names.field = 2,

names.delim = "_")

save(AH,file=paste0("./",sam.name,"/",sam.name,"_raw_R_AH.RData"))

slotNames(AH)

AH@assays

dim(AH@meta.data)


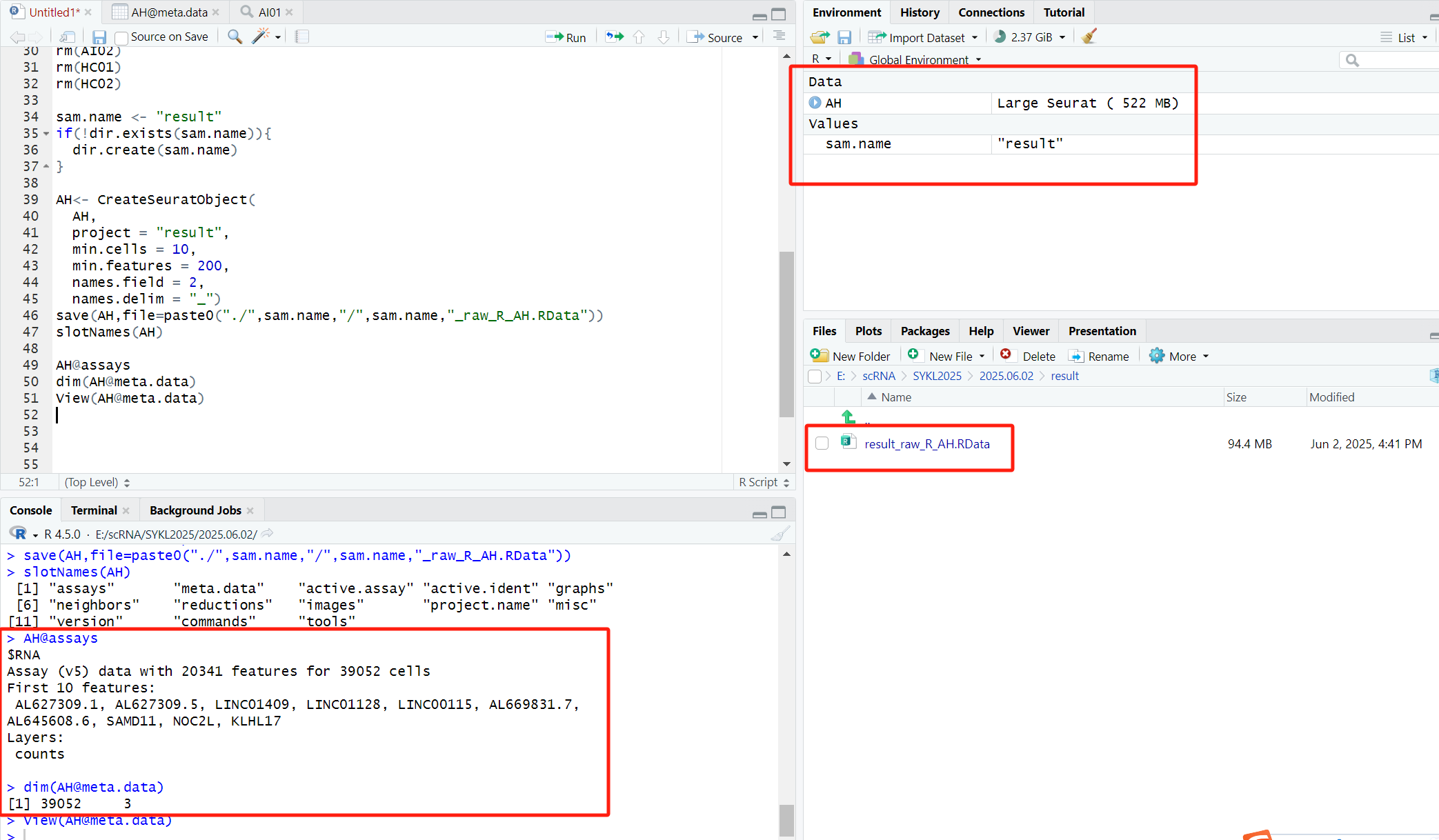


View(AH@meta.data)


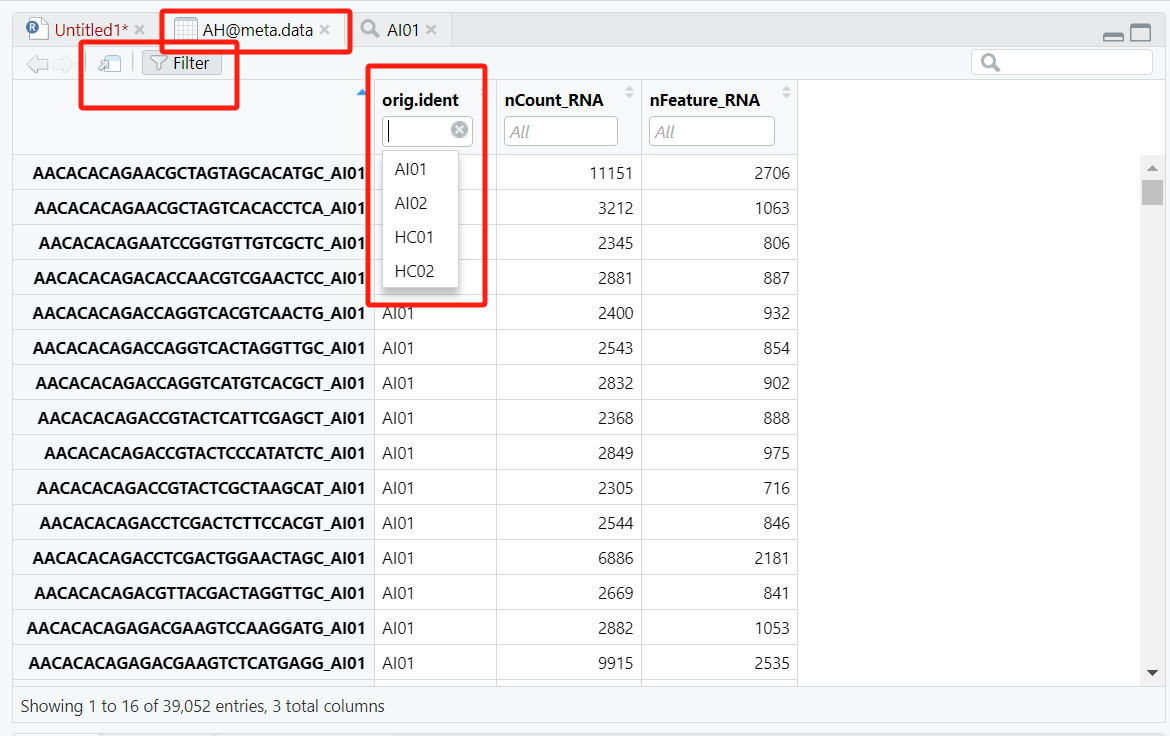


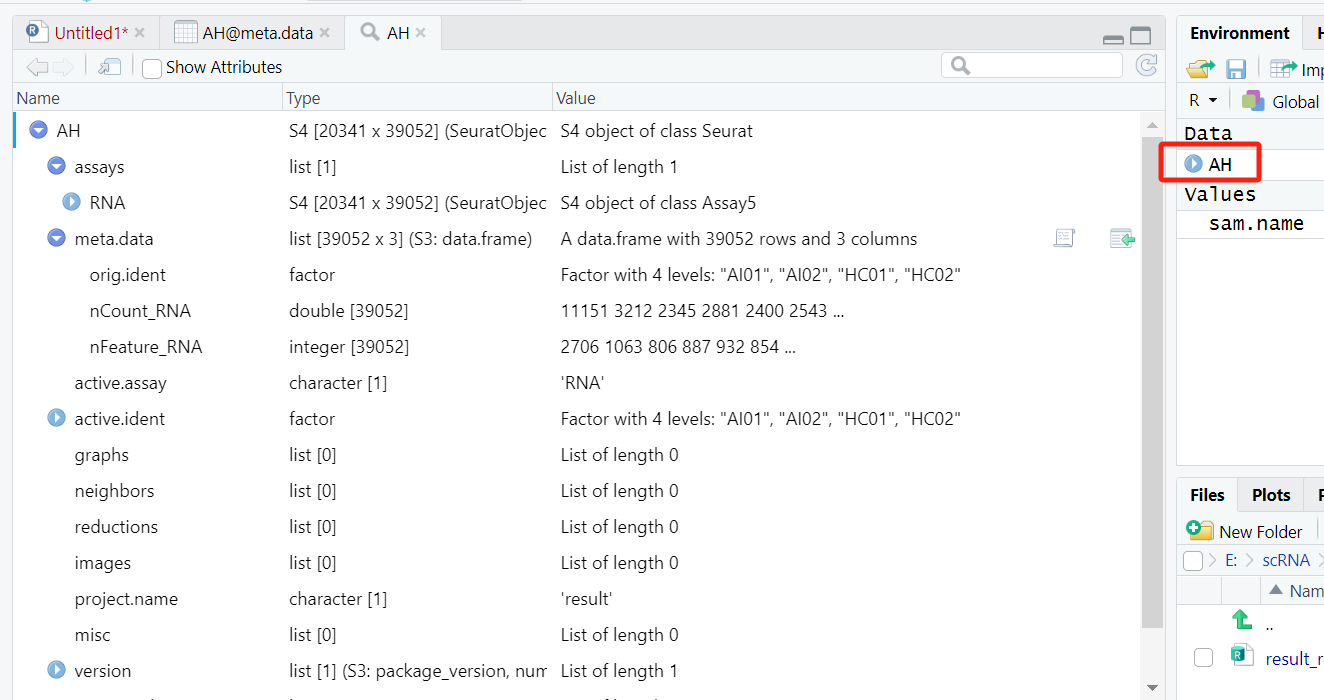


AH[["percent.mt"]] <- PercentageFeatureSet(AH,

pattern = "^MT-")

View(AH@meta.data)


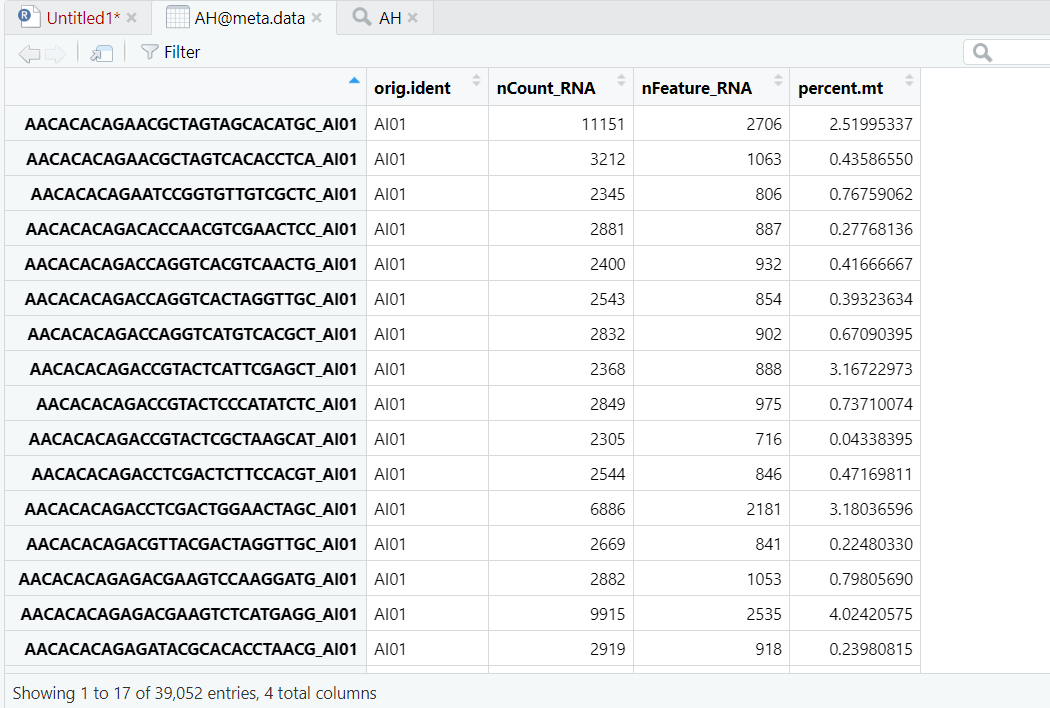


pdf(paste0("./",sam.name,"/QC-VlnPlot.pdf"),width = 8,height = 4.5)

VlnPlot(AH, features = c("nFeature_RNA", "nCount_RNA", "percent.mt"),

ncol = 3)

dev.off()


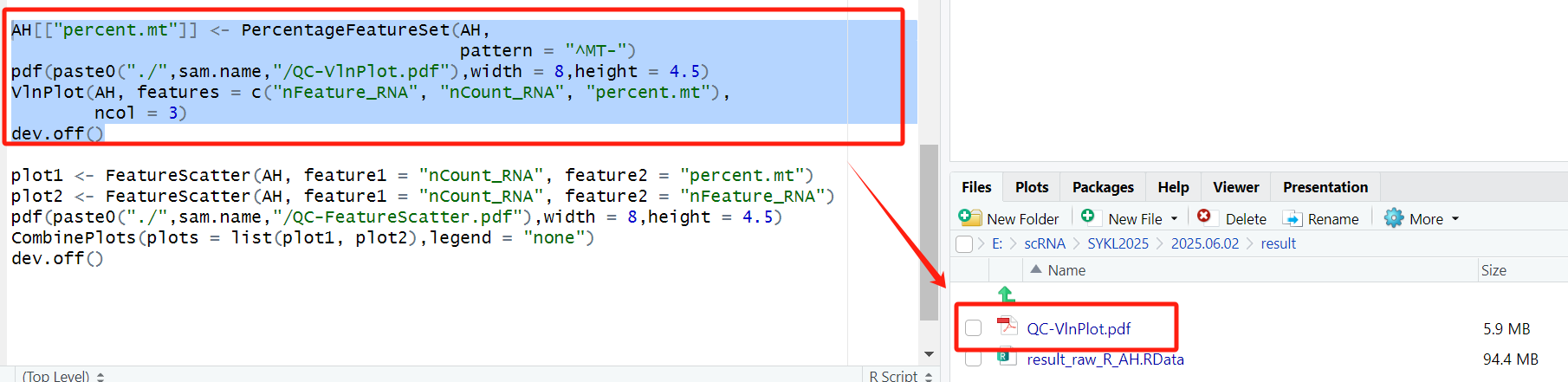


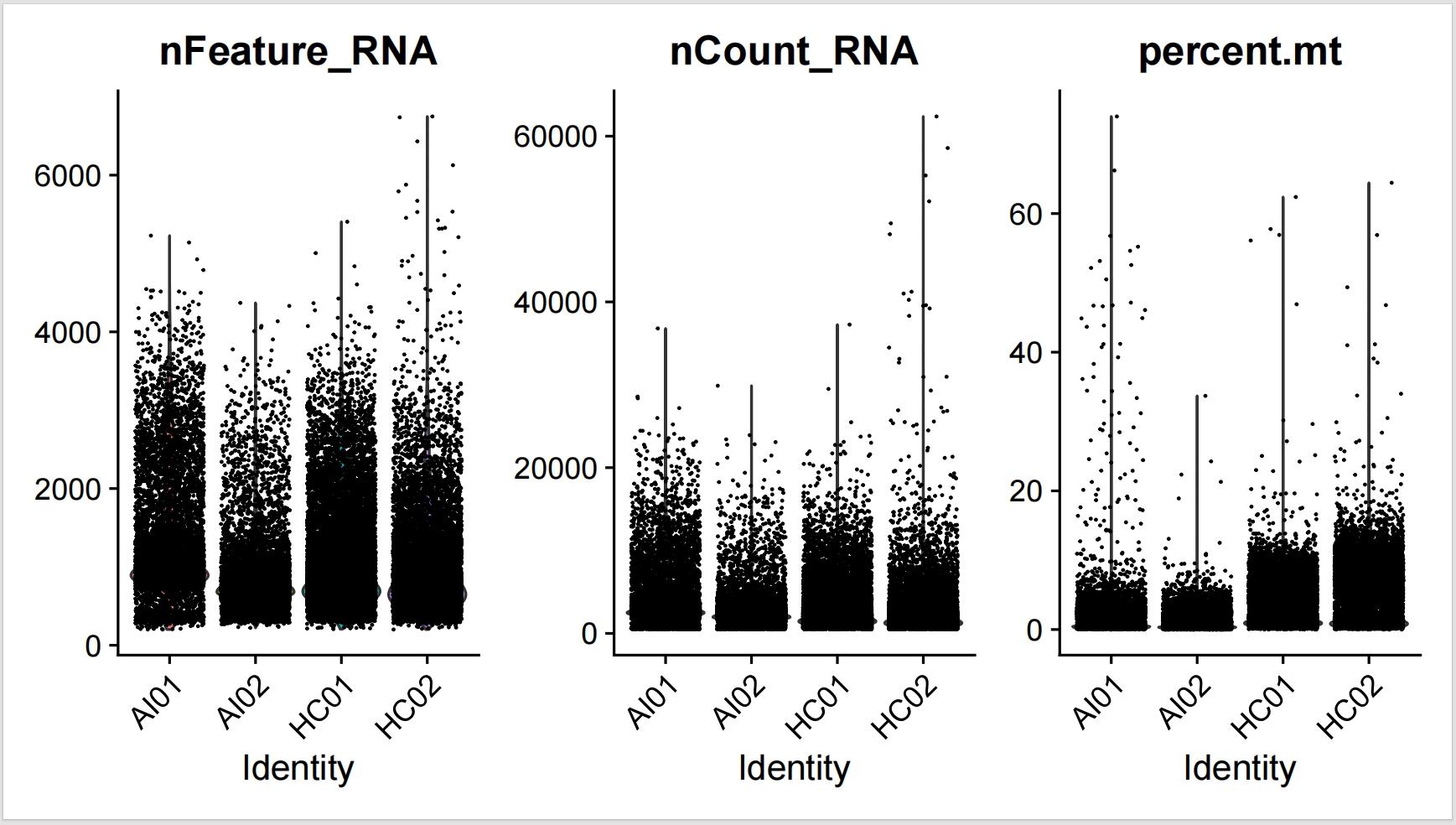


save_path <- "./QC-VlnPlot.tiff"

ggsave(save_path, width = 8, height = 6, dpi = 300)


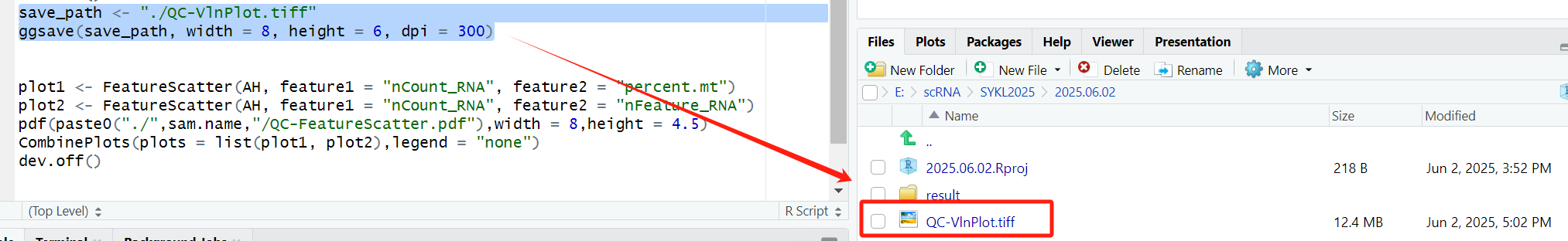


plot1 <- FeatureScatter(AH, feature1 = "nCount_RNA", feature2 = "percent.mt")

plot2 <- FeatureScatter(AH, feature1 = "nCount_RNA", feature2 = "nFeature_RNA")

pdf(paste0("./",sam.name,"/QC-FeatureScatter.pdf"),width = 8,height = 4.5)

CombinePlots(plots = list(plot1, plot2),legend = "none")

dev.off()


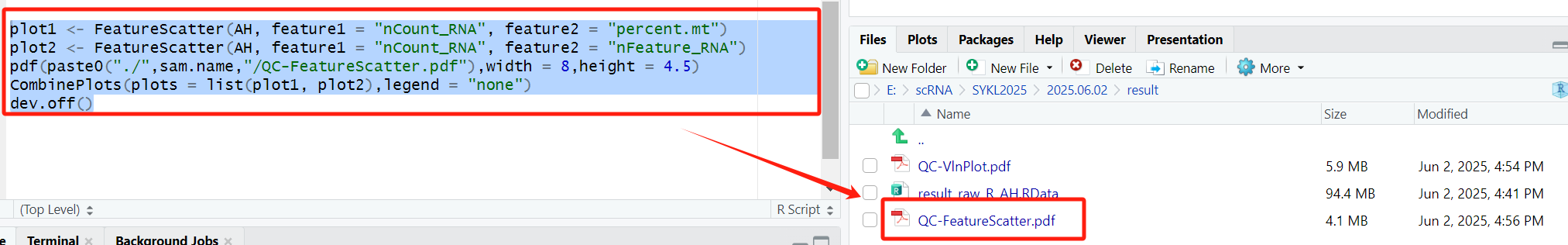


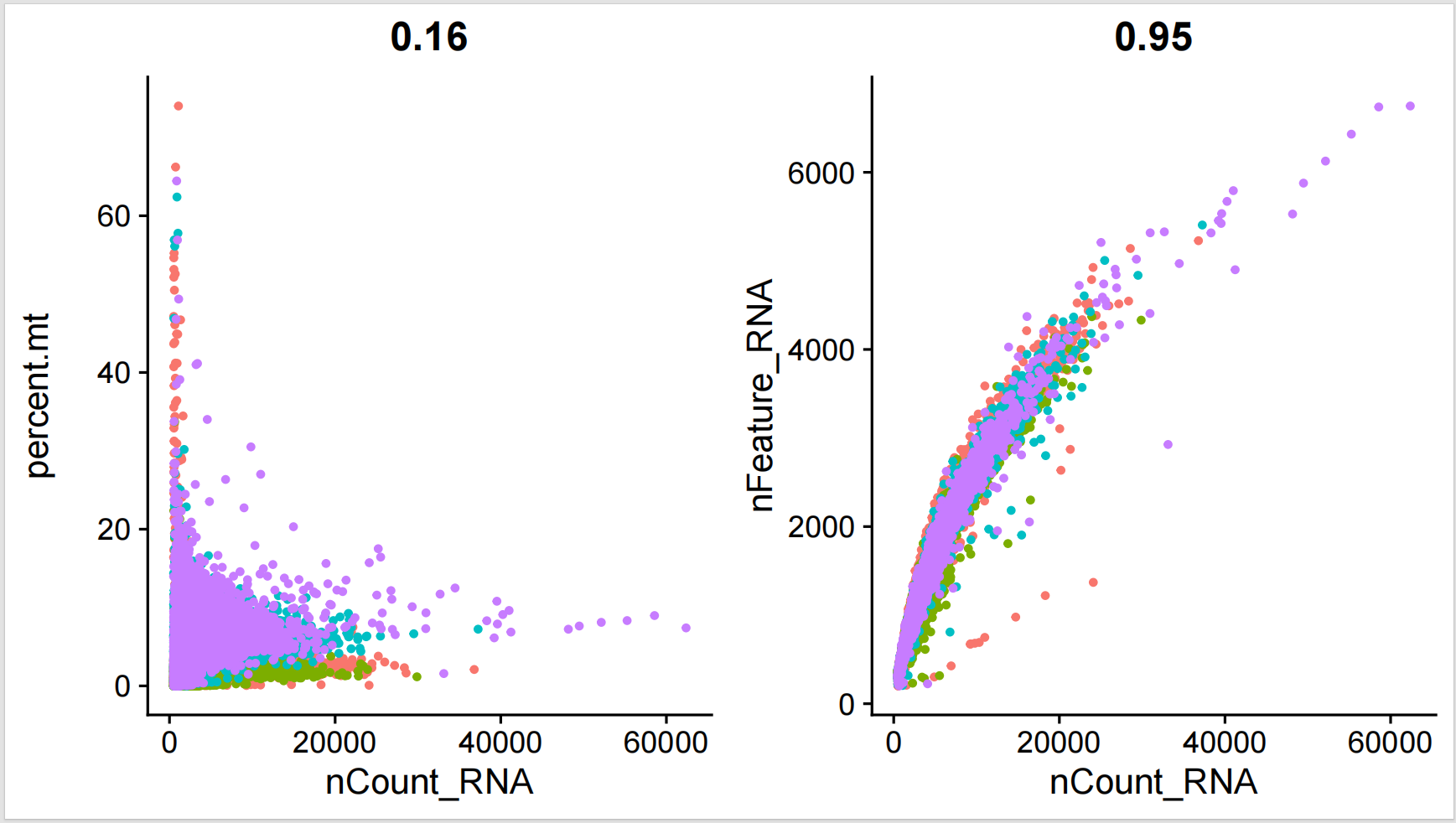


save_path <- "./QC-FeatureScatter.tiff"

ggsave(save_path, width = 8, height = 6, dpi = 300)


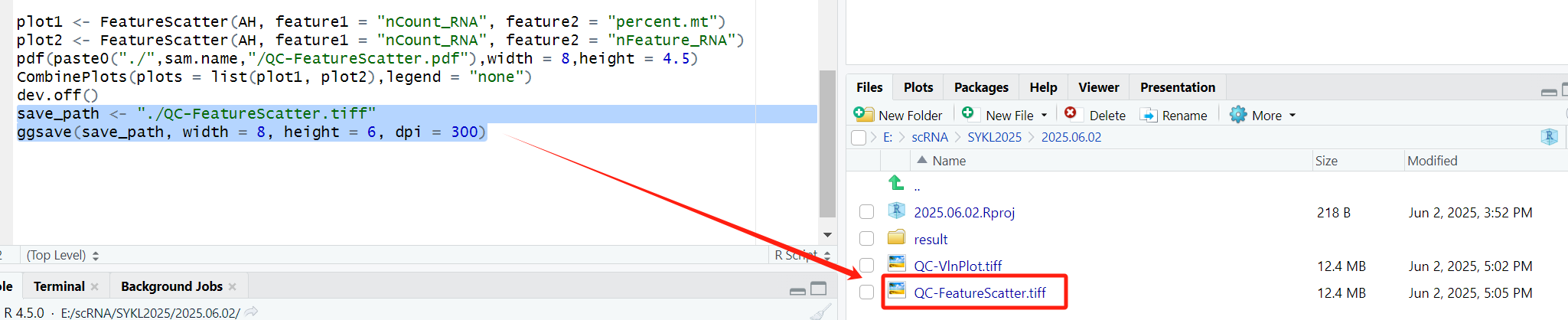


AH<- subset(AH,

subset =

nFeature_RNA > 200 &

nFeature_RNA< 4000&

nCount_RNA < 20000 &

percent.mt < 10)

cat("After filter :",nrow(AH@meta.data),"cells\n")

[table(AH@meta.data$orig.ident)](mailto:table(AH@meta.data$orig.ident))


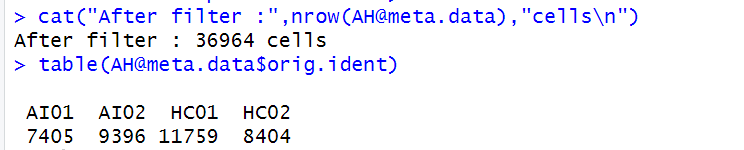


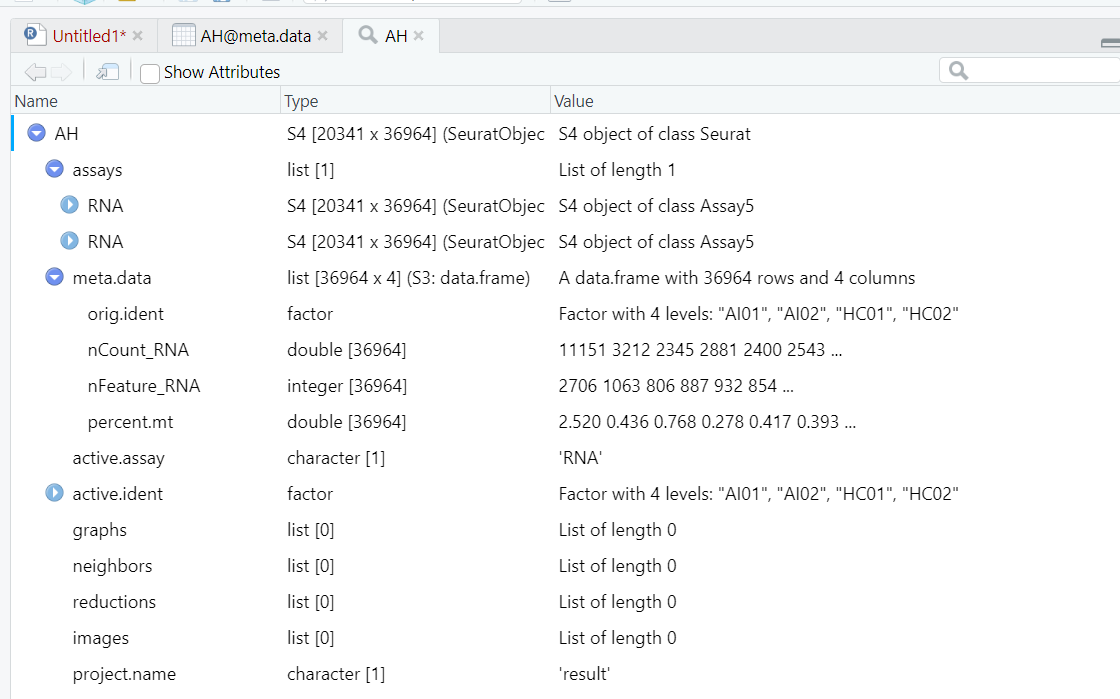


#Directly load data

load("D:/SYKL-DXB/SYKL2025/2025.08.16/result_raw_R_AH.RData")

#Calculate mitochondrial percentage

AH[["percent.mt"]] <- PercentageFeatureSet(AH,

pattern = "^MT-")

#Quality control and cell filtering

AH<- subset(AH,

subset =

nFeature_RNA > 200 &

nFeature_RNA< 4000&

nCount_RNA < 20000 &

percent.mt < 10)

#Normalize data

AH <- NormalizeData(AH,

normalization.method = "LogNormalize",

scale.factor = 10000)

#Identify highly variable features

AH<- FindVariableFeatures(AH,

selection.method = "vst",

nfeatures = 2000)

#Scale data

all.genes <- rownames(AH)

AH <- ScaleData(AH, features = all.genes)

#PCA linear dimensionality reduction

AH <- RunPCA(AH, features = VariableFeatures(object = AH))

#Cell clustering

dim.use <- 1:20

AH <- FindNeighbors(AH, dims = dim.use)

AH <- FindClusters(AH, resolution = 1)

#Perform nonlinear dimensionality reduction

AH<- RunUMAP(AH, dims = dim.use,

do.fast = TRUE)

head(AH@meta.data)

#Add group "Influenza", "Healthy"

AH$group <- ifelse(AH$orig.ident %in% c("AI01", "AI02"),

"Influenza", "Healthy")

#Find differentially expressed features

all.markers <- FindAllMarkers(AH, only.pos = TRUE,

min.pct = 0.5, logfc.threshold = 0.25)

# Correctly create grouped gene list (remove duplicate FCER1A）

all_features_all_cell <- c(

"CSF3R", "FCGR3B", "FPR1", # Neutrophil

"KLRF1", "GNLY", "NKG7", # NK

"CD3D", "CD3G", "CD3E", "TRAC", "CD247","TCF7", # Tcell

"FCN1","MS4A6A", "CD14", # Monocyte

"CD68", "CTSB", "LGALS3", "CTSZ", "IDO1","MSR1", # Macrophage

"CD79A", "MS4A1", "IGHM", "IGHG3", "IGHA2", "FCER2", # Bcell

"CD1C", "IL3RA", "FCER1A", # DC

"MS4A2","GATA2", # Mast

"PF4","PPBP","TUBB1" # Non_Immune

)

# Create corresponding group vector (must match gene count: 3+3+6+3+6+6+3+2+3 = 35）

gene_groups <- c(

rep("Neutrophil", 3),

rep("NK", 3),

rep("Tcell", 6),

rep("Monocyte", 3),

rep("Macrophage", 6),

rep("Bcell", 6),

rep("DC", 3),

rep("Mast", 2),

rep("Non_Immune", 3)

)

# Check for duplicate genes

any(duplicated(all_features_all_cell))

# Define group colors

group_colors <- c(

"Neutrophil" = "#8B0000",

"NK" = "#006400",

"Tcell" = "#0047AB",

"Monocyte" = "#4B0082",

"Macrophage" = "#8B4513",

"Bcell" = "#8B008B",

"DC" = "#008B8B",

"Mast" = "#483D8B",

"Non_Immune" = "#2F4F4F"

)

#  Create corresponding color vector for each gene

gene_colors <- group_colors[gene_groups]

names(gene_colors) <- all_features_all_cell

# Draw dot plot

p <- DotPlot(

AH,

features = all_features_all_cell,

group.by = "seurat_clusters",

dot.min = 0.01,

scale = FALSE,

col.min = 0,

col.max = 3,

cols = c("lightgrey", "blue"),

assay = "RNA",

dot.scale = 10

)

# Extract plot data

plot_data <- p$data

# Assign corresponding group color to each point

plot_data$gene_group <- gene_groups[match(as.character(plot_data$features.plot), all_features_all_cell)]

plot_data$base_color <- group_colors[plot_data$gene_group]

# Calculate transparency based on expression level (higher expression = lower transparency / more opaque）

plot_data$alpha_value <- scales::rescale(plot_data$avg.exp.scaled, to = c(0.1, 1.5))

plot_data$alpha_value <- ifelse(is.na(plot_data$alpha_value), 0.2, plot_data$alpha_value)

# Recreate dot plot using group colors and transparency

p <- ggplot(plot_data, aes(x = features.plot, y = id)) +

geom_point(aes(size = pct.exp, fill = base_color, alpha = alpha_value),

shape = 21, color = "black") +

scale_size_continuous(range = c(1, 10), name = "Percent Expressed") +

scale_fill_identity() +

scale_alpha_identity() +

theme_minimal() +

theme(

axis.title.x = element_blank(),

axis.text.x = element_text(angle = 45, hjust = 1, color = gene_colors[all_features_all_cell],

size = 14),

axis.text.y = element_text(size = 14),

axis.title.y = element_blank(),

legend.title = element_text(size = 14),

legend.text = element_text(size = 12),

legend.position = "right",

plot.margin = margin(t = 60, r = 5, b = 5, l = 5)

) +

labs(size = "Percent Expressed", alpha = "Expression Level")

# Get number of clusters

n_clusters <- length(unique(plot_data$id))

# Calculate center position of each group

group_positions <- data.frame(

group = unique(gene_groups),

start = match(unique(gene_groups), gene_groups),

end = sapply(unique(gene_groups), function(g) max(which(gene_groups == g)))

)

group_positions$mid <- (group_positions$start + group_positions$end) / 2

# Add group labels to the top of the plot

p <- p +

annotate("text",

x = group_positions$mid,

y = n_clusters + 1.5,

label = group_positions$group,

color = group_colors[group_positions$group],

size = 5,

fontface = "bold") +

annotate("segment",

x = group_positions$start - 0.5,

xend = group_positions$end + 0.5,

y = n_clusters + 0.8,

yend = n_clusters + 0.8,

color = group_colors[group_positions$group],

size = 1) +

coord_cartesian(ylim = c(0.5, n_clusters + 2), clip = "off") +

guides(alpha = guide_legend(override.aes = list(fill = "black"),

title = "Expression Level"))

save_path <- "./Dotplot_all_cell_grouped_444.tiff"

ggsave(save_path,

plot = p,

width = 22,

height = 20,

dpi =300)


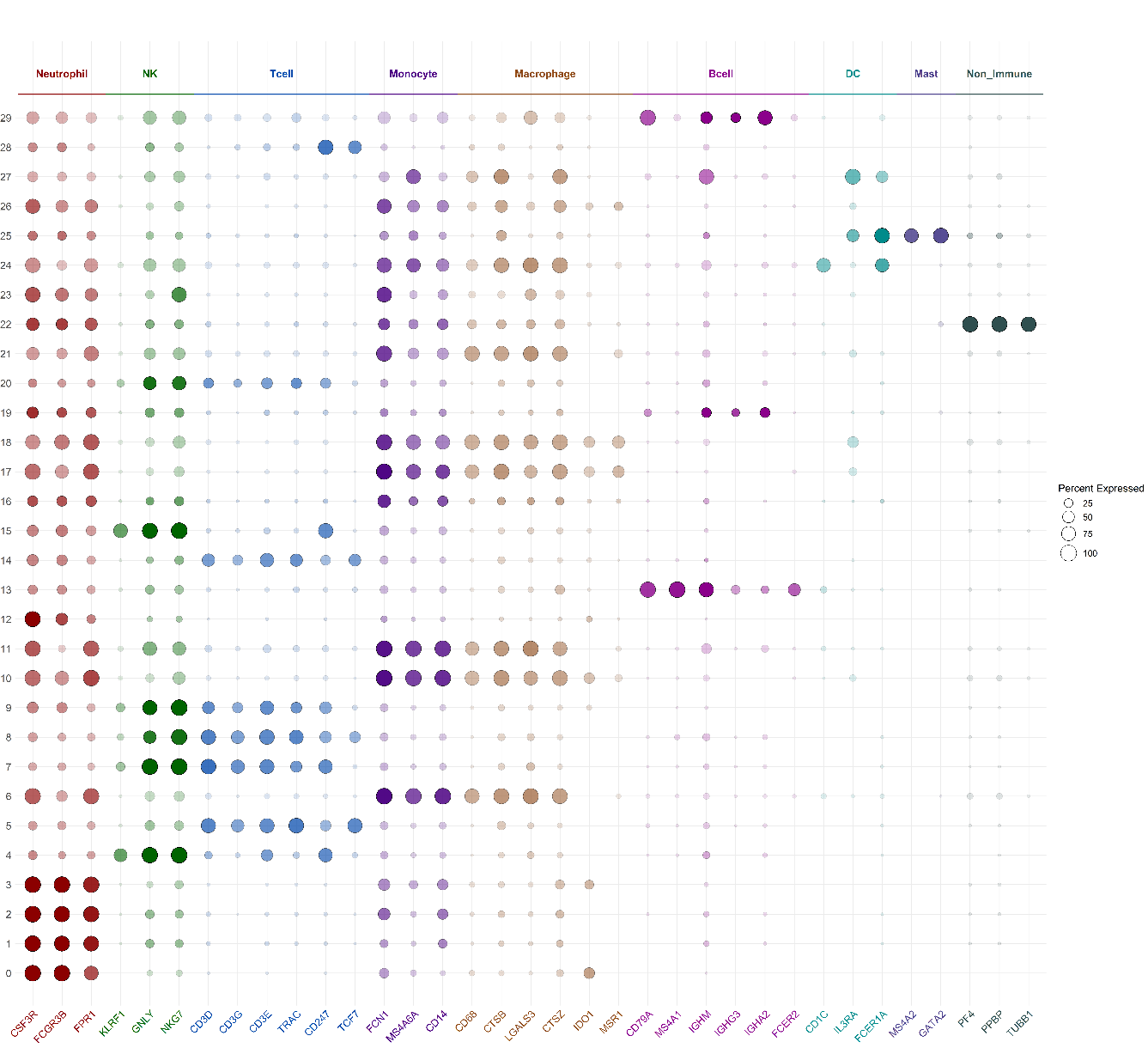


AH$celltype.main<-recode(AH@meta.data$seurat_clusters,

"0"="Neutrophil",

"1"="Neutrophil",

"2"="Neutrophil",

"3"="Neutrophil",

"4"="NK",

"5"="Tcell",

"6"="Monocyte",

"7"="Tcell",

"8"="Tcell",

"9"="Tcell",

"10"="Monocyte",

"11"="Macrophage",

"12"="Neutrophil",

"13"="Bcell",

"14"="Tcell",

"15"="NK",

"16"="Monocyte",

"17"="Macrophage",

"18"="Macrophage",

"19"="Bcell",

"20"="Tcell",

"21"="Macrophage",

"22"="Non_Immune",

"23"="Neutrophil",

"24"="DC",

"25"="Mast",

"26"="Macrophage",

"27"="DC",

"28"="Tcell",

"29"="Bcell")

sub_AH_IA<- subset(AH,

subset = group%in%"Influenza")

sub_AH_HC<- subset(AH,

subset = group%in%"Healthy")

table(AH$celltype.main)

table(sub_AH_IA$celltype.main)

table(sub_AH_HC$celltype.main)


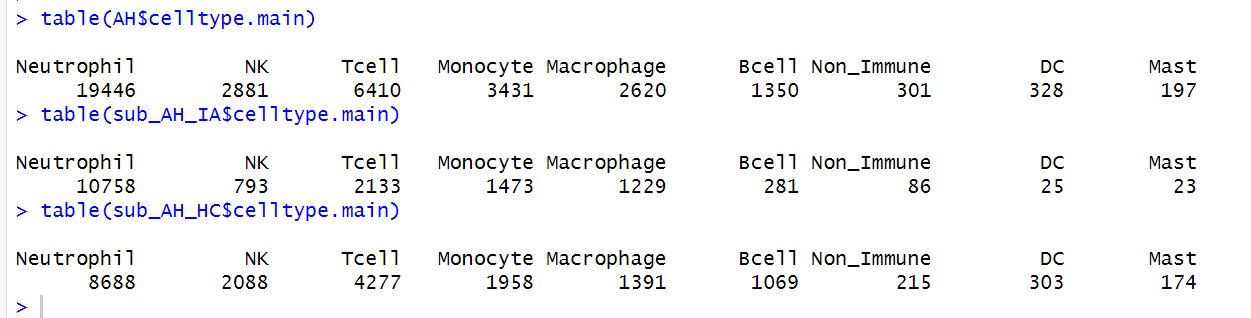


save(AH,file = "./AH_cluster_group_celltype.RData")

DimPlot(AH,

group.by = "celltype.main",

label = TRUE,

pt.size = 0.5,

repel = TRUE) +

ggtitle(" Cell Types")

save_path <- "./CellCluster-UMAPPlot_Group_celltype.main.tiff"

ggsave(save_path,

plot = DimPlot(AH,

group.by = "celltype.main",

pt.size = 0.5,

label = TRUE,

repel = TRUE),

width = 8,

height = 7,

dpi = 300)


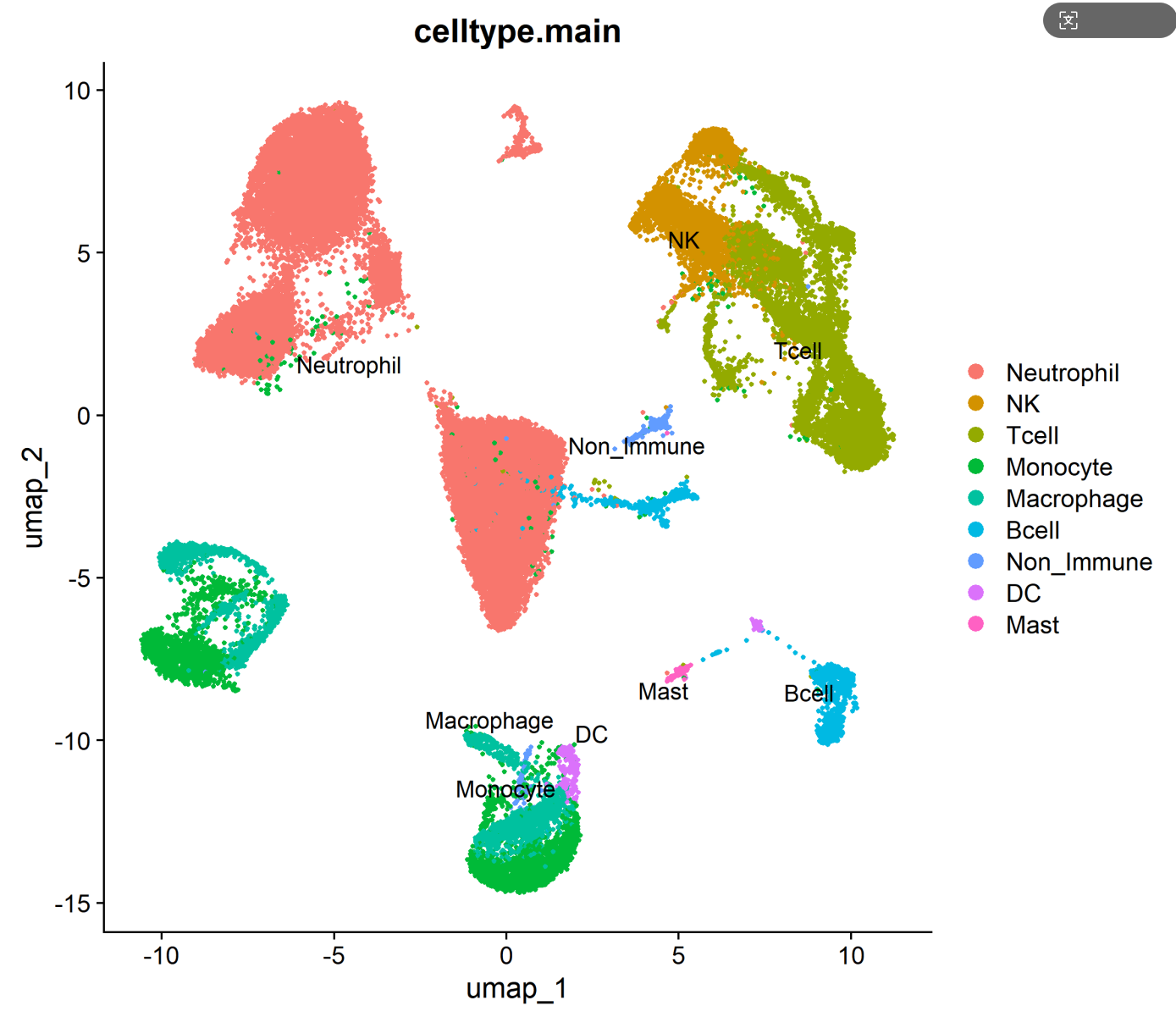


[View(AH@meta.data)](mailto:View(AH@meta.data))

meta_data <- AH@meta.data

plot_data <- data.frame(table(meta_data$group,meta_data$celltype.main))

plot_data$Total <- apply(plot_data,1,function(x)sum(plot_data[plot_data$Var1 == x[1],3]))

plot_data <- plot_data %>% mutate(Percentage = round(Freq/Total,3) * 100)

ggplot(plot_data,aes(x = Var1,y = Percentage,fill = Var2)) +

geom_bar(stat = "identity",position = "stack") +

theme_classic() +

theme(axis.title.x = element_blank()) + labs(fill = "Cluster")

save_path <- "./CellCluster-UMAPPlot_celltype.main_group.tiff"

ggsave(save_path, width = 8, height = 7, dpi = 300)


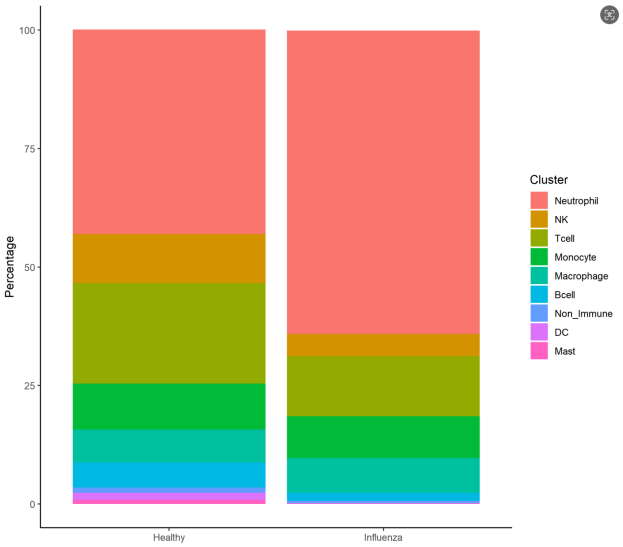


all_features_111 <- c("BAG1",

"CD38",

"MAPKAPK2",

"NOD2",

"IDO1",

"ISG20",

"LGALS9",

"DUSP6")

save_path <- "./Dotplot_all_cell_333333.tiff"

ggsave(save_path,

plot = DotPlot(

AH,

features = all_features_111,

group.by = "celltype.main",

split.by = "group",

dot.min = 0.01,

scale = TRUE,

col.min = -2,

col.max = 2,

cols = c("#8B0000","#006400"),

assay = "RNA",

dot.scale = 10) +

theme(axis.title.y = element_blank()),

width = 10,

height = 8,

dpi = 300)


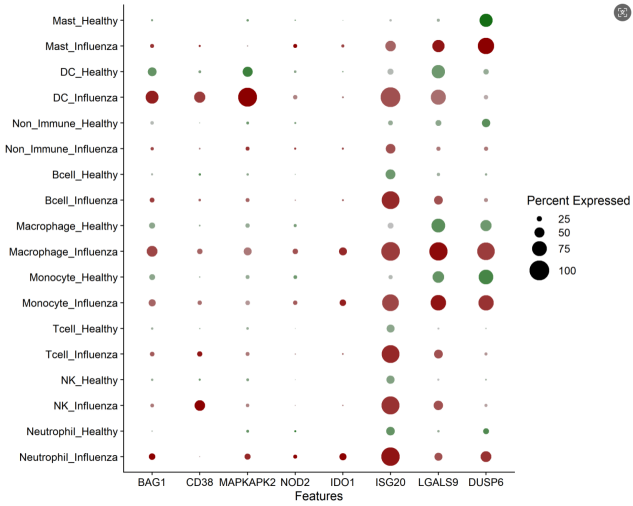

Supplement: S4 Code.R — (DOCX) [file pone.0353259.s004.docx]
