# Supplementary figures and images for: Exploring the mechanism of Shuangyu Granule in regulating immune-inflammatory responses in influenza through UPLC-Orbitrap-MS/MS, GC-MS, and network target analysis
Source: PLoS One. 2026 Jul 27;21(7):e0353259. doi: 10.1371/journal.pone.0353259 (PMC13405112; doi:10.1371/journal.pone.0353259)

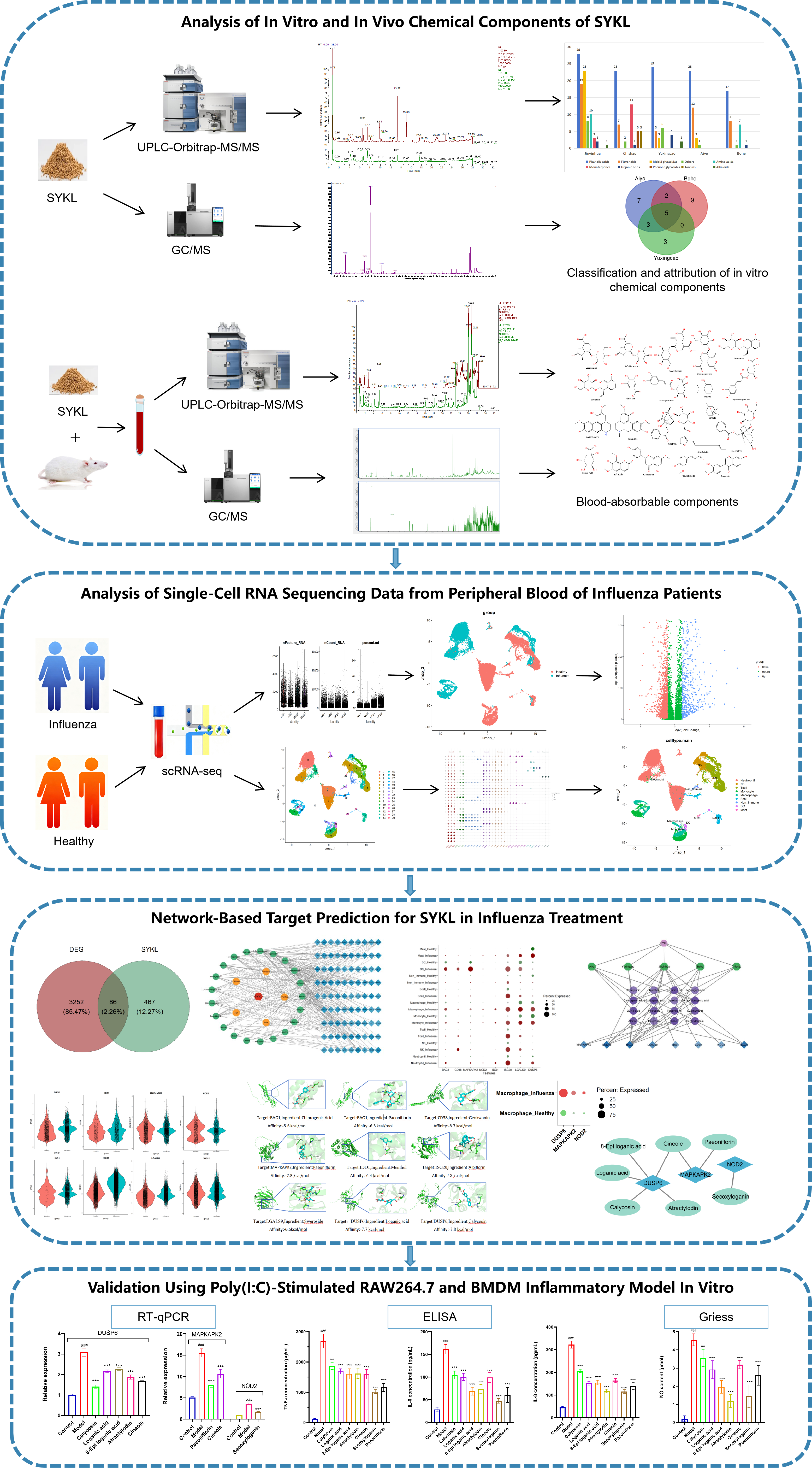

Supplement: S1 Fig — (TIF) [file pone.0353259.s008.tif]
